# Supplementary material for: Reporting of “dialysis adequacy” as an outcome in randomised trials conducted in adults on haemodialysis
Source: PLoS One. 2019 Feb 5;14(2):e0207045. doi: 10.1371/journal.pone.0207045 (PMC6363141; doi:10.1371/journal.pone.0207045)
Supplement: S1 Table — (DOCX) [file pone.0207045.s001.docx]

**Interview guide in English**

Instructions:

- Present yourself as a researcher with an interest in how people define “adequacy of dialysis”.
- Strictly follow order of questions; avoid mentioning biochemical approach in literature until question 3, unless brought up by the patient, to maximize personal input and themes from the patient and avoid suggestion)

Question 1: Do you know the term “adequacy of dialysis” and what is your understanding of this construct;

Question 2: what does this construct of “adequacy of dialysis” mean to you personally as a patient;

Question 3: In literature, “adequacy of diaysis” mainly represents a mathematical (calculated) value based on the amount of urea or one of the other toxins that poison your body if you have severe kidney disease; how do you think about this approach?

Question 4: What do you think about putting “adequacy of dialysis” as represented in literature to reflect “good dialysis”? What would “good dialysis” mean to you?

**Interview guide in Dutch**

Instructies:

- Stel u zelf voor als onderzoeker met interesse in hoe mensen de term “adekwaatheid van dialyse” begrijpen en interpreteren.
- Volg strikt de volgorde van de vragen. Vermijd om voor vraag 3 referenties te maken naar de biochemische definitie zoals die gangbaar is in de literatuur, tenzij de patiënt hier zelf naar verwijst. Dit is noodzakelijk om maximale spontane input en themas vanuit de patiënt te verkrijgen, en om suggesties te vermijden.

Vraag 1: Wat zegt u de term “adekwaatheid van dialyse”?

Vraag 2: Wat betekent het begrip “adekwaatheid van dialyse” voor u als patiënt?

Vraag 3: In de vakliteratuur wordt adekwaatheid van dialyse meestal beschreven door een wiskundige (berekende) waarde die gebaseerd is op de verwijdering van ureum of een andere afvalstof die opstapelt bij mensen bij wie de nieren niet goed werken; wat denk je over een dergelijke aanpak?

Vraag 4: Hoe denk je over het concept dat met adekwaatheid van dialyse zoals het beschreven is in de literatuur, ook “goede dialyse” bedoeld wordt? Wat versta jij onder “goede dialyse”?
