# Supplementary material for: Reporting of “dialysis adequacy” as an outcome in randomised trials conducted in adults on haemodialysis
Source: PLoS One. 2019 Feb 5;14(2):e0207045. doi: 10.1371/journal.pone.0207045 (PMC6363141; doi:10.1371/journal.pone.0207045)
Supplement: S2 Table — (DOCX) [file pone.0207045.s002.docx]

Interview patient 1

S Oké. Ik zou anders eerst willen vragen dat u gewoon een beetje verteld hoe dat u hier
terecht gekomen bent, met andere woorden hoe lang heb je al last met de nieren, hoe is heel dat proces geweest? Een beetje over uzelf vertellen zo.

P Ja wanneer ben ik daar eigenlijk mee begonnen? 2010 zal dat geweest zijn. In 2010 heb ik
 die nieren geblokkeerd.

S Ja en weet u hoe dat dat gekomen is?

P Neen, ik was aan de zee en plots kreeg ik mijn schoenen niet meer aan, ik kreeg mijn
 broek niet meer dicht en ik ben maandagochtend naar huis gegaan. Dan ben ik bij de
 huisarts gegaan en hij heeft mij doorgestuurd naar hier in X. Dan heb ik hier
 14 dagen geweest en hebben ze mij ondersteboven gekeerd, want ik had nog nooit in een
 ziekenhuis binnen geweest. Ik was 50 jaar en dan hebben ze geconstateerd dat ik maar 1
 nier had. Dat wist ik niet.

S Ah u heeft maar 1 nier?

P Ja, dat hebben ze dan gezien. Mijn lever was een beetje verstoord maar dat is allemaal in
 orde gekomen, ik drink niets meer van alcohol of niets. Vroeger heb ik wel redelijk, redelijk
 wel gedronken dat moet ik nu echt toegeven. Maar sindsdien is dat praktisch niets niet
 meer. Ja want normaal gezien volgens dat die dokter gezegd had, zou ik hier niet meer
 gezeten hebben. Het was heel, heel… Ja alles was vergiftigd door die alcohol. Dan heb ik
 toch nog tot begin van dit jaar, hebben we dat kunnen volhouden. Die nier werkte dan
 terug, die is terug opgestart en die werkte nog voor 50 % en dan een 6 maand later was
 dat maar 40 niet meer. Een beetje later was dat nog minder en op een zeker moment is die
 stabiel gebleven op 30. Maar ja ik moest steeds bij dokter komen alle 6
 weken en ze zei dat het zakt, het zakt. Regelrecht naar de dialyse. Wel en van in februari
 ben ik hier.

S Maar bent u in 2010 dan tijdelijk gedialyseerd?

P Excuseer?

S In 2010 met uw vergiftiging hebben ze u dan gedialyseerd?

P Neen, neen.

S Dus nooit gedialyseerd toen? Zo net ontsnapt, ze hebben een stukje gerecupereerd.

P Ja, zo met medicatie.

S En hebt u op voorhand goed uitleg gekregen?

P Jaja van M, dat blonde meisje dat hier loopt.

S Ja oké.

P Ja want ze hebben dan een fistel gemaakt hier over 3 jaar. Dat is 3 jaar dat ze die fistel
 gemaakt hebben. Dat was een voorbereiding omdat ze wisten dat ik hier toch ging moeten
 komen.

S Oké en hoe vergaat het u zo, want het is nog niet zo lang natuurlijk?

P Ja nee, dat is een hele aanpassing natuurlijk want ik ben zelfstandig.
 Moet ik ook zien, ik heb wel veel hulp van, dat is mijn buurman. En mijn vrouw die
 heeft haar werk veranderd, die werkt nu in de namiddag in plaats van de voormiddag de
 dagen dat ik hier zit.

S En wat doet u precies?

P Garage en carrosserie.

S En?

P Ja dat valt mee. We regelen en schikken dat we in de namiddag het werk aanpakken in
 plaats van maandagvoormiddag en de woensdag en vrijdag. Op vrijdag ben ik toch nooit
 thuis al van vroeger al. Vroeger trok ik donderdagavond naar de zee en kwam de
 maandagmiddag terug, maar nu kan ik pas vrijdag vertrekken, vrijdagmiddag.

S Ja een dagje minder met uw dialyse.

P Ja ik zit de vrijdagochtend hier.

S En hebt u ooit een andere optie, hebben ze u ooit andere opties gegeven. Buikspoelingen
 is dat ooit een optie bij u geweest?

P Ja ze hebben dat hier allemaal uitgelegd of een katheter of het zelf doen. Ja ik heb
 gekozen voor hier, ik wist niet wat dat was. Het meeste dat ze zetten zeker? Maar dat is
 dus elke keer in die ader dat ze steken.

S Ja valt dat een beetje mee?

P Op de duur… Maar ja.

S Niet te veel pijn?

P In het begin heeft dat wel een beetje pijn gedaan maar nu gaat dat wel. Dat ‘gangske’ is er
 al, maar toch word je dat nog altijd gewaar. Zoals de bovenste hier, daar raakten ze niet zo
 goed in en dan moeten ze herbeginnen. Ik ben met Pasen dan zijn we een maand in
 Spanje geweest, want ik ga regelmatig naar daar. En daar heb ik dat ook moeten laten
 doen. Dat is goed meegevallen echt.

S En vindt u dat u leven voor de dialyse en alle dingen die u toen deed en nu?

P Dat is een verandering. Dat is een hele aanpassing.

S Maar u bent tevreden met de dingen die u nog kan doen?

P Ja tuurlijk, tuurlijk. Ik moet nog iets verdien, ik ben maar 58 jaar.

S En gaan ze u eventueel ook uitwerken voor transplant ook of hebben ze er niets van
 gezegd?

P Ja daar heeft ze eens over gesproken maar dat is niet voor direct zeker? Je moet eerst een
 jaar aan dialyse zijn.

S Ja en eventueel ook afhankelijk van welke andere ziektes die u eventueel al heeft gehad.

P Niets. Niets. Ik zei het ik was 50 jaar en ik was nog nooit in een ziekenhuis binnen geweest,
 meestal juist voor iemand te bezoeken.

S Het onderzoek dat wij doen is om te proberen wat mensen belangrijk aan dialyse vinden
 wat meer in kaart te brengen. Vroeger werd er meer gekeken vanuit het medisch standpunt
 en enkel naar de machine en de zwart-wit cijfers, maar uiteindelijk is het de patiënt die het
 ondergaat. Om de dialyse dus iets te maken dat zowel medisch goed is maar ook voor de
 patiënt de dingen die belangrijk zijn. Daar zijn wat studies naar gedaan en 1 van de dingen
 die belangrijk was voor patiënten was “adequacy of dialysis”. Nu is de vraag als ik dat
 woord zeg tegen u, zegt u dat iets?

P Nee niets.

S Dat is natuurlijk ook een Engelse term en wat moeilijke term. We kunnen het in principe
 vertalen letterlijk naar “de adequaatheid van dialyse”. Als ik dat zou zeggen tegen u, wat
 denkt u dat dat zou inhouden?

P Ja dat er andere organen zouden kapot gaan of zoiets? Zoals u hart of.. Ja ik heb het
 vorige week nog gevraagd maar dat hangt af van het vocht dat in uw lichaam zit.

S Ja, nu het is inderdaad een moeilijke term dus daarom dat we het proberen te vertalen naar
 wat meer mensentaal en daar zeggen ze “een goede dialyse”. Dus de vraag is een beetje
 wat een goede dialyse voor u is?

P Dat de mensen hier allemaal vriendelijk zijn.

S Ja iets anders ook nog?

P Het moet gebeuren hé anders ga ik dood.

S Is dat iets waar dat u.. Het is moeilijk uit te leggen. Dat is een opmerking die veel mensen
 maken, maar is dat iets waar u in het begin over heeft nagedacht over het niet te doen? Of
 dat is zelf nooit iets..?

P Ja je kunt er niet aan uit hé? Ofwel sterf je ofwel vergiftig je jezelf.

S En zijn er voor u dingen dat u zegt van ja kijk ik heb geen andere keuze ik moet dat doen of
 ik sterf. Nu is het 3 keer 4 uur per week veronderstel ik, is er een bepaald iets waarbij u zou
 zeggen van kijk zou het zo en zo zijn dan zou ik het er niet meer voor over hebben? Stel
 dat het elke dag 4 uur zou zijn?

P Ja dat zou iets anders zijn natuurlijk. Maar ja. Ik ga er altijd van uit als het moet dan moet
 het hé?

S Ja. Er is niet zo een grens mentaal voor u dat u zegt van: “Ja oké ik weet ik heb dat nodig
 om te overleven, maar ik wil ook nog iets hebben van mijn leven los van mijn dialyse”. Want
 daar doe je het uiteindelijk voor het leven van u dialyse te kunnen hebben. Er is geen grens
 dat u zegt van: “Ja tot daar wil ik gaan, maar ik wil niet meer dan dat doen voor in leven te
 kunnen blijven”?

P Dat ik een andere nier krijg.

S Ja dat is natuurlijk beter, maar stel dat ze u elke dag zouden moeten dialyseren. Zou u dan
 zeggen van: “Oké ja als het moet dan moet het”?

P Ja als het moet dan moet het.

S En als dat nu elke dag 12 uur zou zijn?

P Dat is iets anders ja.

S Dat is moeilijk hé?

P Ja dat is zeker.

S Oké en dat u zegt van ik heb niet veel keuze als ik zeg van een goede dialyse, maar kunt u
 merken of een dialyse goed is? Merkt u iets?

P Nee. Nee want ik voel mij nog steeds hetzelfde als over 10 jaar. Want toen ze zei dat ik
 naar dialyse moest, zei ik van “Waarom?”. Ja u nier is kapot en u creatinine staat veel te
 hoog. Ik zeg: “Ja maar ik voel dat niet”, maar het schijnt dat dat juist is dat je dat niet voelt?

S Neen heel soms, sommige mensen wel.

P Ik ben niet moe, ik ben vanmorgen om 5 uur 30 opgestaan en om 7 uur zat ik in mijn auto
 om naar hier te komen, rond 6 uur. ’s avonds ga ik wel niet zeggen om 9 uur ben ik in mijn
 bed. Als ik in Spanje ben dan ben ik daar ook om 9 uur in mijn bed. Vroeger ja als ik muziek
 speelde ja, dan was dat natuurlijk 4 - 5 uur als ik naar huis kwam. Maar dat is “temps
 passé”, dat is gedaan. Dan was ik veel jonger ook.

S Het vroeger gaan slapen wijt u niet aan uw dialyse? Of aan uw nierproblemen?

P Dat denk ik niet? Als ik gegeten heb en ik zit in de zetel kan ik wel in slaap vallen. Of
 s’avonds als ik televisie kijk om 7 uur of iets. Maar ik denkt dat andere mensen dat ook
 hebben als je de hele dag rondgelopen hebt.

S Zijn er dingen dat je denkt van dat is anders geworden in mijn leven en dat is van de
 dialyse?

P Nee.

S Nee? Oké. Stel nu u mag een wens doen. Er is iets zoals een geest in een lamp en u mag
 1 ding wensen om aan dialyse te veranderen, maar je kan niet te vragen om los te zijn van
 dialyse. Is er iets dat je zou zeggen van?

P Ja een andere nier krijgen!

S Ja maar dat is ook weg zijn van dialyse dus iets aan dialyse zelf veranderen? Dus nu is
 het je komt naar hier met de auto, zoveel tijd onder de baan dan wordt je aangekoppeld,
 wordt je geprikt en dan draait de machine. Dan zou u bloed gezuiverd moeten zijn en wordt
 je afgekoppeld. Heel dat proces van u dialysesessie, moest je iets kunnen aanpassen?

P Dat het sneller zou kunnen?

S Bijvoorbeeld.

P Dat ze in plaats van 4 uur op 2 uur zouden kunnen dialyseren.

S Je mag gelijk wat.. de opties waren eindeloos. Is dat dan ook zo voor u dat tijd 1 van de
 meest vervelende zaken is aan dialyse?

P Ja tuurlijk. Je zit hier maar hé.

S Zijn er andere dingen die je zegt van dat vind ik lastig of vervelend aan dialyse waar u op
 voorhand misschien niet bij nagedacht had?

P Neen, want ik wist niet wat dat was hé? Maar het valt goed mee, de mensen zijn hier
 vriendelijk en plezant. Al bij al dat gaat sneller dan. Kijk ik zit hier nu al 2 uur.

S Ja en waar houdt u zich dan vooral mee bezig dan?

P Oh de krant en mijn mp3 luisteren, beetje praten met M dat is ook mijn
 buurman van bij ons thuis.

S Ook al? Dat moet nu wel lukken dan.

P Ja hij let op mijn hond als ik weg ben en alles. Ja hé M? Hij heeft de sleutel van mijn
 huis zelfs.

S Dat is wel handig.

P Ja er is altijd wel iets te vertellen. Juist dat we nog niet samen gaan vissen, anders zouden
 we niets meer te vertellen hebben.

S Hij gaat vissen zeker?

P Ja hij gaat vissen. Ik doe niets meer als hobby. Vroeger heb ik elke dag muziek gaan
 spelen, alle kanten waar ze mij nodig hadden.

S Welk instrument?

P Trompet.

S Ah ja blaasinstrumenten.

P Maar ja dat is nu wat. Vanaf dat ik dat hier voorgehad heb.

S Van 2010 al? Dat je al gestopt bent?

P Ja. Af en toe pak ik die trompet nog eens vast voor nog eens, maar ja.

S Dat is niet hetzelfde.

P Dat is hetzelfde niet hé.

S U zegt dat u vrouw haar werk ook wat heeft aangepast, hebben jullie ook kinderen?

P Nee.

S Nee en u woont waarschijnlijk alleen samen met uw vrouw. U hebt geen ander gezin
 inwonend?

P Neen.

S Hoe heeft de dialyse u gezin ook zo wat.., dus los van het werk, ook de gewone
 dagdagelijkse dingen wat aangepast of..?

P Ja tuurlijk zoals ik wou graag naar de zee, wij vertrokken op donderdagavond. Nu gaat dat
 niet meer nu moet ik vrijdagmiddag vertrekken. Anders konden wij maandag op de middag,
 rond een uur of 10 vertrekken. Nu moet ik zeker als ik wil in de zomer moet ik daar om 6
 uur vertrekken de zondagavond. Dat moet je allemaal. Je bent daar constant mee bezig,
 dag en nacht.

S Hebt u trouwens ooit al een slechte dialyse gehad?

P Neen.

S U heeft zich nog nooit slecht gevoeld aan de dialyse?

P Neen.

S U bent nog niet zo lang gedialyseerd, maar voor u is elke dialysesessie hetzelfde?

P Hetzelfde ja.

S En er moet ook geen vocht af zie ik bij u?

P Af en toe wel.

S Ah ja en voelt u daar een verschil in?

P Ja dan voel je je een beetje lichter.

S Letterlijk?

P Ja dat is echt. Of wel is het de gedachte, maar toch als je u gaat wegen kan het 2 kilo
 schelen.

S Ahja, ik zie nu dat er geen vocht ingesteld is.

P Awel ja ik weet het niet, ik ken maar juist de bloeddruk. Dat was vroeger ook niet goed, dat
 is nu ook verbeterd. Praktisch goed.

S En denkt u dat dat met de dialyse te maken heeft of..?

P Mijn bloeddruk heeft altijd hoog gestaan. Ik heb altijd rond de 20 gehad, maar ja daar werd
 ook nooit naar gekeken. Nu heb ik daar pilletjes voor.

S Ja en u medicatie werkt?

P Ja in het begin niet maar op de duur is dat beginnen werken. Zoals ook die plaspillen dat ik
 moet nemen voor dat water he. In het begin zei ik het ik moest daarvoor niet meer van
 gaan plassen, maar nu wel. Nu ondervind ik dat precies toch wel ja. s’nachts 3 - 4 keer
 opstaan.

S Dat is al de moeite ja.

P Ja dat zijn allemaal van die nevenwerkingen. Maar ja als dat niet anders kan. Er zijn zoveel
mensen, want vroeger dacht je daar niet aan. Je wist dat wel dat er iemand niercrises
kreeg of een keer stenen in hun nieren maar van dialyse. Ik wist vroeger niet eens dat dat
bestond. Je hebt daar ook zoveel soorten in. Bij ons in de gemeente, die doet dat thuis. Die heeft schijnt schaamte om hier te komen zitten. Ik weet eigenlijk niet
waarom. Ik vind toch dat je hier altijd onder controle staat, als er hier iets piept of zo. Als je
dat thuis voorhebt, dan sta je daar wel.

S Iedereen zo zijn eigen ding.

P Ja je bent misschien wel vrij overdag, als je dat ’s nachts kan doen. Maar ja slaap je daar
 dan mee, dat weet ik niet.

S het feit dat je dat hier doet, zo onder controle en de dokters komen langs en de
 verpleegkundigen, denk je veel na over je eigen dialyse? Of is dat meer van het moet en
 voor de rest..?

P Het moet hé.

S Laat ik het gewoon in de handen van de rest.

P Ja.

S Heeft u in het begin veel gepiekerd of slecht geslapen?

P Neen. Ik heb mij daar bij neergelegd, je kunt niet anders hé? Gelukkig dat dat bestaat.
 Zoals in Afrika die arme landen zo, wat gebeurt daar hé? Daar sterven de mensen. Ze
 zullen daar een andere naam aan geven en zullen dat zo niet zeggen misschien maar…

S Maar het resultaat is hetzelfde. Nee oké goed. Dankjewel.
 Het is altijd… ***Stop opname***

Interview Patiënt 2:

**S** Oke euhmm ik ga ook wa notities maken [P: ja] tijdens euh dat we aan het praten zijn zodanig dat als er iets interessant is da je nu zegt en ik wil u niet onderbreken dan kan [P: jaja] dan kan er eventueel er nog op euh kan terugkomen.

**P** Ja ja ja ja.

**S** Euhmmm dus het meeste hebben we al gezegd dernet dus euhh ik ben S,

Ja.

**P** Ja da mag waarom [S: oke] .. niet?

**S** Euhmmmm dus weet ik ben verbonden aan de dienst van X hé?

**P** Ja.

**S** We zijn bezig met onderzoek te doen om te zien hoe dat patiënten zelf dialyse ervaren en dus niet puur het medische [P: jajajaja] de dokters de kant hé maar ne keer van de patiënten, hunne kant van de patiënten. Euhm en dan na het onderzoek als we wa resultaten hebben als u wilt kunnen we ook altijd zeggen of wat daar uitgekomen is.

**P** Jajajaja.

**S** Dus euh we gaan ook moesten we bepaalde dingen zeggen die zeer interessant zijn en we willen da achteraf gebruiken als onderzoek gaat daar nooit iemand ooit weten dat u da geweest bent.

**P** Jajajajajajomaja das gien probleem de rest. Ja.

**S** Euhm begint anders een keer me wa te vertellen over u zelf en euh hoe da euh u nier.. u nierziekte en hoe dat u in dialyse gekomen bent.

**P** Ja ik ... ik em al joeren. Ik ben al joeren diabetisch. Naart schijnt is da al slecht voor de nieren [**S**: ja] Toen emmek euh voor een twee jaar en half emmek kanker gehad en kem chemo gehad en vandoor is da beginnen vrie na benieden te goen en dan kwammet uit van mijn nieren allez ja .. en aan den andere kant bennekik content da da bestoet da ge dingen et want khad er misschien al verre van tussen geweest dus kem mij daar ..

**S** Dialyse wil je zeggen?

**P** Jaa, het is wel een ongemak me daje hier drie keer in de week moet komme ma had da neu content zen da da bestoet.

**S** Ja. En bent u euhmmm altijd hier geweest in Xof euh..?

**P** Baja want et is het kortste bij he.

**S** Ja dus alles is hier gebeurd en ook u eerste dialyses alles vanaf hier vanaf het begin altijd in [**P**: ja] X geweest? En altijd hier op dezelfde hemodialyse?

**P** Nee

**S** Nooit andere dialyse dingen gedaan? Nooit thuis euhmm?

**P**  Niejenieje [**S**: of buiskpoelingen] nieje nieje .. Nieje ma ik doen da voor .. van eind november.

**S** Dus u bent nog (S tegen andere persoon: Ah dankuwel)

**P** Ja.

**S**  Dus u bent eigenlijk nog maar recent gestart *(praten samen)*?

P Ja.Ja jajajaja.

**S** En hoe ervaart u de dialyse?

*(korte stilte)*

**S** Wa voelde daarvan?

**P** *(zucht nadenkend)* Het voelen? Ja eh ge krijgt veel..veel niet. Kemkik daar gene last van da demmek. Aahja ik wozekik zelfs verwonderd da..als ik ier gekommen ben op visite. Da ze mij zeide dak direct aan de dialyse moest met dat (korte pauze) da mijn bloeduitslag ni goe meer was. En kwas daarvan verwonderd maja aan den andere kant ja als da nodig is is da nodig ennn..

**S** En had u.. Had u bepaalde klachten op da moment?

**P** Ni speciaal.. Ni speciaal ma jah op den langen duur weet ge ni mi wa dage hed he. Ik was veel moe enn.. kortademig da was ik oek.

**S** Ja.

**P** Ma da was alleen da van ni. [**S**: Ja] Da was puur van mijn hart wast.. Ondertussen emme ze mij al *(korte pauze)* in de moand januari eeuhh ik em over.. een goe joar en half emmik ne stent gestoken geweest.. En nu over in de moand januari emme zer mij drei nog gestoken. Doarmee emmik er vier he. Awel ja tis al de moeite ist.

**S** Ja.

**P** Allezja euh.

**S** Doen ze hun werk

**P** Awel ja kzen gene, bennekik toch altijd content da kik hier nu kunne kome, allez da kik hier nu da ik hier toegekomen ben da da helpt.

**S** Ja

**P** Maar last.. last dan hebik noa begot ni van.

**S** En euhmm dus we hebben .. der zijn al studies gebeurd vroeger he [**P**: ja] zelfs Engelstalige studies en [**P**: ja] in één van die studies zegge.. zeggen ze één..één van de factoren da ze altijd bekijken is de "Adequacy of dialysis". Das een Engelse term, ge kunt da ni goe vertalen.. als we da zouden vertalen is da "adequaatheid van u dialyse". *(korte pauze)* Moestik die term zeggen aan u [**P**: ja] wa.. wa zou da voor u euh wa houdt dat in of zegde "grohja.." *(korte pauze)* "daar kan ik nu ni direct euh.."

**P** Jaja.

**S** Wa maakt er voor u dan da een dialyse goed is?

**P** Ja het feit *(lange pauze)* Ik weet als ge.. als u nieren nieje werken dage u vergiftigd [**S**: uhu] u eigen lichaam vergeeft en euh bennekik eigenlijk content da da bestoat da ze mij kunnen helpen zo. Ma kmoekik nu voor de rest van mijn leven drie keren in de week kommen.. Awel da moeije tein bijpakken moeije .. Als ge wil leven toch..

**S** En nu u zegt dat da vervelend is da. da drie keer per week euh dialyse komen..

**P** Bwa vervelend.. *(korte pauze)* Enerzijds jaa.. als ge.. ge kunt niks anders meer doen, als ge gepensioneerd allez normaal gezien al gepensioneerd bena twintig jaar op euh [**S:** ja] een overgangspensioen dakem. *(korte pauze met zucht)* Ja moekik ni meer werken en moe kik, ofdakik nu hiere zit of thuis in de zetel zit da blijft nu toch allemaal iender? Tenigste da je thuis moet koisjen dasal.

**S** En euhm bent u getrouwd?

**P** Geweest.

**S** Geweest.. Alleenstaand?

**P** Neeje bij, me mijne zoon.

**S** Met u zoon.

**P** Me mijne zoon is thuis nog. Hij is al .. Hij wordt van tjaar vier .. vierendertig jaar. Das nogaltijd thuis dus. awel ja..

**S** Hij blijft wa bij de papa plakken?

**P** Awelja..voort ogenblik heeft em ni veel tijd en hij.. Hij moe werken altijd nu.

**S** En als u.. u tijd nu bekijkt me u tijd voor daje aan dialyse was. Moete veel dingen laten azo dan naar u tijdsbesteding naar de dingen die ge vroeger deed die ge nu laat vallen?

**P** Bwa ik doenekik mijn werk thuis nog een beetje gelijk dak kan, kem een poetsvrouw, dat hemmik, en kem iemand die komt voor de strijk te doen. Nu nog maar onlangs ze, van int begin van joar hemmek maar voor de rest moestik alles zelf doen: wassen en strijken en al. Maar nu benik al content dak dade euh ni meer moet doen. [**S:** Ja] Moar der woare veel werken dak ni meer kon doen oek. Kem..Zemme allez met den tijd gezeit me dak vrie last had aan menne rug. Awel ja en den tussenin emmik al een poar keer geoppereerd geweest aan menne voet oek. Zemme zelf mijn tenen geamputeerd emme ze. Kort erna als ze mijne teen geamputeerd emme enkele maandennadie emmek dan kanker gehad. Allez ja.. Ik hem al veel veel meegemaakt emmek, mo den als ge doar tegen kunt vechten en ge ge zijt erdeure gekommen dan kunde zo goed na dialyse kommen oek hé. [**S:** Ja] Kunde.. als ge.. als ge u leven meu..beu allez moe zijt of der deur zijt jah da kan ik wel aanpakken da je u laat gaan, maar alst er iet aan te doen is.. Kvind da ge er veu moe vechten.

**S** Dus.. En denkt u dat dat iets is da de.. da de meeste patiënten wel u mening delen of denkte da er sommige mensen het er veel lastiger mee hebben of euh..?

**P** Ja *(korte pauze)* Ja hoe hoe gaakt zeggen hé. Als ge zo gelijk als ekik nu. Als ik zo.. als ge zolang gevochten het veu in leven te kunnen blijven me de kanker enal woar dak vrie .. vrie slecht geweest hem in het begin. Maar ik heb alles loaten stoen hemmik van die dinge .. allez ja.. gelijk als da versta kik da ni ook. Kzie ni in waarum dak me euh ma.. ma luut [**S**: Ja] vergeven of euh kapot goan op manier van spreken. Als ge ten al tienentander gevochten het awel ja dan blijfde gij .. ge moet vechten vind ik. *(korte pauze)*

**S** Sinds u dialyse kan u zo ne keer de..de beste dialysedag beschrijven? Nen dag da ge zegt das de meest aangename of de beste dialyse die ik al gehad heb?

**P**  *(diepe zucht)* Ja.

**S** Of is er nen dag bij u dage zegt dat is nu de slechtse dialysedag die dak al gehad heb?

**P** Awel *(korte pauze)* Da zijn de slechtste dialyse dagen da was int begin vant juur. Alskik tevoet no hiere kwam. Dak mijne dinge.. dinge last hem van euh van euh allez mijn hart en alles. Ik..ik traptege als ik van hier na beneden gingk zattek te snakken na oesem zattek en als ik binnenkwam 't smeirgens oek ze. Ze zeiden altijd ja. Ze heeft mij ten terug allez zemme mij hier een afspraak gemoakt euhh cardiologe en doar gegoan awel ja en ten hemmik nog een onder.. alle een euhmm no aalst veur een onderzoek tein hemme ze vandoar die dinge gestoken, kem doar twie doagen geblèven of drie. En kben noar huis gekommen en 't sanderduugs kommek na de dialyse. Da was allemaal redelijk goe. En kzit 's avonds ten in menne zetel en alles weurt zwet veur mijn ogen. Kem dan op mijne zeun geroepen en hij heeft derek naar den dokteur gebeld en hij zeit goat er derekt mee noar de spoed. Awelja en tein emmik hier een goei.. een week of 2 opgenomen geweest. Kem eerst in intensieve gelegen en ton laggek op een koamer .. *(zucht) (korte pauze)* of da kik da ier nog bij deun ofni of iet das na tock oek zo erg nieje ist.

**S** Algemeen verdraagt u dat goed?

**P** Joa kem er ni veel last van vinnek.

**S** En zijn er zo.. speelt er .. u.. *(korte pauze)* u ervaringen aan dialyse zelf hoe ... Wat ik hoor vooral is dat u zegt van euhm ik..ik leef graag hé en ik heb al zoveel gevochten en ik pak da er wel bij [**P**: Ja] en tis gewoon een beetjes hindernis maar tis ni.. tis ni in die mate da *(korte pauze)*

**P** Dat is .. Op langen duur wordt da een gewoonte en als ge da dan ****[09:33], awel ik zie ni in euh kbennekik content dermee bennik ja [**S:** Ja] Bennenik dan toch altijd content da.. dak nog kan blijven leven.

**S** Ja. Dus u aanvaarding voor u is hetgene dat dan.. u zegt van das hetgene da zorgt da da eigenlijk allemaal wel ...

**P** Bajaa dade als ge wilt leven moete da bijpakken moeije vinnik.

**S** En verpleegkundigen enzo, de artsen, speelt dat ook een rol hoe dade u voelt aan de dialyse vindt u?

**P** *(korte pauze)* Jah de verpleegkundigen, kmoe wel zeggen ze zijn hier heel heel vriendelijk zijn ze enn.. de dokters oek ja. Toen dakik gien dinge meer hem, hemmek euh *(lange pauze)* ze helpen u hier alster iets is moogde nog iets vragen enzo. Ik vinda..Ik vind da ze redelijk open zijn ook op da gebied.

**S** Zijn er dingen die u zelf aan de dialyse zou veranderen moest u *(lange pauze)* Moeste er iets aan kunnen veranderen, wa zou u dan veranderen?

**P** Het enigste dak zou veranderen moeste da thuis kunnen, maja dadis een kostelijk iet, ik denk da da kostelijk is of zelf moeilijk euh.. Da zou gemakkelijker zijn moede u ni meer verplaatsen, maar ja aan den andere kant komde dan toch nog nekeer buiten. Ziede nog nekeer mensen oek, anders zite kik thuis tussen mijn vier muren.. zittik.

**S** En euh u wordt gebracht naar de dialyse?

**P** Nee ik kom zelf nog.

**S** Ge komt zelf.

**P** Awel ja .. Zolang dak nog in den oto kan zitten, kannek rijje kannek euh kem daar gien problemen mee veu te rijden . Euh.. Soms een beetje moeilijk veu in te kruipen als ge ni goe zo zijt. Mo nekeer daker in zit is da geen probleem. Ik kom alle doa.. Allez elker keer met den auto kommek. En ik denk dak da ga blijven doen zolang dak kan. Ge zijt dan toch nog iet *(korte pauze)* woar da ge kunt noar uitkijken hé. [**S**: Ja] Best *(lacht)* ma wanneer ****, het is wel moeilijk van naar hier te kommen, mo bennekik content da ge dan toch nog nekeer buite zij.

**S** Ja, en moeilijk om naar hier te komen, hoe komt dat?

**P** Jamanee het dinge, euh de verplaatsing vind ik erg. Dad da een beetje dingen is, ma anders als gij thuis zijt wa doeije doar tussen vier muren kijken en na den tévé kijken. [**S**: Ja] en ne gansen dag in de zetel zitten, dadis oek geen avance hé da.

**S** En euhh de verplaatsing heeft dat te maken met het procent verkeer of gewoon de inspanning om in dienen auto en elke keer **** [**P:** Neeje neeje neeje] *(praten over elkaar).*

**P** Den tijd da ge kwijt allez dinge. Ge moe zien da ge op tijd opstoet smergens ah en voor naar hier te kommen. Want hoe eerder da ge ier zijt, hoe rapper ge naar huis kunt goan weer. Als ge loater komt en ge zijt te lang en loater aangesloten, ist den oek loater als ge thuis zijt hé.

**S** Ja. Dat aansluiten is dat iets belangrijk voor jullie? Voor de mensen van euuhmm, want natuurlijk tis zo hé als u in de is da afhankelijk van wie dat er u aansluit of wanneer u wordt aangesloten? [**P**: Ja] Is da iets zo da ge zegt van da speelt ook mee in hoe da ik mij..

**P** Awel.. Ja.. Ik ik kom graag smorgens op tijd [**S**: uhu] Dak ier rap aangesloten ben en dan smiddags da ze mij kunnen afsluiten en dak zo rap meugelijk al naar huis kan weer.

**S** Ja en das misschien allemaal hetzelfde idee van zo weinig mogelijk tijd hier uiteindelijk?

**P** *(korte pauze)* Neeje.

*(gekraak op de achtergrond)*

**S** We zullen efkes wachten.

*(gekraak op de achtergrond) [Verpleegster: Is de box al gecontroleerd?] [Verpleger: Nee sjoeke da goan ik direct noar toe. Ik ga bloed dan gaan afgeven]*

*(dialysetoestel piept)*

**S** .. Ah ja van die.. dus dat u graag terugkomt en dan..

**P** Awel ja euh alsk als ge ier toekomt en ze moeten zoveel nog aansluiten, [**S**: ja] moeije een uur wachten. Das oek ni plezant als ge hier blijft zitten me u vingeren ..zonder da ze hier verder kunnen doen.

**S** En kunde nekeer voor mij u ideale dialyse voor..beschrijven, dus das dan heel het verloop dan eigenlijk hé. Hoe ist voor u hetgeen dat beste zou gaan?

**P** Op welk euh gebied wilde euh..

**S** Dus ge.. ge beschrijft gewoon het verloop van een dialyse [**P:** uhu]vertel nekeer oe da het..da..voor..voor u het leukste zou zijn. Ge weet zoals u het zegt het vroeg aansluiten ,
[**P**: ahbajaa] beschrijf zo nekeeer de ideale dialyse dag.. beschrijven. Hoe zou da gaan?

**P** Hmmm.. Awel ja ik vinnekik aan den ene kant bennik lijk gelukkig da da bestoet, dakier na toe kan kommen, enzu euhh *(lange pauze)* ...

*[Verpleger: Sorry doe ma voort ge moet na mij ni zien zenne.. Ge moet na mij ni zien]*

*(lange pauze)*

**P** Ja.. Noa weet ik het nimeer..

**S** Awel over de ideale dialysedag bezig en u zegt dezen dag neig gelukkig maar...

**P** Awel euhh bennik gelukkig da da bestoet. Da ze u kunnen helpen. En..Anderzijds *(korte pauze)* dan is da toch zukken dingen hé da da na hier moe kommen hé als ge ne kier, allez als ge er iet kunt mee genezen of als ze u kunnen helpen dermee vinnekik.. Bennekik daar hiel tevreden over da da da bestoet.

**S** Ja. En was u vroeger iemand die veel sociale activiteiten deed? Zoals da da .. in verenigingen of vaak euh ietske gaan drinken of al die zaken?

**P** Neeje want ik em.. vroeger jaren emmik veel.. veel gewerkt. Ik hem .. Ik hem wel een schuun plosjke gad. Ik kem et van Brussel geweest . Ik hem altijd van ***** [15:58] Ik hem thuis vrie veel .. allez.. gewerkt thuis, want mijn breur die had een autocarbedrijf over. En ik hem daarin geholpen. ******[16:10] gewerkt in de garage. Ik hem mij .. aleez. veel gelegen enal veel veel weg geweest oek buiten mijn werk. Kem geweest .. Kem geweest een joar dak *** [16:24] De loatse kie een week of twee woar da we verbleven hemme oek. Dus boiten mijn werk *** [16:29] [**S**: Ja] Al dei poar doagen con.. verlof te pakken euh.. awel ik deed da allemaal graag dee ik. Ik was vrie actief int werken als ik. Kem altijd gezegd dak na Brussel ging veu veu mij uit te rusten. Ahbajaa als ge op een véloke zit, wa da moar is, kunde toch uitrusten [**S:** ja] vinnik.

**S** Maar de jaren voor da je aan dialyse gekomen bent, was da eigenlijk, was u eigenlijk al thuis dan hé?

**P** Ja ja ja ja.. Ik ben van in *(lange pauze)* van in '98. Dak nimere werk. En kem just da dingen mainnneuuhh verlof allez mijn dingen mijn ziekteverlof zolang dak dinge hem en dan emme ze mij gepensioneerd hé.. Heel..heel sereen ahja awel bennik al thuis van in.. Ik geloof dadet 2002 is, dak gepensioneerd geweest ben.

**S** Das al een tijdje hé?

**P** Ja.. Ahweljaaa.. Nu zitkik dan thuis, want het enigste dak nu doen, ben al een vijftien jaar in de vissersclub.. voe nekeer te goan vissen.. ahbajaa dadeeuhh .. ma veur de rest..

**S** Maar da kan u nog altijd blijven verder doen?

**P** Awel zolang daget goet. Want opt laatste hiere gingket ni, verleje verleje joar ginkt da ni goe ni mie. Alsek ver moest stoen, want de **** [17:54] is redelijk gruut dus alsge giel aan den achterkant doar zitten.. Das toch .. Allez dan euhh.. Da was moeilijk wast.

**S** Ja.. En nu gaat het dan terug beter eigenlijk?

**P** Baja da goet [**S**: ja] Da goet mor kzou moeten kunnen een beetje movement doen nu. Want kem redelijk *** [18:13] opt werk [**S:** Ja?] Ja alsk van beneje na hier kom hé als ik hier boven kom he ben ik ben euh van oasem bennik. Ben content altijd dak ier ben.

**S** Maar ge hebt ni den indruk da da op de dialyse allemaal achtergaat van zolang in uwen stoel te zitten?

**P** Neeje neeje neeje neeje..

**S** En u had gezegd dat u voor dialyse eigenlijk relatief weinig last had en meer kort van adem zijn [**P:** baja] waarschijnlijk ni van, van na dialyse dus ge kunt u nog herinneren opt moment da ze u onmiddelijk hebben doen starten me dialyse en hoe da de u toen voelde [**P:** Ja] en dan een tweetal weken nadien bijvoorbeeld. Voelde u zich ook effectief beter, merkte u da da die afvalstoffen .. da die weg waren of da het vocht weg was?

**P** Ni kem doe ni specioel veel veranderingen uit te halen emmek.

**S** Ge waard er op zich op tijd bij?

**P** Ik denk het ja. Ik kan ni zeggen dak van vandaag op morgen beter ben .. terwijl dak mij hiel anders gevoeld hem, kvoel mij wel anders tegen over een poar moand duur alsek die problemen van kortademig en al wa da doer bijhoorde en euhh zemme dan ne stent gestoken en da ging tein nog ni tegoed awelja tein hemme ze dan toch en euuh afspraak gevroagd bij en ben er geweest en ze ...

**S** Wie is dokter Hermans?

**P** De Cardiologe, en zeeft daar haren apprell op mijn dinge op mijn allez zeeft mij ne pacemaker gezet oek derop gezet oek. Dus zeeft da wel een beetje.. ze heeft mij gezegd da ze da bijgeregeld heeft en na nen dag of twie ging da veeele beter ginket.

**S** Dus voor u was die kort van adem zijn meer gelinkt aan u hart dan aan [**P**: ja ja ja ja ja] aan u nieren?

**S** Oké.. Euhmmmm

**P** Veu de rest kvoelekik ma ****** [19:50] kan na ni zeggen dak ma prima voel mor ja als ge zoveel problemen gehad hebt van over joaren al euhh.. Gelijk as nu ik deed. allez.. Alst een beetje slecht weer is, nevelachtig of regenachtig. Dan hemmik vrie veel last van mijne rugge nog. Ja *(zucht)* Das al meer dan twintig joar dak doemee doemee rond leup. Ge moet dermee leren leven zeide ze. Ma das gewoon oek, das ni meer veel aan te doen, jaa... Misschien opereren hoe of wa. Maar zemme mij gezegd zolang dak mijn bienen kan bougeren da ze mij ni opereren zeitem.

**S** Als u kijkt naar u gezondheidsproblemen. Wat is voor u het meeste, da het meeste op u kwaliteit van leven werkt?.. Is da dan de dialyse voor 3 keer per week hier zijn? Is da die rug, die voor u meer storend is?

**P** Doer emmik mier last van. [**S:** Ja] op andere dingen niet en.. moest ik, kzou daarveu tekenen moest ik deu kunnen leven .. zonder last te hemme [**S:** ja] Gelijk as in de zomer voelik mij gelukkig as de zonne goe schijnt en ik zit in de vijver veu te vissen en de zon schijnt op mijne rug. Da doet eeeerlijk.

**S** Stel nu we zijn in de magische wereld en we kunnen zo 1 van u gezondheidsproblemen wegnemen voor.. wa zoude dan kiezen?

**P** Ik zou mier kiezen da ze mijn rugpijn wegpakken [**S:** dan de dialyse] as de dialyse [**S:** ja] echt..k..k .. Ik hem nu al allez veu te .. veur dingen.. Het enige o.. onhandige daje het da ge a moe verploisjen. Da ge ier drie keer nen halven dag verliest bij manier van spreke. Maar aan den andere kant bennekik content bennik. Zo kommekik is buiten, nekeer iets anders.

**S** Hebde veel contact met de ...

**P** Wa da mij het miesjte intereseert, kem altijd mijn krant mee ne kie in de krant kijken en euh ne sudoku en euhh de kruiswoordroedsels da doenik allemaal graag doen oek. [**S**: Ja] Aan den andere kant ba da bij de.. houdje toch een bietje a verstand bij alles oek vinnik.

**S** Voor te trainen?

**P** Awel ja dat is ..

**S** Maar eigenlijk .. u bent ook nog ni zo lang begonnen ook met de dialyse hé?

**P** Nee van eind november.

**S** Voila dus das nu een viertal maand, maar ge hebt eiggenlijk nog nooit slecht geweest aan de dialyse.. euhmm.. Of veel alarmen gehad aan de machine?

**P** Mmmhh.. Awel da vinnik een beke ambetant da als ge een bietje bougeert da..awel.da .. da..ge ondervindt da azo [**S:** ja]. Hoe minder da ge bougeert, hoe minder batacl... hoe minder laweit.

**S** Hebt u een fistel of een katheter?

**P** Watte he? Een dinge he een katheter ja. [**S:** oké] Da trekt.. Ik ben hier gekommen bennik en tsanderdaags moestik naar hier kommen.. en dan ... zat ik... ik allez.. Hoe gaak da zegge hé. Ik had juist nen dag of twee op voorhand eennnnn..... *(korte pauze)* allez... [**S**: een echo?] een eekho laten doen van mijn armen. *(korte pauze)* Eneeuhhh.. Das er.. da gingk er ne keer afspreken voor da voor da dingen te zetten. Maar euh zebben hunnen tijd ni gehad, zebben een nieuw dingen gezet. Dat hemmen ze hier gedaan he en direct aan de dialyse gezet, als ik hier op visite moest komen.'S anderdaags is da tein gedoen.

**S** Maar dan al verschillende keren op controle geweest voor da ge aan dialyse moest?

**P** Jajaja

**S** Is da nu al wa uitgelegd wat da de nieren moeten doen?

**P** Awel ik kwam de leste euhh dingen, de leste tijd al om de zes weken. Dak op controle kwam. [**S:** ja] En dan benk terug als ze kwam, ja *(zucht)* ik was ekik oek verwonderd da ze mij zei dak derect aan de dialyse moest, da mijnen uitslag ni goeden was.

**S** Maar had u het verwacht dat het nog.., dat het iets was dat dan u te wachten stond? Hadden ze u een beetje voorbereid?

**P** Ja, ja ze had mij daar op voorbereid had ze. Want ik hem ne vriend, allez ne kameraad tis na allez just, en kgoan me den diene regelmoetig is na den control. En dienen, dat is oek ne patiënt van dokter. En dienen heeft zijnen fistel al mier als een joar he. Héy. Ik dachtekik ja me da kik oek hier kwam, we spreken wij medenen der over spreken wen. Datten hij zou rapper oan de dialyse gehangen hemmen alsekik. Khad da nooit ni verwacht en hij oek ni da kik er ierder oan geweest zen alsem em.

**S** En is dat zoiets zo da u dan.. U kent die dan ook met dezelfde gelijkaardige problemen te maken heeft en daaar dan over babbelt of is dat iets da meer ..?

**P** Nnneeee, allez allez ja, allez dakik naar hier kom en euuhh **patiënt spreekt in zwaar dialect** [24:14] en zu allez ja *(lange pauze)* ik..ik was altijd verwonderd dak derect moest binnen kommen, allez moest kommen voor allez aan de dingen, dialyse te kommen. Khad da nooit ni verwacht.

**S** Maar ge hebt geen...

**P** Ma kem daar nooit geen last van gehad van mijn nieren.

**S** Ahja zo. Euhm het is dus ook ni da u zegt van dat u in het begin moeilijk had om dat te accepteren, of da je zegt van "Oh ik heb zo in de put gezeten, of..?"

**P** Nee da da hemmik ni gad deveur.. Dadem ik ni gad, ik was meer ongerust veu, veu, veu allez veu mijn hart als veu, als veu de dialyse [**S:** nja] Aan den ene kant bennik gelukkig da da bestoat dakik hier na toe kan kommen. *(korte pauze)* Aweljaa had da hier ni bestoen kat misschien al ni mie geleefd tis ni...

*(lange pauze)*

**S** U zegt verschillende keer zo aan de ene kant ben ik blij dat da bestaat [**P**: ja] maar het enige zo van.. voor..da..da tot nu toe het enige wat je verteld is dat da vooral die tijd is hé. Dat u stoort? Is daar zo [**P:** pff ja zoo.. aah bjajaaa] *(praten over elkaar)* da je zegt of is dat euh ..?

**P** Neen ik euh.. ah jaa. Ik vin al ge na hier moe kommen da da verloren tijd is op manier van spreken, maar voor de rest ben kik hiel gelukkig dak naar hier kan kommen.

**S** U bent vooral blij dat u leven.. [**P:** dak mijn leven kan verbeteren] ja voila. Dat u.. Da u kunt blijven [**P:** awel jajajajajaja] Voor u is een machine dan iets.. Is dat nu een stuk van u... *(korte pauze)* van u leven geworden? Echt?

**P** *(korte pauze)* Njaaa.. Awel ik aanvaard dade en aan aan den ene kant omdak content ben. Ik leef nog graag t'leven.

**S** En plast u nog?

**P** Ja

**S** Ja

**P** En redelijk goe nog

**S** Ja *(korte pauze)* [**P**: ja] Dieet, oh sorry zeg maar.

**P** Ja... Euuhhh.. Ja want da da doen ik nog regelmoetig plassen doen ik. Da doen ze mij ook regelmatig vragen ze. Mo ik..em..wel doagen als ik zelf van de dialyse kom, als ik thuis kom. Dak nog ma 5 minuten thuis ben, da ik al moe goan plassen. Zo dus... [**S:** Ja] Awel ja kemmik.... *(korte pauze)* kem oek .. een ******* [26:25] en kem toch nog ni gerealiseerd da dak minder plas. Als wanneer dak hier ben, alsk vroeger deed.

**S** Ja en u bent diabeet zegt u hé? [**P:** Ja] Dus moest u dan al een dieet volgen voor u suikerziekte?

**P** Awel ja gene suiker.. Ik pak hiel weinig suiker, mor op mijn aardappelen alsk ze kook, doen ik er toch een klein beke op. Ni te vele, mo toch iet. [**S:** ja] Want als ge aardappelen zou eten da gezouten zijn, dat isssss.. Ik vind da da zo flashj is, da moeik ni hemmen. Op frieten of iet daar doek geen zout op kom, da pakkik nooit gezouten. Dat is al jaaaareen..

**S** Want ja tuurlijk u...u suikerziekte hebt u ook al jarreenn waarschijnlijk? [**P:** ja] Was u strikt in u dieet van u suikerziekte?

**P** Neeje, ik kem wel altijd per**** [27:12] suiker eet ik toch.. Allez emmik toch liever in mijne koffie. Kem da nooit ni gedoen. Ma kvermijd wel de suiker. [**S:** ja] En awelja gisteren hemmik nu nekeer euh dingen.. gezondigt ik hem een stuk taart geten. Ma ik eet ni veel taarten, ni veel zoetigheid.

**S** Eneuhh..Terug als u aan dialyse komt, komt er al, komen er soms extra dieetmaatregelen bij. Moet u op iets letten extra of niet voorlopig?

**P** Neeje, want al van wanneer dak hier de... allez de de laatste keer in de kliniek gelegen hem, hebben ze mij verminderd van euuhh... *(lange pauze)* allez ik moest 26 eenheden prikken en nu moet ik er nog 13 nog prikken moet ik. En ik sta redelijk goed op de dingen van van.. van suiker. Ik begrijp da niet, omdak dat derveure altijd 26 en dak dezelfde medicoesie al pakte, ma da ze mij verminderd hemmen en nu sta mijne suiker beter as vroeger.

**S** Bent u vermagerd ofzo?

**P** Neeje, want noe mijn dingen, noe mijn euhh kanker ben ik serieus verdikt. Dr *(cfr. Dienst "Oncologie")* zei altijd dak moest zien dak eet... allez goed eten altijd. Ik hem al geweest dak me momenten dak mij forceerde want ik was vrie neig vermagerd was ik. En euuuhh ja nu bennik *(korte pauze)* ben noa van wannier ik aan de dialyse ben toch al 3 - 4 kilo verm..afgevallen bennik toch. En kzou noa toch geire nog een beetje afvallen, want *(lacht)* kheb problemen me mijn kleren hemmik jong. Kan in al mijn kleren ni meer in.

**S** Door te verzwaren van u...

**P** Door te verzwaren jajaja. Want ik heb, ben op mijn hoogtepunt. Kem, kzen nog nooit zo dik ni geweest tot datte. Kem wel altijd 100 kilo en iets over de 100 gewogen mor,... als ik hier toegekommen ben, woog ik er 120. Twas ter in hé.. En nu nog 116.

**S** En door een beetje te veel eten of euhh..?

**P** Awel ik eet ni mir. Das zoveel als dade kem 3 sneejen brood, boter- allez brood geten van den morgen d. [**S:** Ja] En nu als ik thuis kom, dan ist nu dingen, thuis hemmik gewoon allez gewoon bruin brood. Want dingen, en dat is nen dobbelen boterham. Want das al jaren dak da altijd zo eet. Mor ik..Ja.. Ik weet ni hoe dat komt dakik zo verdikt was. Van die, me me mijn dingen me mijn kanker. Ja, en twas nogtans altijd hetzelfde dak eette euh, en de manier van eten allez ja.. Tenigste dak niejen doen dat is zout pakken, da doen ik hiel weinig nog, suiker da pak ik toch oek ni peisk.

**S** Ja mensen aan dialyse mogen gewoon ook normaal geen zout eten, maar voor u was dat al iets dat op voorhand al in u leven was.

**P** Ja ma kem nooit ni veel zout geten hemmik.

**S** Dus dat was ook geen grote aanpassing voor u?

**P** Neeje neeje neeje ni veel last van hemmik. [**S:** Ja] Grooohhh.. Het moe zijn da ik soms geen pilleke euhh allez binnen eet of chocolade stukjes chocolat. Veel veel, hiel weinig. Hiel weinig. Kan ik is sporadisch een stukske van witte eten,nemen kannik. Ma das, da val ni veel voor.

**S** *(korte pauze)* Oké... Ben aant denken... Denk dat wij eigenlijk het meeste wel besproken hebben hé? [**P:** ja] Zijn er dingen waar u nog aan denkt? Die u nog wilt zeggen, opmerkingen,..?

**P** *(korte pauze)* Nee niks speciaal. Allez aan ene kant bennekik content dat er euuhh dat.. dat.. een verbeteringen is in u...in u levensstijl eigenlijk. Want alsegij als u nieren ni meer marcheren.

**S** Wilt u dan zeggen een verbetering in u levensstijl of een maneir om u leven te..

**P** Te verlengen *(*spreken samen*)*

**S** ..-ouden ja [**P:** ja ja ja ja] Tis ni da u leven beter is sinds de dialyse?

**P** Baanieje nieje nieje nieje aan den ene kant moeije wel aanvaarden dat da euhhh.. daje ni goede zijt. [**S:** Ja] Awel jaaa.... Der kan niemand niet oandoen. Maar aan den andere kant bennik gelukkig da die bestoet.

**S** Ja ge zijt gelukkig dat ge het alternatief hebt?

**P** Ja ja ja ja

**S** Oké. Hopelijk waren de vragen niet te moeilijk hé?

**P** Neeje, *****[31:10] *(lachen samen)*

**S** Oké als..dus ik zal dan nadien nog iets laten weten, moesten we alles al samen gelegd hebben enzo. [**P:** Aweljamaja] Als u zo geïnteresseerd bent. [**P:** Allez in orde] Euhmmm merci voor uw tijd.

**P** Das toch verloren tijd he dees..

**S** Kep misschien minder verloren tijd gegeven hé

*(lachen samen)*

**S** Kem u zo al bezig gehouden. [**P:** ja] Ik ga u met uw sudoku's laten verder doen hé.

**P** Awel jamaja kem tijd ze hemmik. [**S:** lacht] En hemmik nu ni gedaan dan doen ik thuis voeisj.

**S** Volgende keer verder. [**P**: ja] Sgoed, ik gaat hier stoppen.

Interview Patiënt 3:

S Hier leggen. Ik zal dat hier zo op de .. Ma ge moogt uwen arm der zo wa hier opleggen ze. Da zal wel ni vallen hé? (korte stilte) En ik ga mijn kaartje hier pakken zodanig da ik kan noteren hé. Dus het gaat een beetje over de dialyse, maar ik zou eerst een keer willen weten hoe dat u aan dialyse gekomen bent. Wat was het probleem met uw nieren? En..

P Ja

S Vertel maar

P Ahja Ik had *poblemen* aan de nieren hé.

S Uhu

P En den langen duur emmik toch moeten dialyse doen.

S Ja en weet u wat het probleem precies was?

P Ah ik *ad* altijd last enn… (stilte)

S Had u daar last van? Ge voelde da.

P Ja ja zo dak ma wa.. alles. Ik kon mijn water moeilijk maken.

S Het komt er moeilijk uit.

P Ja

S Ja en wanneer zijte dan gestart?

P Van in.. een jaar..

S Nu een jaar?

P Ja

S En op voorhand waarde dan al bij ne nefroloog geweest?

P Altijd regelmatig bij dokter.

S Ja en ge kende die al lang dan?

P Jaja

S Hoelang al ongeveer?

P Toch al *langk* zeh.

S Ja. Dus ge had het een beetje zien aankomen?

P Ja. Zij had het zien aankomen hé.

S En ze hadden het u gezegd?

P Ja

S Ja en hebben zij dan voor een fistel gekozen of voor een katheter?

P Hier, hier ist. *Twie* ma da *gingk* ni.

S Ja?

P Da ging ni. [S: uhu] En hier *emmik nen* dingen in ma hij *goe* ni *mier*.

S Ne fistel die niet meer werkt.

P En hier *hemme* ze ook enen.

S Ja maar die zal ook ni werken?

P *Toet* hij werkt *mor* ze steken ni meer omdat teveel *bloeijen.* En nu *hemmen* ze hier hé.

S In u lies? [P: Ja] En hoe voelt .. Hoe vinde.. *da* van?

P Goed goed.

S *Zijer* content van?

P ja *kben* er *neig* content van.

S Hoelang *hebde* die al?

P Ah toch al een goeie moment zeh.

S En ge kunt er alles mee doen thuis?

P Jj.. Ik hem iemand voor ons werk te doen hé? [S: Ja] Twee keer op de week.

S Ma ge kunt rondlopen enzo?

P Joet, ik doen een beetje me klein dingen en alles da [S: Ja] gaat.

S En hoe be..Hoe Hoe ist voor u aan dialyse?

P Als ik weerkom een beetje moeg mo als ik rust ist ewa beter.

S En ist.. Moede veel rusten of euh..?

P Neen da..een beetje snoens rusten we altijd hé.

S Ja en tegen ’s avonds zijte er al terug door dan?

P Ja jaa. [S: Ja] Ik maak men eten enn..

S Ja en hebde last.. aan de dialyse zelf? [P: Neennn] Ge voelt niks?

P Ja niks.

S Niks. Euhm.. Het onderzoek gaat een beetje over of.. hoe da.. hoe.. over “adequacy of dialysis” hé? Dus das de..de..de. Maar das een Engelse term dus ik weet niet of da u ie.. u iets zegt? (korte stilte) Als get vertaald letterlijk dan is dat adequaatheid van u dialyse, maar ik weet ook ni of da.. Das ook nog moei.. moeilijke zin eigenlijk hé? Euhm en als het dan probeert zo wa in mensentaal te zeggen..Euhmm.. Is da een goeie dialyse. Dus de vraag is een beetje “Wa is een goeie dialyse voor u”?

P (lange stilte) Goed hé? (aarzelend)

S Awel ma WA maakt een dialyse da da de dialyse goed is? Zijn er dingen die belangrijk voor zijn voor u aan de dialyse?

P Neen

S Niets? (lange pauze) Kunde u dingen voorstellen waardoor da u dialyse slecht zou zijn?

P Ik vind da da goed is.

S Ge vindt da da goed is? Maar zijn er dingen dat het slechter zouden maken?

P Neennn.

S Neh. (korte pauze) Niets .. Totaal (P: Neh) niets?

P Nee

S Neh. Hebde het ooit lastig gehad met de dialyse?

P Hier just met de katheter ze.

S Ja. Maar dialyse zelf? [P: neen !] En je hebt ook nooit last gehad om … te beginnen met dialyse? [P: Neen] Ge hebt ook nooit daar verdriet van gehad?

P Nee nooit niet.

S Nee, ge hebt dat gewoon aanvaard?

P Ja

S Ja en ge verdraagt het goed?

P Jajaa.. Ik verdraag het heel goed.

S En zijn er dingen die je zegt van “Da vind ik nu jammer aan dialyse bijvoorbeeld?”

P Ja ge moet, moeijje hmm?

S Ja. Awel ma wa.. Kunde zo wa u gevoel over de dialyse, wa je er allemaal van vindt nekeer beschrijven?

P Ik vind het goed.

S Ja? En waarom vinde het goed?

P Ha, da da ma (lange pauze) Ik vind da ak da doen [S: Ja] bennik ewa beter.

S Ge voelt u beter na [P: Ja] dialyse. En en hoe merkte da? Hoe voelde u voor de dialyse bijvoorbeeld?

P Ik was altijd moeg en alles.

S Ja, dus eigenlijk zijde minder moe.. [P: Ja] met de dialyse?

P Ja.

S Ja. En ge merkt da verschil?

P Ja.

S Oké, das een belangrijke hé?

P Ja.

S Ja. Andere zaken? (korte stilte) Da je merkt met de dialyse, da je zegt van die dialyse werkt, da marcheert?

(Lange stilte)

S Zijn er nog dingen dade zegt van.. Zijn er nog zaken dade zegt van die dialyse werkt, ik voel da?

P Ik.. (zucht) Ik vind da da goed is.

S Ja.. En .. Ik ga nekeer kijken. Moede gij vocht af? Gij moet vocht af ook hé?

P Jaaa.

S Merkt ge da bijvoorbeeld?

P ja van tijd aan mijn benen. Da zwelt. [S: Uhu] Ma nu zijn ze zo ni mie dik.

S En hebde in het begin van de dialyse zo … (korte stilte) veel alarmen gehad of veel moeten komen extra of …?

P Neen.

S Ge hebt daar allemaal geen dingen van gehad. En hebde da gemakkelijk ? Woonde gij thuis alleen.. of hebde [P: Neeneeen] gij ne man?

P Mijne .. Mijne man.

S Me ne man.

P Ja.

S En hebde kinders?

P Een maske.

S Een meiske ja. En hoe oud is die dan?

P 43 ma ze woont hier in X hé.

S En heeft zij kinderen?

P Nee.

S Ze..Ze is alleenst.. -aande

P Ja.

S ja. En euhmm als ge aan de dialyse kwam hé? Moest.. Haddet gevoel da da voor u familie ook een aanpassing was dan?

P Ja.

S Ja. En kunde daar wa over vertellen?

P Ah, maja ze vonden da goed hé. Dak da deed voor mijn gezondheid hé.

S Ja. Maar het alternatief . Wat de andere opties zo een slechte optie was waarschijnlijk. [P: Ja] Ja, hebde ooit gedacht om niet te starten?

P Da niet. Ik was content dak startten.

S Omdat ge u zo slecht voelde?

P Ja.

S Ja, en hadde verwacht da ge u beter ging voelen?

P Jaa…

S Ja, oké. En lukt het met alle aanpassingen dade moet doen?

P Ja. Voor te eten enn.. wat dak drink en alles.

S Ja, da lukt?

P Joet da lukt. (korte pauze)

S En is da lastig?

P Nieennt dadis ewa moeilijk maja. Voor mijn eten hé. [S: ja] En fruit moenik ook oppassen hé.

S Ja. En hoe doede da met het eten thuis? Want uwen man mag waarschijnlijk wel vanalles eten of..?

P Ja jaaa.

S Doet hij mee?

P Ja hij doe mee. Hij moet oek een beetje régime doen hé.

S Ja. Gij kookt zelf nog?

P Jaja.

S Ja. En is da.. (korte pauze) Hebde veel tijd nodig gehad om da zowa onders de vingers te krijgen om te zien wadade wel en ni moest doen of euhm.. ?

P Jaaaa.. Da gaat.

S Ja da gaat. Als u zo nekeer kijkt dus ge zijt bij dr geweest en die zei van: “Ja u nieren gaan achteruit, gaan achteruit” en gij voelde u slechter worden en dan zegt ze “Kijk ge moet aan dialyse” [P: Ja] hé? En ge voelt u dan wel beter, maar ik kan mij voorstellen dade ook dingen hebt moeten laten of u leven hebt moeten herschikken. Kunde daar wa over vertellen wa da da….

(lange stilte)

P Maja..

S Ma ik weet ge hebt daar ni veel keus, da weet ik, maar hebde.. Wete nog wa ge allemaal moete.. Euhmmm.. Laten hebt?

P Ja. Al da rrr..rauw is. Mag [S: Ja] ik ni eten [S: Ja] Frr… Euh just nen appel en een een peer. Da [S: Ja] mag ik eten. En soep moenik ook laten.

S Ja. En das op het eten hé?

P Ja.

S En hebde op andere vlakken zo het gevoel dade dingen hebt moeten laten?

P Jama da gaat.

S En kunde wa vertellen wa dade moeten laten en.. [P: Ahja] Hoe ga je er mee om?

P (zucht) Allez frr.. ll.. groenten enn.. Dak allemoel moe koken.

S Ja… Ma ik wil zeggen dus niet van eten hé. Bbijvoorbeeld euhmm dingen me u familie doen, of andere zaken die me dialyse gepaard gaan: naar hier komen enzo… Hebde daar moeten zo nen tijd uwen draai in vinden of ge hebt daar nooit last van gehad?

P Nooit.

S Neh. Ge hebt het makkelijk aanvaard?

P Ja.

S Ja. Oké. Kende andere mensen, behalve hier in de dialyse, die ook dialyse doen?

P Neenn, wemmennn… Niemand ni da we weten.

S Neh. Dus ge wil.. En wiste op voorhand waar da ge aan begon dan?

P Ja.

S En hoe kommet? Hebde een keer (korte stilte) Hebben ze het u goed uitgelegd? Of zijde nekeer komen kijken of..?

P Zebben het goed uitgelegd en..

S Ja. (korte stilte) Der is niks voor u da onverwacht was of dat een teleurstelling was of [P: Neen] juist goed meeviel? (korte stilte) Neh. En hoe komde naar hier?

P Met vervoer van Ambulancedienst

S Ja, content van?

P Ja, ben der neig content van.

S Wanneer moete zo vertrekken?

P Om..euh.. Om 6 uur en een kwart.

S En wanneer zijde thuis?

P Kwart voor ne… één.

S Kwart voor één. En lutk dat een beetje? [P: Joet] Want.. Want das nen halven dag ma..?

P Da lukt. [S: Ja] Da lukt.

S En dan komde thuis zet ge u eten klaar?

P Ik maak ’s avonds eten.

S Ah ge maakt ’s avonds eten. Uhumm. En doet uwen man dan ietske, zo probeert u wa te helpen?

P Jaja jaja. Hij helpt mij. Hij doet de comisskes hé. [S: Ja] Hij helpt ma een beetjen hé.

S Ma tegen ’s avonds zijder al door?

P Joa da goet voor op maan gemak.

S Ja. (korte stilte) oké.

(lange stilte)

S Ik denk da ik het meeste al gevraagd hebben hé?

P Voilà.

S Tgaat een stukske sneller als bij u [P: En ik ben neig content ---] buurvrouw. [P: Neig content en alles veu .. veu al dei da ier liggen.]

S Zijt ge content van de mensen rondom u?

P Ja. We zijn hier in compagnie en..

S En houdt ge u zo een beetje bezig dan?... Houdt ge u zo op die manier bezig? Zo wa babbelen en.. ?

P Joa da doen ik.

S Ja. En stel nu plots dade naar een andere groep zou moeten? Zou je da lastig hebben?

P Neen.

S Ge zou u kunnen aanpassen?

P Joa.

S Ge.. Ge zijt zo iemand die zo.. Dingen aanvaard waarschijnlijk?

P Ja ja..

S Niet teveel … Niet teveel proberen piekeren.

P Nieje.

S Neh. Oké. Goed. Dan ga kik euh.. al stoppen met de opname. En euhmm… Voilà. Gewoon zien dat alles uit is hé. Zo (korte piep) Zijn op zich geen moeilijke vragen, maar iedereen heeft een ander antwoor… [Opname stopt]

Interview Patiënt 4:

**S**  De bedoeling is ook da achteraf als er dan euh.. resultaten zijn van het onderzoek, dat als ge geïnteresseerd zijt, dat je het ook. Da we die ook aan jullie gaan euh.. vertellen hé. Goe? Euhm ik zou anders gewoon vragen om of u eerst een keer kan vertellen hoe dat u aan dialyse terecht gekomen bent? Dus wat het probleem was met uw nieren enzo euh?

**P** Hoe da ik daar eigenlijk bij gekommen ben?

**S** Jah.

**P** Vermoeidheid, dat ik dus.. (korte stilte) een bergsken ni meer op kon en dan na algemeen onderzoek.

**S** Uhu.

**P** En..

**S** Zo zijt ge der op uitgekomen? Euh.. Wete wat de oorzaak was?

**P** Ja..

**S** Hoelang wast geleden?

**P** Ik ben hier euhhh.. van innn..novemmberrr… (lange stilte) veertien.

**S** Ah oké. En altijd hier geweest?

**P** Awel ja, a..awel ierst met die onderzoeken en der achter beslist..

**S** Ja.

**P** Van naar hier te komen.

**S** Oké en hoe vergaat u dialyse?

**P** Hmm?

**S** En hoe ist dialyse?

**P** Ben content, ben overal content.

**S** (lacht) Zijde overal content?

**P** Ook over het eten.

**S** Hebde int begin u moeten aanpassen, weet ge da nog? Hoe dat da in ’t begin was?

**P** Neenk.

**S** Tis a-

**P** Ik heb geen pijn hé, ik heb ne katheter hé.

**S** Uhu. Zijjer content van?

**P** Wel zebben al ne keer veranderd.

**S** Ja? Een nieuwe gestoken wilde zeggen? Ja.

**P** Nen anderen. Ierst is die weg en ene in de lies gestoken.

**S** Uhu.

**P** Ma dan hem ik langk geweest me zo’n blauw bille.

**S** Oei.

**P** Bja. Da gaat allemaal over hé.

**S** Ma ge zijt nu al nen tijd met den dienen? Ja.

**P** Van in oktober ist den dieje.

**S** Dus dat het binnenkort een jaar gaat worden?

**P** Dieje ja.

**S** Negen maand nu zoietske? Ja. ’T onderzoek zelf gaat hem eigenlijk, dus we willen weten wat dat patiënten denken van medische dingen, ja? Dus willen ze aan patiënten van..die dialyse hebben, vragen hoe dat de dialyse.. Allez wat dat dat allemaal inhoudt voor hen. En de vraag is een beetje, maar ik weet niet. Kan u Engels? Neh. Dus ik ga eerst een Engelse term zeggen…

**P** Kwas gebuisd opt school.

**S** Voor Engels?

**P** Voor Engels.

**S** (lacht) Ge zijt er toch door geraakt.

**P** Baja da was in den tijd om van de schoolkwestie me euhh.. minister Collard
(*cfr. Minister van Openbaar Onderwijs*) Wa..Want die i..is oek nog in de stoet geweest o..om..

**S** Ah in de protesten?

**P** Ha, awel het protest hé.

**S** Goed, nu willik hem.. Kga hem gewoon zeggen in.. in’t Engels en direct vertalen dan al. (lacht) Ist goed? Dus dat is.. Tgaat een beetje over “Adequacy of dialysis” en als ge dat letterlijk vertaalt, dan is dat adequaatheid van dialyse. Maar dat is nog altijd ne moeilijken term hé. Kweet niet, zegt u dat iets?

**P** Adequaat?

**S** Ja. Adequaatheid van dialyse. Kunde u daar iets bij voorstellen?

**P** Awel ja, nee? Jamanee allez euhh.. Dak dat gewoon ben of wat?

**S** Als ge het nog in mensentaal wilt zeggen hé, euhmm dan is het goeie dialyse. Ist de meest eenvoudigste vertaling zo een beetje, dus de vraag is: “Wa maakt er voor u een goeie dialyse?” (korte stilte) Ge moogt op u gemak nadenken ze.

**P** Ja gewoen, awel ja kem kik da machien.

**S** Ja?

**P** Een.. En ge kunt er zelf ni veel aan doen hé? En ast, ast een beetje lawaai maakt.

**S** Wa wilt da voor u zeggen als da wa lawaai maakt?

**P** Awel da dat er ietss.. iets.. hapert.

**S** Ja.

**P** En dan komen ze op het knopke duwen en euh..

**S** Ja. Andere zaken da je zegt van das goed? Allez da..daaraan merk ik dat een goeie dialyse is bijvoorbeeld?

**P** Awel ja.. Das het.. Tis levenslang hé? Dussss ja.. moe..moet er mee leven leven hé en euh da doe geen pijn dus..

**S** En zijn er ding.. zaken aan de dialyse die belangrijk zijn dan voor u om te zeggen dan ist voor mij een goeie dialyse?

**P** Wa verschil is dat als ge ’s avonds weggaat tegen.. tegen als ge toekomt ge wordt dat niet gewaar hé.

**S** Nee? Merkte verschil voor en na? (korte stilte) Ge hebt geen veranderingen?

**P** Ja, tis binnenin hé.

**S** Uhu.

**P** Maar ge hebt chance-

**S** Maar sommige mensen hebben wa last als ze van dialyse weggaan bijvoorbeeld?

**P** Ahneenee dat heb ik nog niet gehad.

**S** Neh.

**P** Awel soms gaat dat bloeddruk een beetje lager.

**S** Uhu.

**P** Mo.. maar da.. da komt weer, ik recupereer.

**S** Ja.

**P** Gelijk als vandaag wast bijna veertien mijnen bloeddruk, omdak..

(lange stilte)

**S** Misselijk?

**P** Ja, joemoe als ik, als ik van huis uit al..

**S** Ja?

**P** Neem ik al ne plastiek zak mee en als ik hier toekom..

**S** En hoe komt het?

**P** Awel ja ie.. iet verkeerd geten waarschijnlijk hé.

**S** Ah das ni standaard? En uwen bloeddruk was hoger?

**P** Ja, enfin ja o een beetje nerveuzer maja.

**S** Ja, door de dialyse hebde gij uw.. Hebde gij veel dingen moeten aanpassen?

**P** Wat?

**S** Met de dialyse te starten, zijn daar.. Is u leven... Dus..da..das..das drie keer per week?

**P** Awel ja, dus ik mocht kiezen in de voormiddag of de namiddag.

**S** Ja?

**P** En ik heb in de namiddag genomen omdak langer zou sl.. en ook omdat ik thuisverpleging heb. As.. Als ze hier kommen tussen zeven en acht, dan wilt da zeggen dat de thuisverpleging moe..moe.. moe vroeger kommen hé.

**S** Uhu ja?

**P** En dus heb ik den achtermiddag genomen hé.

**S** Ja?

**P** Voor de rest..

**S** En hebde dingen die e vroeger in u le.. die de vroeger deed die de moet laten bijvoorbeeld?

**P** Awel ik trek mijne plan die andere dagen hé.

**S** Ja?

**P** En ik bedoel.. Ik heb mijnen auto moeten verkopen omda ik toch ni veel per oto ni meer reed en ze komen mij brengen en halen.

**S** Uhu.

(lange stilte)

**P** Dus.. Ja ja. zijde nu al bezig? Alle hup. En spreek ik genoeg?

**S** U spreekt genoeg denk ik ja.

**P** Kan da hier allemaal in?

**S** Ja das de GSM en das euh.. Ma diene GSM pakt het minste geluid op, me dienen andere moete gelijk luid genoeg spreken om veel euhmm.. Ma da geeft ni als er af en toe ni veel gezegd wordt zeh.

**P** Mja.

**S** Ik heb da liever dat ik u wat laat nadenken voor dak euh.. ik onderbreek ofzo.

**P** Ik denk dat ik hier nog nog hiel vroeger geweest hem me een enquête en dan heb ik gezegd tegen da meiske: “Ik zalt aflezen de vragen en dan bijvoeren.” En ondertussen gingen ze nen andere en uwen tijd was minder versleet.

**S** Ma dit is anders hé? Dit is een interview echt hé.

**P** Ja.

**S** Der zijn geen voorgemaakte vragen-

**P** Mmmaja.

**S** -der zijn geen euh.. Ze kunnen het ni zomaar invullen hé en ge kunt ook moeilijk tegen mijn GSM staan babbelen hé?

**P** Mmmhh.

**S** Als ik weg ben zo.. hé? Euhmm.. Woon.. Woont u alleen thuis? Ja. Vroeger getrouwd geweest bijvoorbeeld?

**P** Ja mor euh.. (korte stilte) Half getrouwd.

**S** Half getrouwd?

**P** Awel neenee, (zucht) dus ik ben in zestig getrouwd.

**S** Uhu.

**P** En in tweeëntachtig is ze me.. Da ding.. Kwas bij mij thuis café.

**S** Uhu.

**P** En ze is blijven plakken me nen anderen en ik.. Want ik was daarbinnen getrouwd.

**S** Ja?

**P** Dermee bennekik weggegaan naar een appartement hier in drieëntachtig, dus van zestig tot drieëntachtig.

**S** Ja.

**P** En derachter hem ik hier.. Heb ik gien vrouw ni mier aangetrokken.

**S** Nee? Das al dertig jaar.

**P** Grohja..

**S** En hebde kinders?

**P** EEN en die heeft in de echtscheidingskosturen partij genomen voor de vrouw, alhoewel dak ze laten oppakken hem voor euhmm.. overspel op een ander hé.

**S** Ja?

**P** Terwijl da ze op een ander was hé.

**S** Ja.

**P** Ja. En dermee..

**S** En hebde.. Hebde daar dan nog contact mee dan of niet echt?

**P** Emmik?

**S** Hebde daar nog contact mee met?

**P** Met hem ni.

**S** Neh.

**P** En hij me mij ook ni.

**S** En euh.. Zorgt er iemand voor u of zegde van ja met de thuisverpleegkundige?

**P** Ahja ma nee nee, zouk als ik allien ni was ik ging nog werken dus da ging doer nog goed.

**S** Ja en wa deed ge van werk toen? Wat dede van werk?

**P** Bediende in de pensioenkas van de mijnwerkers en..en das dan nu das er geen mijnwerkers ni meer zijn, is die opgedoekt. Eigenlijk werd ik gefusioneerd de eerste januari na mijne verjaardag van zestig jaar. Ik heb dus vervroegd pensioen genomen hé.

**S** Ja.

**P** En dat is ook al van in zevenenzestig geleden. Euh.. Zevenennegentig geleden.

**S** Ahja. Oké…

**P** Van zesendertig na zevenennegentig hé.

**S** Ja.

**P** Vanaf dan bennekik content.

**S** Maar ik heb u onderbroken en ge waart aan het uitleggen van euh.. Da ge in het begin nog werkte en dat dat dan allemaal ging, voor wa hulp enzo was ik aan het vragen.

**P** Ja.

**S** Dus aan het vragen voor hulp en dan zegt je ja in het begin werkte ik nog.

**P** Ja.

**S** En toen gingt da allemaal en dan heb ik u onderbroken.

(korte stilte)

**P** Werken? Ahja.

**S** Ja.

**P** Als ik gepensioneerd was?

**S** Ja, ik zei: “Wie zorgt er dan voor u?”. Gaat dat allemaal goed zelf?

**P** Awel ik.. woonde op een appartement allien.

**S** Uhu.

**P** En als ik nimier ging werken, gingkik hier in de streek nen dagschotel eten. Gelijk als nu, hemmik op in de dingen in Lokaal dienstencentrum hier in het wooncentrum.

**S** Uhu.

**P** Van het OCMW, hemmekik ook nen dagschotel hé.

**S** Ja.

**P** En aan vier euro krijge we hem.

**S** Ja.

**P** En dan hemmik mijne plan getrokken. Ik was altijd bezig me ander op ss..ss..sportgebied, secretaris van het ien en t’ander.

**S** Ja altijd veel bezig geweest? En doet ge nu nog iets extra?

**P** Hmm?

**S** Zijde nu nog actief in.. verenigingen?

**P** Ik volg nog altijd, ik ben nog altijd in het bestuur van de.. van de clu.. van de wandelclub.

**S** Uhu.

**P** En van de sportraad mor ik ga ni meer naar vergaderingen omda ik me… De vergadering is altijd gewoon op het eerste verdiep me ne smallen trap en ik kan daar.. Nieje.. no.. (mompelt) ne stoel pakik, mor als ik ver.. euh verder ga heb ik o een

**S** Ja.

**P** Oek vo dan man evenwicht te houden.

**S** Dus zelf gade ni meer gaan wandelen dan waarschijnlijk?

**P** Awel nieje.

**S** Ma wel de dingen zo de activiteiten als ge kunt euh..?

**P** Ik heb..goak me nen taxi.

**S** Ja.

**P** Ten als ik naar t’centrum voor vijf euro gaan en vijf euro were keren.

**S** Heeft X een centrum?

**P** Ja tuurlijk.

**S** Ben ni van hier.

**P** Ja mo.

**S** (lacht) Ma ge kunt.. Ge doet nog af en toe nekeer buiten komen en…?

**P** Tuurlijk.

**S** Ja en de dialyse heeft u daar niet in gehinderd?

(korte stilte)

**P** Ge moet er wa aaa..aan aanpassen hé.

**S** Ja. Oké. Ben aan het denken. Ik denk dat ik alles gevraagd heb.

**P** Ah kweeget ni.

**S** Jah, ben zo wat aan..

**P** Ik ben nog altijd positief.

**S** Ja?

**P** Ja allez, ben zondag op restaurant geweest en die lange zetel hé? Zijn er kussen om achter u zijn.

**S** Ja?

**P** En er kwam toe aag een koppel me een meiske van zeventien achttien jaar. Ik nam da kussen wa achter mij, wil je mij kussen?

**S** (lacht)

**P** Ziede ja wat dat doet me de mensen?

**S** Zo van die ja.. Ewa grapjes maken en euh.. ja.

**P** Of in den hof waren der verleden.. verleden week met de Gentse Feesten (cfr. openluchtfestival in Gent) was er dar een euh groepken. Jongen en meiske per één die euh.. zich kammen.. kwamen uitrusten na da ze weggeweest waren hé. En euh.. ik begon te spreken met één of andere en die langste kwam bij mij om te babbelen. Hij was van Tsjetsjenië (cfr. Russische autonome Kaukasus-republiek).

**S** Moh?

**P** Ja en d..die.. Hij woont hier al van int jaar vijf en toen was hij tien jaar. Dus..

**S** Tweeëntwintig.

**P** En euh.. Die meiskes euh.. spraken ook euhh van.. van.. ze zitten der opt gras. BB.. allez conversatie een bietje en op een zeker moment vroeg ik aan ien van die meiskes: “Zeg wilde neki kijken?” (lange stilte) “Naar het oor van dat ander meiske offff te zien of ge daar ni niks speciaals ziet?” (korte stilte) Ze doet dade. “Kzie kik hier niks.” zeg ze. Kzeg: “Awel das een teken da ze ni meer groen is achter haar oren.”

**S** (lacht)

**P** Kende da versl…?

**S** Jaja da zeggen we ook. En hadden ze het door?

**P** Ma ge moet toch een mopken.. Heh?

**S** Hadden ze het door?

**P** Jajajajaja, jamaja.

**S** En maakt ge hier ook zo grapkes dan zo af en toe is?

**P** Baja hé.

**S** Ja.

**P** Ben onlangs tachtig jaar geworden. Hé? Ik heb een fiest gehouden, in de feestaal van het wooncentrum.

**S** Uhu.

**P** Met een honderd man uitgenodigd, want kmocht er me ni veel meer binnenkomen.

**S** Das al de moeite hé? (lacht)

**P** Ik heb daar mijn speech, da was ni anders als zelf uitgevonden in werkelijkheid.

**S** Ja.

**P** Of ef.. echt gebeurde moppen verteld. Allemaal op papier geschreven en ge..gerepliceerd thuis.

**S** Ja.

**P** Dat heb ik verteld, maar twaren gien kinderen bij hé.

**S** En was er iemand van hier bij?

**P** Kep ze wel uitgenodigd maar euh..

**S** Van de dialyse? Ja.

**P** Als ge me ne groep zijt, ofwel moeten ze allemaal uitnodigen en..

**S** Ja.

**P** En dat is wel.. en de dokteres heeft mij gezegd best.. (korte stilte) Ziet da ge, kzal ze ni vernoemen, dat ze uitgenodigd zijn want anders ist er hier jaloezie hé.

**S** En zijn er gekomen?

**P** Hier? Van hier?

**S** Ja?

**P** Neen.

**S** Ahja. Gept ze gewoon allemaal..

**P** Jamaja twaren der al zo.. Ik had er honderdentien uitgenodigd.

**S** Ja.

**P** Om.. Omdat ik er ze..zekers op die.. op die.. honderd, want ze zeggen dan op of.. Het.. Twas int midden van de winter hé.

**S** Ja.

**P** (mompelt) Maar goed verlopen.

**S** (lacht) Ge zijt er content van zegt?

**P** En tis daar dan foto’s van genomen en fi.. en filmreportage gemaakt van foto’s. Ik heb die.. Zebben.. Die..Dieje da foo.. foto’s gemaakt heeft, heeft da op internet gezet want ik werk nog altijd met internet. En die heb ik dan doorgestuurd nor mensen die der geweest zijn en de die die ni geweest zijn.

**S** Allez ge hebt u bezigheid?

**P** Jajajaja, kep der nen tijd van in juni ben ik er mee bezig geweest. (korte stilte) Tot in december erge.. als ger aliene voor staat.

**S** Ja.

**P** En euh.. mijn nichten dus euh.. De kinderen van mijn zuster (mompelt) tzijn er toch een stuk of.. een stuk of zes.. Zes geweest. Das (hakelt over zijn woorden) sociale assistente, van op mijn werk die mijn medisch dossier ophoogt, kwestie van tussen te komen bij de socialen dienst.

**S** Ja?

**P** En na bovenop de euh.. standaard dingens.

**S** Standaard dingen? Ja uhu.

**P** Ennn die was daar ook en die had in november een kindje gekocht en ze was in december na tfiest gekommen per oto.

**S** Amai.

**P** Kem kik daar nog altijd correspondentie mee. Ze heeft mij zelf ne foto gestuurd van da kindje. (mompelt 16:26) Ja correspondentie en euhh.. Kwas vandaag begonnen mee.. voor den dokter van de professor hier nen brief te maken, hoe da ik mij voel en hoe dat ik da zou moeten doen om mijn lichaam.. (korte stilte) over te maken aan de universiteit. Van Gent. Ben nog bezig da op te maken. En kwil dien brief afslui..afsluiten en bij ne groep zetten, moenik waarschijnlijk vergeten hemmen van hem op te slaan. Dermee..

**S** Oh volledig verb.. Volledig kwijt.

**P** Jamoe das ni.. Joe da sta ni veel op zenne. Zo mor allemaal wa teksten enzo..

**S** Zijde daar zo mee bezig zo euhm wat er hier achter..?

**P** Hmm?

**S** Zijde daar mee bezig?

**P** Baja. Ahbaja als ge alliene woont. En der zou..

**S** Ja.

**P** (mompelt) Dus tmoet op, moe rekenen op anderen.

**S** Ja.

**P** Die ook ouder zijn als den andere.

**S** Ja.

**P** (mompelt) En nu me.. me die In gans groot X zeventien parochies zijn er nu nog twee paters uit de Congo hier komen missen doen en die.. en deken.

**S** Ja.

**P** Tes alles.

**S** Ja das ni veel hé.

**P** Er zijn veel kerken die..

**S** Ja.

**P** Gesloten worden. Ahja (mompelt) Zo benk mijn tijd.. Mijn bezigheid.

**S** Zo passeert alles sneller hé ook? Hebde dan iets tijdens dialyse soms zoiets van voorbereiden of rusten vooral? (praat luider) Tijdens de dialyse of da je vooral rust of dan ook af en toe iets..?

**P** Awel nu hemmik mijne rust anders kijk ik na tv.

**S** Ma zelf ge gaat ni uwen computer meepakken da ni?

**P** Nee, hier naast mij ist er één die dat doet, mo..

**S** Das dan te modern zeg? Oké, merci ik heb alles dat ik wou vragen is zeker gevraagd.

**P** Allez da weet ik ni hé?

**S** Jaweell..

**P** Gept mij-

-OPNAME STOPT-

Interview Patiënt 5:

**S**  Dus euhm ... anders even nog kort uitleggen hé

**P** Ja

**S** Dus de bedoeling is eigenlijk dat we mensen ondervragen, patiënten ondervragen *nekeer* te luisteren naar die ervaringen rondom de dialyse. Om dat achteraf goed te kunnen bekijken en te analyseren nemen wij alles op ..op een bandrecordertje.

**P** Ja

**S** Nu enkel ik kan daar aan en de andere onderzoekers en nadien wordt dat gewist. Is dat een probleem voor u dat da euh..?

**P** *Neeje*

**S** Tijdens het onderzoek *zalk* ook af en toe .. euh interview *wa* opschrijven of dus als u iets vertelt en denkt aahja daar wil ik straks nog *wa* meer over weten, *da* ik u niet moet onderbreken he.

**P** Ja

**S** Goed ? ... *Kzou* zeggen euhm begin maar iets te vertellen over uzelf en ook over de euh .. de nierproblemen en hoe u aan de dialyse gekomen bent, een beetje inleiding.

**P** Ja. Euhhh ik heb een nier uitgepakt he euhh ze zagen allemaal cysten.

**S** Mmmhhmm

**P** Ennneeuhh *d´andere hebbek* nog maar die is ook eigenlijk ..... En ze hebben er dan een dialyseverpleegster bijgehaald euh thuisdialyse

**P** En da ging ni meer weg en dan hebben ze mij dan eigenlijk gewone dialyse ..... das ongeveer een jaar euhhmm da ik aan de dialyse ben.

**S** Uhuu. Die thuisdialyse volgde u dan zelf ook buikspoelingen?

**P** Buikspoelingen.

**S** Ja. Ni geen.. euh hemodialyse met een toestel ?

**P** Neenee

**S** De buikspoelingen.

**P** Buikspoelingen ja .

**S** En dat was overdag lege buik of ... een volle buik ? En de rest voor ‘s nachts?

**P** *Da* was een volle buik dialyse ja ja.

**S** Oké en hoe hoe vergaat dan u dialyse?

**P** Awel ik *goan* heel vermoeiend.

**S** En kunt u een voorbeeld geven van dat dat vermoeiend zijn?

**P** Awel he als ik thuis kom na de dialyse en dan kan ik niks meer doen.

**S** Ja en u merkt dan verschil als u da vergelijkt met die peritoneaal dialyse met die buikspoelingen?

**P** Ja

**S** Ja oke . Het onderzoek dat wij euhhmm op voorbouwen, hoe dat wij ons idee gekregen hebben, wat we bij de patiënten peilen achter ervaringen euhm komt eigenlijk voort van een Engelstalig onderzoek en daar euhmmm hebben ze wa onderzocht `adequacy on dialysis´ he. Das een Engelse term, als ik het goed vertaal, *ge* kunt het eigenlijk niet goed vertalen, als we het letterlijk vertalen komt het eigenlijk neer op de adequaatheid van dialyse.

**P** Ja.

**S** Zegt dat u iets? Of *kunde* u daar iets bij voorstellen?

**P** Mmhh.. nee feitelijk ni nee..

**S** Euhm als ik dan zou zeggen een goeie dialyse. *Wa zoude*.. Wat omhelst dat voor u? *Wa* maakt dat een dialyse een goeie is of *wa* zijn goeie dialyses?

**P** Njah. (korte pauze) Ik vind die hier wel beter dan euh dan peritoneale.

**S** Ja ? De hemodialyse vindt u beter dan ..

**P** Ja

**S** Enn..

**P** In het begin *ni*, maar naar het einde wel .

**S** Ja . Begin is van in het begin van de eerste behandeling of telt het begin van de behandeling?

**P** Het begin van de behandeling.

**S** Maar hebben we het dan over de eerste keer dat u geweest bent?

**P** Ja.

**S** Ja , de evolutie in het eerste uur van dialyse is altijd slecht en dan beter?

**P** Neeje.

**S/P** Nee [ samen].

**S** Nee. Bij de opstart wilt u zeggen?

**P** Ja.

**S** En in welke mate vindt u dat dan .. Merkt u dat dat beter is dan de .. (klikt met tong)

**P** Awel in het begin van de dialyse voor altijd toch nog een uurtje.

**S** Uhuumm .

**P** Euhmm en daarna naar hoe dat het vordert euhh verbetert da wel.

**S** En die bloeddruk.. aan de bloeddruk merkt u dan dat het beter is?

**P** Ja ja.

**S** Zijn er ook andere dingen dat u voelt, dan u voelt dan dat het beter is?

**P** (lange pauze) Neeee..neee (twijfelachtig) Nee *ni* speciaal.

**S** En u zei dat euh dat dat de dialyse nu beter dan de buikspoelingen [**P:** Ja]
Hoe merkt u dat dan?

**P** Euhh aan mijn benen enzo die zijn ni meer gezwollen.

**S** Uhuumm

**S** Ja. Uhu andere zaken?

(korte pauze)

**S** Andere verschillen?

**P** Neeee.. da heb ik zo ni..

**S** Nu een goeie dialyse, [ja] stel da u ..euhmm.. beschrijf *ne keer* het.. het de dialyse zo.. vanaf het begin tot het einde. Dus vanaf opstaan en hoe *daje* hier komt , kan u mij de perfecte dialyse .. vertellen?

**P** NJah.. Das wel moeilijk ze.. Euhh s´morgens voel ik mij altijd beter als ik opsta [**S:** Uhu]
Tis naar mate dat het middag wordt, voel ik mij euh moe.. [**S:** Uhu]
(gemompel) ja (korte pauze)

**S** Is dat de dagen van de dialyse of de dagen van de niet-dialyse?

**P** Dat is *iederen* dag

**S** Elke dag, want u start dus het beste?

**P** Ja.

**S** En dan komen ze u halen of.. ??

**P** Nee, ik kom nog zelf.

**S** Uhu.

**P** Ja..ja (korte stilte)

**S** En dan maar aansluiten enzo, da gaat allemaal ...

**P** Dat gaat redelijk goed ja. (korte stilte)

**S**  Hebt u ooit al slechte dialyses gehad? ... Of wat verstaat u onder een slechte dialyse?

**P** (twijfelachtig) Neee. Ik heb wel al geweest *opt* einde van euh *da* ik zo *wa* krampen krijg in mijn benen

**S** Uhumm

**P** ... Maar euh ja zebben dan al geweest dat ze eeuhh het *machien* afzetten en euhhmm da ga over dan. Maar dat is altijd op het einde van de dialyse. (lange stilte)

**S** Goed. (korte stilte) De-euhmm.. euhm .. In welke mate merkt u dat er - heeft de dialyse een impact op uw leven dan? Zijn er dingen .. die je zegt `Das voor mij een goeie dialyse want da en *da* is dan beter of *das* een slechte dialyse want das slechter?

**P** (korte stilte) Nee feitelijk ni.

**S** Zijn er dan dingen in in de dialyse die belangrijk zijn voor u? Belangrijker dan andere zaken?

**P** (lange stilte) Nee, euh vroeger

**S** En woont u alleen thuis of..?

**P** Nee me mijne man.

**S** Ja. Kinderen?

**P** Ja, twee maar die zijn wel getrouwd he, en mijn dochter die woont euh in X.

**S** Ja

**S** Eneeuuhmmm u zegt van ja ook bij de buikspoelingen *konde* nog meer ergens naar toe gaan. Merkt u ook dat u .. dat u sociaal leven anders is dan?

P Njah.. feitelijk wel ja, tegen vroeger.

**S** En kan u mij wa voorbeelden geven?

**P** Awel euhh naar de zee gaan [Uhuum] bijvoorbeeld ..

**S** Ging u veel naar de zee?

**P** Ja toch wel regelmatig .. ja.. *da da* gaat nu ni meer, das juist in de weekend, en *da* past dan ni altijd *da ge* kunt gaan euhh ...

**S** En was *da* iets *da* belangrijk was voor u? (korte pauze) De zee?

**P** Ja ik vond *da* wel.. (korte pauze) Ik vond *da* wel..

**S** En was *da* moeilijk om da dannn zo .. minder te kunnen doen? Of is rap makkelijk aanvaard?

**P** Grohnnjah met die peritoneale ik had daar nog aan zee ook. En en dan sloot ik mij ´sna..´s avonds

**S** (korte pauze) Eneeuhmm deuhhmm de verpleegkundigen hier da rondlopen [**P:** Ja]
Zorgen die mee .. heeft *da* dat een invloed hoe dat u dialyse u dia..dia dialyse ervaart? Hoe moeilijk *da da*...

**P** Neee, *da* vind ik goed euhh met dadeeuhh ze daar mee omgaan.. (korte pauze)

**S** En de artsen?

**P** Ook ja, uhuu die doen het goed ja.

**S** Zijn.. Zijn er dingen die de arts goed, *allez* ze kijkt wel vaak naar de bloedwaardes he?

[**P:** Jaaa] Ja wordt u daar veel over verteld bijvoorbeeld? .. Hoe *da* u *waardes* staan?

**P** Euhhhhh awel ja dus iedere maand zo als ze de bloedafnames gedaan hebben, dan komt *den* dokter wel rond enneuuh kijkt ze na of hoe *da* de bloedwaarden zijn.. En ja de laatste tijd euh gaat *da* goed.

**S** En merk je *da* dan ook als ze *da* vertellen? Klinkt *da* dan logisch of voelt u zich ook anders op die moment? Kunt u *da* verschil uitmaken?

**P** Neeje neeje, feitelijk ni. (korte pauze).

**S** En merkt u bijvoorbeeld eh *tis.. wa* we soms als adequaatheid van de dialyse [**P:**Ja] zien he, (korte pauze) voor de arts en verpleegkundige is *da* ook goed *da* die afvalstoffen eruit haalt.

**P** Ja

**S** Merkt u daar verschil van? Of dat die afvalstoffen goed of slecht verwijderd zijn? *Hebde gij* bepaalde klachten die op de dialyse of juist op de .. de afvalstoffen steekt?

**P** (lange pauze) Njaah, nee .. nee (lange pauze)

**S** Euhmm (lange pauze) Als u iets aan dialyse zou kunnen veranderen?

**P** Ja.. euh (korte pauze) Bwaa nee feitelijk ni (korte pauze)

**S** *Ge* kunt u niks inbeelden *da ge* zegt van..`Groh *moest* ik een toestelontwikkelaar zijn of ik zou iets magisch kunnen aanpassen aan de dialyse dat of dat stoort mij het meeste.´?

**P** Neeje..Neeje

**S** Zijn er dingen die u .. die u storen?

**P** Neeje feitelijk ni.

**S** Andere mensen melden soms al de tijd die ze kwijt zijn.

**P** Ja .. ja

**S** Ik weet dat u da ook [**P:** Euhhmm] herkent.

**P** Ja da herken ik wel.. Ja drie of vijf uur *moogde* zeggen *eerda ge* aangesloten zijt en dan *eerda ge* afgesloten zijt.. Ik vind *da* zijn drie namiddagen .. ja .. waar *da* ge niks kunt doe he, *kbedoel*..

**S** En vinde *da* (korte pauze) erg?

**P** Njah .. langs *den ene* kant wel .. ja ma *ge* kunt *ni* anders hé.

**S** Nee (lacht)

**P** Ja....

**S** En hoe gaat u daar thuis mee om? Ik kan mij voorstellen vroeger doet u de buikspoelingen zelf ...

**P** Ja

**S** Of was u man die mee hielp bijvoorbeeld?

**P** Nee, ik deed dat zelf.

**S** Helemaal zelf..he.. Euhmm en nu gebeurt da zo wel thuis en nu moet *uwe* man u missen he [**P:** Ja] Merkt u da verschil ook? *Kunde* daar over babbelen?

**P** Ja.. ja mijne man [11:33]

**S** Is *der iet da* euh.. Wordt daar over gepraat? Of...

**P** Ja ni zoveel.. nee ni zoveel nee..

**S** Kunt u zich *da* nog herinneren op het moment *da* [11:45] er de peritoneaal dialyse, buikspoelingen [**P:** ja] ni meer? [**P:** ja] Dan gegaan naar de hemodialyse. Weet u dat nog, kan u dat moment nog ...[**P:** Njaa..] oproepen?

**P** Ja, das wel ja een groot verschil he tegen *da ge nen* dag thuis zijt.
En wie

**S** En kon u *da* voorstellen op voorhand of waren er toch andere

**P** Ja das wel anders ..

**S** Hoe had u zich *da* voorgesteld?

**P** Ja ja.. die zijn weg ja (lange pauze) ja euhhh voor u aan de dialyse

**S** Hoeveel jaar misschien aan de dialyse moeten, aan de machine in het ziekenhuis.
[**P:** ja] Hoe was *da* beeld *da* u daarvan had?

**P** Ja ik weet dat niet meer he

**S** U had er op voorhand zo niet echt over... een idee van? [**P:** nee]

**S** Kijkt op voorhand?

**P** Jaa (korte pauze).

**S** *Ge* kon u wel iets bij voorstellen? [**P:** Ja] dikke benen voor u naar hier [**P:** Ja] kwam he had u .. had u ook andere klachten die dan beter gegaan zijn? Of andere klachten voor *daje* aan de dialyse *ni* kon of vooral die dikke benen?

**P** Vooral da

**S** Maar dus niets aan u eetlust, [**P:** neeje] u energielevel [**P:** neeje], geurklachten? [**P:** neeje]

**P** Nee

**S** [13:40] Zijn er klachten die u zegt *da* komt volgens mij van de dialyse, ik had daar vroeger geen last van en nu wel en .... [**P:** *da* jeukt] de jeuk? [**P:** ja]

(lange pauze)

**S** Andereeuh dingen **da** je ook merkt?

**P** Nee *ni* speciaal..

**S** (lange pauze) Euhmm.. Heeft u al slechte ervaringen op de dialyse gehad?

**P** Nee *das* altijd hetzelfde geweest..

**S** *Ge* hebt nooit lang of er organisatorisch probleem waren bijvoorbeeld?

**P** Nee

**S** Nooit kapotte toestellen dat problemen gaven?

**P** Ja soms maken ze nu wel *nekeer l*awaai he. (lacht)

**S** En stoort u dat dan?

**P** Moh, *bwa* nee.. ja *ge* zijt *da* gewoon..

**S** Hebt u veel alarmen?

**P** Euh ni zo speciaal, nee..

**S** En als *da* alarm maakt, bent u dan ongerust? Of..?

**P** *Neeje*..(korte pauze) *Neeje* (korte pauze) maar ze komen dan toch kijken of dat er ne..euhh

**S** Ja .. (lange pauze) En de eerste keer *da* je zo een alarm had?

**P** Ja, ze kijken wel, maar ze komen dan direct, ja..

**S** *Ge* denkt *ni* direct van “Ho *wa* is da hier?”?

**P** Nee Nee ni speciaal (lange pauze).

**S** Moet u van ver komen?

**P** Euhmm

**S** Dus *da* vervoer *da* gaat voor u? [**P:** *Da* gaat ja] En moet u vaak lang wachten?

**P** Nee *maja* als ze bezig zijn

**S** Van euh werk wordt er iets gedaan?

**P** Euhhhh ik ben En dan ben ik euh naar hier gekomen naar X. Euh heb ik daar En euh u dialyse is *da* zoals begonnen of? Nee das al een paar ONVERSTAANBARE AUDIO [16:12]

**S** Hoe lang bent u euhhmm al thuis dan? Sinds wanneer? Op welke leeftijd of ...?

**P** Eeuhh....79 ja

**S** Ja en u dialyse is gestart in tweeduizend ennnn... ? (lange pauze)

**P** Euhhmm (lange pauze) in euh (lange pauze) Op het einde van 2000.

**S** Over de jaren 2013

**P** Ja 2013 ONVERSTAANBARE AUDIO [16:57]

**S** *Wa* zijn de zaken die voor u nog belangrijk zijn?

**P** Dat alles goed gaat he. (lacht nerveus)

**S** En hoe merkt u dat? ... Hoe oordeelde dat het goed gaat?

P Oh njah, *ge* kunt daar *ni* zo speciaal iets van zeggen ze.

**S** Zijn er dingen *daje* zegt van “Ik hou *da* in de gaten of ik luister naar *wa* de mensen
zeggen of ..”?

**P** Jaa da wel

**S** En wie zijn die mensen dan?

**P** Euhhh

**S** Andere manieren?

**P** Nee (lange pauze)

**S** Of dingen die u zwaar vallen?

**P** (lacht) Het naar hier komen.

**S** Het naar hier komen (lacht).

**P** Het naar hier komen of ja *ge* kunt ni anders he ***

**S** Ja

**P** das nu al ongeveer drie jaar.

**S** Weet je nog hoe lang .. *Vinde da .. da da* nu een stuk van u leven geworden is?

**P** Awel ja

**S** En voel je ook een dialyse?

**P** Ja. (lange pauze)

**S** En is da..En *kunde* u dan *wa* bezighouden?

**P** Ja, nu de dialyse.

**S** Van de dialyse zelf?

**P** Ja toch wel.

**S** [20:32] zijn er nog dingen euhhh voorbeelden aanhalen van zaken waardoor je het merkt da je aan de dialyse bent of dingen die door de week die gans veranderen? Of anders zijn?

**P** Nee ni speciaal.

**S** Euhhmmm andere sociale activiteiten trouwens die u niet meer kan doen? (korte pauze) Of moeilijk voor familie bijvoorbeeld?

**P** Ja, ze zijn *da* gewoon he *dakik* naar hier kom.

**S** Op andere datums?

**P** Ja

**S** En euh aan dialyse?

**P** Ja

**S** Nog zaken zo?

**P** Nee ni speciaal,

**S** [21:36] Op wat moet u allemaal letten?

**P** Ja zien wat *dawe* eten enzo, groenten euhh *da ge da* *ni moogt* en *da* *ni moogt.*

**S** Als de dokter jou komt zeggen van ja euh u kalium, *uwe* fosfor *da* sta redelijk goed. Is *da* ni belangrijk is voor u

**P** Ja *da* helpt.

**S** En als ze *da* vertellen *wa doeje* daar dan mee?

**P** tsja

**S** En En hoe lost u *da* dan op?

**P** Euhmmm .. ja

(lange pauze)

**S** Zijn er nog andere dingen rond de nierziekten of rond de dialyse of andere zaken die je wilt vertellen?

**S** (lacht) Nu ga ik het stopzetten he.

Interview Patiënt 6:

**S** Goed.

**P** Ja.

**S** Dus euh.. Weet u nog wat er allemaal op die papieren stond?

**P** Nieje, da weet kik ni mier.

**S** Neh, dus het gaat hem er eigenlijk om dat we meer en meer proberen ook de patiënt, in zo de medische wereld, de patiënt in rekening te brengen dus dan moeten we eerst weten wat de patiënten denken hé. En tis een beetje de vraagt wat dat er gedacht wordt door patiënten aan dialyse over dialyse. Maar euhm wat da aan de… Eerst zouk graag een keer gewoon een keer weten hoe da je aan dialyse beland bent? Hoe dat da met uw nier gegaan is, want ik ken u dossier niet hé. Dus euh vertel maar.

**P** Awel euh toen ik vijftien wasss, is er een nier weggenomen.

**S** Uhu.

**P** En euhh… Ja dat, dat was allemaal prima in orde daar achter maar dan kreeg ik euh… suikerziekte.

**S** Uhu.

**P** En ja. (korte stilte)

**S** Oké.

**P** Der.. Derdoor is.. is euh.. die.. die.. nier weeral, de overige…

**S** De andere? Ja.

**P** De overgebleven nier kapot gegaan.

**S** Ja. En bent u al lang aan dialyse dan?

**P** Vier jaar.

**S** Vier jaar en werd u op voorhand dan opgevolgd?

**P** Jah, dokter Van.. Euh professor X. Toen ik naar Y-

**S** Ja.

**P** -kwam weunen, want ik ben wel van Y, maar ik woonde in Z.

**S** city?

**P** Neeje.

**S** (lacht)

**P** (lacht) Y.

**S** (lacht) Oké.

**P** En euh natuurlijk ja, ge voelt dat niet. Het doet geen pijn. En dan is het te laat hé.

**S** Ja. Hoe.. Hoe zijn ze der op gekomen?

**P** Eigenlijk euh mijn bloed wier euh werd euh tis constant.. voor die suikerziekte nagekeken en euuhmm.. daarachter ja. Zeeuuh, zebben da wel de dingen meegenomen voor de euh nieren, ze wisten da ik maar één nier had. En euhh aan.. aan die uitslagen hebben ze het dan gezien dat dat die verminderde.

**S** Ja. Oké. En dialyse hoe gaat u da?

**P** Oh ma ik zen da gewoon.

**S** Weet u dat nog in het begin als u gestart bent?

**P** Ja da had ik wel euh veel schrik.

**S** Kunde daar een beetje over vertellen?

**P** Eneeuuh… Da was maar twee uur de eerste keer.

**S** Uhu.

**P** En ik werd onmiddellijk al ziek (lacht). Ik moest al.. mijn bloeddruk zakte. En mijn.. maar dan is dat weer door de..de weken en de jaren dan. Ik heb.. Tis nodig hé! Ge.. Ge moet er u in sterk..

**S** En is dat iets dat moeilijk is?

**P** Neen, moeilijk is dat niet hé.

**S** Ja der u in stellen wil ik zeggen zo het..

**P** Neenee, ge weet dat da moet en da..da komt hé.

**S** Ja.

**P** En tis hier euh wel aangenaam.

**S** U bent altijd direct hier geweest dan?

**P** Ja.

**S** Ja, oké. Euhm. Het onderzoek zelf gaat over, kweet niet kan u goed Engels?

**P** Nieje, nee.

**S** (lacht) Gaat over “Adequacy of dialysis”. Das een Engelse term en als die letterlijk vertaald gaat dat over adequaatheid van dialyse. Zegt da.. Zegt da woord u iets? Adequaatheid?

**P** Nieje.

**S** Neh, als we het dan in meer mensentaal euhmm..

**P** Ja voilà.

**S** Vertalen hé, kunnen we het ongeveer vergelijken met euh goeie dialyse.

**P** Ja.

**S** Ja en nu is mijn vraag naar u: “Wat vindt u een goeie dialyse? Wa maakt er dat de dialyse goed is?”

**P** Ja euh.. Ik.. vind da.. Das hier altijd hetzelfde, ik we.. Kzou niet weten hoe.. hoe dak da da u kan uitleggen. Das.. Dialyse is een dialyse hé?

**S** Geen verschil tussen de..?

**P** Bwa den iene kier is diene bloeddruk ne keer.. zakt diene das ze ze de euh het vocht op euh afname moeten afzetten, nen andere keer ist niks. Dat is ja euh.. Mor als ge noar huis goet, ist gedaan en.. en ge zijt wel vermoeid mor..

**S** Ja.

**P** Mor ge voelt a ni slecht hé.

**S** Ja en zijn er dingen die het voor u beter of slechter maken?

**P** Pff, nieje.

**S** Kan u zeggen van: “Ik heb ooit een slechte dialyse dag gehad”?

**P** Nieje, kan da ni zeggen. Nieje.

**S** Of een zeer goede dialyse dag?

**P** De euh.. Voor mij zijn ze altijd hetzelfde buiten da de den bloeddruk nekeer zakt. Das niet altijd hé?

**S** Ja, zij-

**P** Tegen da ge naar huis gaat is da allemaal in orde en ik euh voor die heupbreuk kon ik, kwam ik zelfs te voet. (korte stilte) En ik ging te voet naar huis.

**S** Ge moet niet ver?

**P** Nieje tis ni zo ver moja ge euh.. Ik was ver deuje als ik thuis kwam, de eerste uren.

**S** Beetje rusten dan?

**P** Jaja.

**S** Ja en is dat dan iets dat de altijd merkt of..?

**P** Dade als ge, als ge te voet komt? Hebde da eigenlijk ni, kommik me me euh ziekenve.. vervoer.

**S** Uhu.

**P** En nu is da al gans anders hé!

**S** Ah?!

**P** Ik ben moe, maar ik heb die baan ni afgelegd hé!

**S** Ja.

**P** Ik word aan de deur afgezet.

**S** Vinde het beter met vervoer? Of euh..?

**P** Beter? Ja maar als ik koste, als ik.. Normaal als ik..k.k kunnen stappen gelijk vroeger, dan ging kik altijd me euh.. te voet zeh! Maar ja.. Dan beweeg ik en ge zijt buiten.

**S** Ja wat meer vrijheid.

**P** Ja.

**S** Ja.

**P** En wa dak na moe wachten, hé? Of ge moe thuis wachten.

**S** Ja.

**P** En dat is dan het geval ni hé.

**S** Zijn er nog andere zaken die voor u belangrijk dan zijn bij een dialyse?

**P** Ja de sfeer. De sfeer van de mensen, het zijn hier allemaal plezante mensen en de verpleegsters oek hé. (korte stilte) Ja da da da ik da da ik vind ik da dat het bijzonderst.

**S** Ja.

**P** Want als ge hier veel zit euh en tis niemand ni da neki tegen a iets zegt of ge kunt neki ni lachen. Dat is geen… Dat is geen plezante dialyse hé, omdat het zolang duurt en dan gaat dienen tijd voorbij.

**S** Dus een beetje afhankelijk van hoe dade entertaint wordt, - (patiënt lacht)- bij wijze van spreke?

**P** Ja. Als er hier ma niemand iets zegt, oh dan duurt dat toch zoelangk.

**S** En tis ook vier uur waarschijnlijk?

**P** Ja tis vier uur.

**S** Is het ooit langer moeten zijn of…?

**P** Nieje.

**S** Hebde ooit extra al moeten komen?

**P** Nieje, nieje.

**S** Altijd mooi drie keer per week en da lukt?

**P** Ja drie keer per week en da lukt goed hé.

**S** Oké. In welke mate euhm heeft u dialyse u leven, hebde da moeten aanpassen?

**P** Ja het is wel een aanpassing, maar ik ben wel nogal flexibel.

**S** Uhu.

**P** Ennnn… Ja ik zen da rap gewoon. En euh hé?

**S** Uhu.

**P** En ik vond da goed. Tis hier goed en dermee amenikken, voor de rest euh…

**S** En wilt dan zeggen da je da gewoon er.. snel proberen ervaren hebt of..?

**P** Ja. Kheb daar ef… Het moest hé?!

**S** Ja.

**P** Dan aanvaardde da. Allez den iende mens misschien anders als den anderen. Da weet ik nau ni hé.

**S** En ge zegt da de wel wa dingen moeten aanpassen hebt, kunde daar voorbeelden van geven?

**P** Ja gij, da da dieet hé. Ge ge moet a aa..aardappelen twee keer koken. Ge moet a groenten twee keer koken en..

**S** Ja.

**P** Dat hé, ge ge meugt da ni. Da mag ni, da mag ni.

**S** En ist lastig?

**P** Nee omda ik euhm.. ik had euh… Ik zen diabeet.. geworden. En ik euhh tzijn twintig kilo op zes maand afgevallen door het dieet van suikerziekte.

**S** Amai.

**P** Ja en ik zen da gewoon. Tis wel het tegenovergestelde.. Nu.., maar ge..ge moet der ne dingen in vinden hé ja euh . En da goat.

**S** Oké en woonde gij alleen thuis…–

**P** Ja.

**S** -… of hebde gij ne man?

**P** Ik woon alleen.

**S** Dus ge kookt voor u zelf?

**P** Ik kook voor mijzelf.

**S** Ja, hebde kinderen?

**P** Euh stiefkinderen.

**S** Ja oké. Euhmm uuu…. Sociaal leven bijvoorbeeld?

**P** Ah ma ik zit in een service flat.

**S** Uhu.

**P** Enn.. euh.. Ja daar is alles vanalles te doen. Bingo avond euh.. van alles. Ik verveel mij ni.

**S** Allez des te beter. Euhm. Hebt u ooit over “Kt over v” gehoord?

**P** Nee.

**S** Neh. Ja das ni belangrijk. Das eigenlijk allez.. Het is een vraag die we moeten stellen omdat dat iets is dat de artsen soms gebruiken voor die goeie dialyses te meten, maar dat is een formule. Dus euhmm..

**P** Nien, dat euh da ken ik ni.

**S** Neh.

**P** Nieje.

**S** Euhmmm.. Hebde altijd hemodialyse gehad?

**P** Ja.

**S** Nooit buikspoelingen of iets?

**P** Neh, nee, nieje.

**S** En hebde gij een fistel of ni?

**P** Ja.

**S** Ja ne fistel. Hebde ooit een katheter gehad ook?

**P** Neeje.

**S** Nee, ge hebt altijd.. Zebben op tijd uwe fistel kunnen aanmaken?

**P** Ja twee jaar op voorhand.

**S** Dus ge had het echt wel van ver zien aankomen?

**P** (lacht) Jaah.

**S** En content van?

**P** Mjaa, bajaa.

(korte stilte)

**S**  Oké. Ben nu aan het denken of dat ik nog iets vergeten vragen ben… Moest ge iets mogen veranderen aan de dialyse? (korte stilte) Ge hebt ne magische stok ofzo.. (patiënt lacht) Of ne geest die één wens kan doen? Ma ge kunt niet wensen dat het niet meer is hé, maar ge kunt er iets aan veranderen.

**P** Grohja wa kunde daar aan veranderen?

**S** Hetgeen dat u het meeste stoort aan de hele tijd dialyse.

**P** Ik hoop dat die fistel het uit houdt, da ik geen… want da zou ik ni geirne hemmen.

**S** Nee?

**P** Nee.

**S** En hoe kommet?

**P** Euuhh… Ge kunt ni mier in bad. Ge kunt a gienen douche pakken.

**S** Ja.

**P** En ik hem da geirne dak ma kan.. Ja kan ma, kgoen oender twoeter en tkan gien kwoed.

**S** Ja.

**P** En dan moeije da. Datte da houdt ma miest tegen derveure hé. Veur nen.. nen katheter.

**S** U bad en u douche?

**P** Ja. Jaa zie ge kunt a ni mier proper wassen gelijk dat moet. Da vindekik hé.

**S** Hmmm.

**P** (Lacht)

**S** Ma ge hebt uiteindelijk nooit eentje nodig gehad hé dus..

**P** Ja ik euhnn.. Ik zen content me manen fistel. (lacht)

**S** Dus ge zou wensen da ge hem kunt houden?

**P** Jah! (Lacht) Ik hoop het.

**S** Oké. Goed. Euuhmmmm… Ben nog wat aant denken. Hoe oud bent u?

**P** Achtenzestig.

**S** Achtenzestig. Oké, hebben zij… Als u begon was u vierenzestig waarschijnlijk hé?

**P** Ja.

**S** Ja. Hoe zit dat met een transplant?

**P** Wel, ik weet het niet. Ik heb de voor- en de nadelen een beetje naast elkaar gelegd en euhh… Hoelang hebte ee..e.. een..een nier? Een getransplanteerde nier? Twaalf – dertien jaar?

**S** Afhankelijk hé.

**P** Allez ongeveer. Ongeveer. En ten derachter weer die dialyse? Nieje da zouk ni kunnen.

**S** En ge zou het niet kunnen om der af te zijn en dan terug te moeten starten, wilde zeggen?

**P** Ahja want e..ee..een virus, hé? Hier is een maddammeken me een ulce… Ze had enen en twas oek gedaan en nu omda ze da virus hemmen en dat heb ik goort. Ja dat hem ik dermee mee afgewogen hé. Tkan al ni hé.

**S** Ja.

**P** En nu..

**S** Ge wilt het risico niet nemen?

**P** Ohgeilijk nie. Ahj, da das misschien gien risico ten misschien tien twelf joer goe leiven, mo kemmekik noa oek een goe leven.

**S** Ja, oké. Tja.

**P** Ik.. Ik hem ma der in gesteld en dermee is da goed.

**S** Ja.

**P** Met het veurdiel as…as ge iets.. iets veuren hebt, als ge noa een beroerte ofzo krijgt en.. en ge zijt echt… Dan kunde alt, as da vastligt, zeggen gien dialyse ni mier.

**S** Hebde da gedaan?

**P** Ik moe da nog doen, maar ik zen da wel van plan.

**S** Om zo wa dingen voor u zelf..?

**P** Ja. Da ge zegt hé, ik wil ni..ni een ander ten laste zen en zu echt da ge ni me niemand ni niet meer kent ofzu. Dan vindekik da ge kunt stoppen met de dialyse.

**S** En is dat iets da de kunt goe bespreken met u dokters?

**P** Euh ik heb da met dokter X besproken en euh…

**S** Ja, gept er goed over nagedacht?

**P** Ja ik hem er over nagedacht en wel omdak ier zu al zuu veel echt oaw mensen hem. Allez ge zit hier, der zat hier een madammeken zonder bienen en gevallen me me een blauu gezicht, want ze viel voorover in eure rolstoel. En a ge da allemaal ziet… Nieje en dan emmek, da z.. da zoukik nog ni willen. En blench en zuu dat ier ge… Ge ziet dat hier den oek hé, me die mensen die ier gelegen hemmen. A ge den begint noa te peizen.

**S** Ja.

**P** God nee da willek ni.

**S** En wat doet da dan me u? Als ge zo van die mensen ziet dade denkt van..?

**P** Jaa, gept gij der daar.. Ge kent die mensen den.. Dat is wel.. Ja euh.. Pff, da pakt a wel. Da..da.. Ja.

**S** En kunde daar dan me iemand over babbelen?

**P** Oei nieje da..da moe ni euh… Ik weet ze zen den oud of zemmen er zelf voor gekozen en als ze gelukkig zen. Baja want ze weten het wel nimier mor. Ik klap ze, ten.. ten zijje oek gelukkig hé? Als ge ni meer afziet?

**S** Mmh.

**P** Doer, dade ja..

**S** Ja..

**P** Das een fout hé, das gien.. Der moeije ni over klappen. Allez ik ten toch..

**S** Ja. Ja maar ik dacht ook.. Ik was in het idee da je bedoelde sommige.. al gestorven waren. Ik bedoelde over de mensen als er iemand gestorven is of dade daar dan kunt over babbelen met de rest van hier.

**P** Ahjamaja! Oh nee we zeggen weur neki tegen ien: “Zis overleden”. Ja da mens is gelukkig op een manier. We meugen da ni tegen de familie goan zegge, mo onder ons zu.

**S** Ja.

**P** Ja.

**S** Wordt erover gebabbeld over zo..?

**P** Nieje eigenlijk nie. Nieje.

**S** Of over eventueel stoppen ofzo op voor?

**P** Oh nieje nieje nieje dei.. de..di… Ik goan het a zeggen ik goan stoppen hé want ge.. euh.. ik euh.. Ge moet tegen die mensen over plezante dingen klappen geluuf ekik hé.

**S** Ja. Ahajajaja.

**P** Of vertellen wat da ge…

**S** Ja.

**P** Ma weur vertellen azu ewa over van alles en nog wadde.

**S** De koetjes en kalfjes eigenlijk?

**P** Jaa..

**S** Ja. Ma moeste met iets zwaar zou zitten, zoude da meer voor uzelf houden of nekeer met den dokter?

**P** Awel jaa of euh.. of… ja of..

**S** Omda de vindt da de..?

**P** Ik hem faitelijk.. Ik zen.. Ik ik hem zu gien karakter voor da aan een ander.. Ik ga da wel veur mazelf, want ik kan da wel goed relativeren, ziejet?

**S** Ja.

**P** Het is dermee, ik.. ik..kgoan niemand euh als ge miserie hebt. Ik go da aan een ander ni goan vertellen hé. Gelijk allez mergen ist weer nen dag en da zal wel beter goan en voilà. Ge moet da zo euh..

**S** Ja, iedereen zijn eigen.

**P** Da noemen ze da ongegeneerd.

**S** (lacht) Uhu.

**P** Ja hé?

**S** Ja. Oké. Voorlopig gaat het goed du**S**hé?

**P** Mahbaja.

**S** Oké.

**P** Ik.. Ik zal a is iets vertellen hé. As er den dingen.. Den euh.. Ma geld heet, as ik gevallen zen.

**S** Ja?

**P** En da was oep stroet hé. Zemme ma der geweest oep stroete.. Ja op..oproapen azu zeggen zeiler da. En da woaren twie ambulanciers ik kost pompiers. Ba mijn broere is oek ne pompier.

**S** Uhu.

**P** En.. Ge goet dermee lachen ma ik zou der van de ziere echt… En ik was… Kwas deur aant lachen hé, met die mannen want ten was da, ah ja zadde man bruure ten gebeld: “wemmen a zuster hiere vant stroate geroapt.” En ik kost er ni aan doen hé, ik moest ermee lachen. (Lacht) “Da hemme we toch nuut ni meegemoakt, zeet eur heupe gebroken en ze ligt hier te lachen.” Azuu een karakter hemmekik.

**S** Ja. Ahja dat is wel.. Ni veel mensen zouden lachen met een beenbreuk denkik.

**P** (Lacht) Ja.

**S** (Lacht) .. Ja.

**P** Maja da.. veur ma was da komiek hé. Gelijk da ze deur oan dienen telefon gedoen he..

**S** De absurde van de situatie. Zebben u dan naar hier gebracht ook of euh?

**P** Ja. Ja ze zijn ba ma gebleven tot da maan bruur hier was.

**S** Waarschijnlijk gewoon omdade een plezante waard om bij te zitten hé? (lacht)

**P** (Lacht)

**S** Ni over pijn a..-

**P** Ma ik kost ze al van als ik euh… Van als ze zeeuulf klaine weuren zal ik ma zeggen hé. Want das azu een vriende clubke allemaal ba de pompiers gegoan en man bruure en ge kende der van.

**S** Hmmmm.

**P** En dermee ja da was al vertrouwlijker als dat er der vremden -

**S** Ja.

**P** - kommen eee.. en ja ze dan kennen hé en weten dant euh.. dant van die komieken zen ja.

**S** Das al lang geleden?

**P** Euh.. In februar.

**S** Ah recent en gaat het nog vooruit me u been?

**P** Ochja hij heeft er geen nieuwe heup ingestoken hé. Hij heeft er euh.. Hij heeft da-

**S** Me een pin ofzo?

**P** Me een pin en da duurt veel langer hé want as ge der misschien zelf iets aan te zeggen hebt, kunde zeggen ja alstamblief geeft ma een nieuwe heup want de mensen me een nieuw heup die die op een moand.. Zieje da ni meer. Da zieje wel en ik zit hier nogal te manken en te manken.

**S** Ma tzou moeten beter gaan?

**P** Ja.

**S** En vinde da ge hier iets te zeggen hebt?

**P** Hier iets te zeggen? Ja ik.. Ge moet gij hier aagenlijk moeijie niks te zeggen hemmen hé? Ge wordt hier bediend, ge krijgt a eten-

**S** Ah ja zo.

**P** Ze doen alles voor a. Nieje, tis hier goed. Echt woar, allez ik vind dat hé.

**S** Ma tis ja, ik heb de indruk da iedereen goed overeenkomt hé?

**P** Ahjajaa.

**S** Oké, merci alleszins voor het gesprek.

**P** Tis niks.

**S** Kga ne keer op stop zetten.

- EINDE OPNAME –

Interview Patiënt 7:

**S**  Euhm.. En dan ga ik ook wa nota’s maken tijdens da u aan het babbelen bent zodanig dat ik u ni moe onderbreken, moest ik iets extra willen vragen ofzo. Alles wordt euhmm.. Confidentieel behandeld, dat wil zeggen da.. da we nooit namen ofzo of..-

**P** Ja. Het komt nooit op Facebook?

**S** - dat het allemaal anoniem is.

**P** (lacht)

**S** Het komt nooit op Facebook bijvoorbeeld, op al die andere dingen dien Instagram enzo allez..

**P** (lacht) Jaja ma ik heb gene computer, nenee.

**S** Ge hebt geen.. (lacht) Ja awel ma ook al hebt ge der geen, ge gaat er zeker -

**P** Nenee.

**S** - zeker ni op verschijnen. Het enigste is.. Dus da dat er wel van doet is soms artikels maken hé, met de resultaten. En natuurlijk het kan zijn dat je u in sommige van de dingen herkent, maar misschien da verschillende mensen bepaalde meningen delen zoals u. Maar daar gaan ze nooit kunnen weten da gij da gezegd hebt.

**P** Jajajaja. Tis naamloos.

**S** Tis naamloos.

**P** Jaja.

**S** Voilà, goe? En tgaat dus ook een beetje over hoe dat de mensen de dialyse ervaren. Maar kzou eerst keer willen weten of vertel anders een keer hoe da ge aan dialyse ge..terecht zijt gekomen want ik ken u dossier natuurlijk niet.

**P** Nenee.

**S** Hoe komde gij van.. Wa is er me u nieren gebeurd en…?

**P** Wel ik ben opgenomen in..in de spoed.

**S** Uhu.

**P** En euhh.. zebben euuhh.. vier overbruggingen gemaakt.

**S** Uhu.

(korte stilte)

**P** En de euhh.. Den eerste nachten zijn de nieren geblokkeerd.

**S** Dus na een operatie?

**P** Nieje voor de operatie nog.

**S** Voor de operatie?

**P** Voor de operatie nog zijn de nieren geblokkeerd.

**S** En voordien?

**P** Nee maar ik gaan er u wel bij zeggen. Mijn zuster is al euh een nieuwe nier, mijn schoonbroer,.. (lacht) Ik ben den derde.

**S** In de familie?

**P** In de familie ja.

**S** Dus ge wist op voorhand da de da…-

**P** Jaja.

**S** - Het ging, allez op voorhand.

**P** Jaja maar zij hebben thuisdialyse gedaan.

**S** Uhu.

**P** Dade.

**S** Ja.

**P** Waarom da ik noa allier.. Ik ben nen alleenstaande hé. Nooit ni.., ik ben niet gehuwd.

**S** Ja.

**P** Dermee doe kik hier de dialyse

**S** Ja.

**P** In plaats van thuis.

**S** Ja.

**P** Want ik hem het genoeg gezien thuis bij mijn zuster en bij mijn schoonbroer hé. Hoe dat het in zijn werk gaat.

**S** Ja.

**P** Tis.. tis zo gebeurd, met op de spoed opgenomen te worden.

**S** Voor u hart?

**P** Ja.

**S** En wanneer was dat?

**P** Moet dat de juisten datum zijn?

**S** Nee jong.

**P** Tweeëntwintig september.

**S** Is da..

**P** Ik denk dat u iets is. Tweeëntwintig september.

**S** Van dit jaar nog maar?!

**P** Euh ja verleden jaar..

**S** Tweeduizendzestien nog maar?! Jaja.

**P** Ja zestien.

**S** Dus eigenlijk allemaal recent?

**P** Ja jong ja.

**S** Ja. En daarvoor ooit nierproblemen gehad?

**P** Neen.

**S** Andere zaken da de zegt van…?

**P** Neenee.

**S** Twas voor u een grote..?

**P** Ik.. Ik hem nooit geen.. Ah ik heb nooit geen problemen. Ik zat in.. in.. in misschien tien jaar ni meer bij den dokter geweest.

**S** Ja. Ja dus zeer plots op da moment?

**P** Ja. Ja.

**S** En was da goed voor u denkt u? Da je al wist wat da dat inhield? Da de daar al.. Wa da je daar al gezien had?

**P** Awel ja euh.. Ik.. Kwas der al van op de hoogte dus ik wist wat dat er allemaal ging gebeuren.

**S** Ja.

**P** Maar hier had ik nog nooit geweest. Ma.. Maar thuis. (korte stilte) Thuis bij mijn zuster had ik het gezien hé.

**S** Ja.

**P** En mijn.. Mijn schoonbroer heeft na een nier, zes maand. (korte stilte) En bij mijn zuster ist van tweeduizend ennn.. zeven.

**S** Amai.

(lange stilte)

**P** Ja.

**S** En hoe steldet?

**P** Redelijk goed. Redelijk goed.

**S** Kunde zo een beetje vertellen over hoe da da begonnen is, als ge gestart zijt? Weet ge da nog? Hoe da de u voelde enn..? Als ge int begin dialyse gestart zijt?

**P** Ja, jajaja.

**S** Ja, weet ge nog hoe dat dat was?

**P** De dialyse is gestart in Aalst hé.

**S** Ja.

**P** Ik ben in Aalst gestart en ik heb daar een week.. en dan ben ik langs hier gekomen.

**S** En hoe.. Hebde daar.. Voelde daar iets van? Hebde daar last van?

**P** Ik heb daar gene last van.

**S** Neh.

**P** Zieker gene last van. En of dat da beter of slechter is dan. Bij mij veranderd er ni veel. Alsk hier toegekomen was en naar huis gaan.. (korte stilte) Nee.

**S** Nee? En moeten zij vocht onttrekken bij u? Nee hé? Moete gij ook zo altijd vocht af of ni echt?

**P** Moete kik dienen “Burinex” hé voor…-

**S** Ja!

**P** - Thuis.

**S** Maar tis ni iets dat er twee kilo moet afgetrokken worden aan dialyse ofzo?

**P** Nieje.

**S** Gij plast nog goed?

**P** Jaja jajaja ik plas nog goed jaja.

**S** Ja. Oké. Dus eigenlijk u gewicht met den “Burinex” ongeveer in orde?

**P** Ja ja ja ja ja…

**S** Der moeten geen kilo’s af?

**P** Nee nee nee neeje nee neeje.

**S** Nee.

**P** Nee.

**S** Oké.

**P** Tis altijd rond zestig kilo.

**S** Voor en na aan dialyse?

**P** Jaja.

**S** De studie op zich..-

**P** Ja.

**S** -gaat over de “Adequacy of dialysis”. Das een Engelse term, ik weet niet of dade gij goed zijt in Engels? Of dat dat u iets zegt?

**P** Neeje neeje neeje neeje da ken ik ni goed.

**S** Als ge dat vertaald int Nederlands is dat “adequaatheid van dialyse”, maar das nog altijd natuurlijk (korte stilte) niet het meest standaard Nederlandse woord hé?

**P** Jaja.

**S** Kunde u daar iets bij voorstellen? Als da iemand zou zeggen tegen u van de “adequaatheid van de dialyse”? Wa zou dat voor u..?

**P** Neh neeje.

**S** Euhm awel als ge dat een beetje vertalen naar wa mensentermen euhmm… Kunde het meest, we hebben er lang over gedacht…-

**P** Jaja.

**S** -Het meest simpel vertalen door een goeie dialyse.

**P** Een goeie dialyse?

**S** Dus de vraag is ee.. een beetje naar u toe. Wa is voor u een goeie dialyse? Welke dingen maken voor u da een dialyse goed is?

(korte stilte)

**S** Ge moogt op u gemak nadenken ze.

**P** Ahjaja. jaja langk en goe. Das een moeilijke vraag ze dade!

**S** Der zijn geen slechte of foute alle.. goeie of slechte antwoorden.

(lange stilte)

**P** Ja een goeie dialyse als er ni teveel alarm is.

**S** Niet teveel alarmen?

**P** Ahja.

**S** Ja.

**P** Denkt ik allez..

**S** En waarom is da voor u dan belangrijk? Of is da?

(korte stilte)

**P** Belangrijk? Awel ja euh.. ja. (korte stilte) Dat is teken dat alles goed verloopt hé?

**S** Ja.

**P** (lacht)

**S** Oké. Jaja.

**P** Das da..-

**S** Dat het hier niet stil staat?

**P** Ja! Want als het alle halfuren in alarm is euh.. Das geen goe teken hé?

**S** Ja oké. Euhmm en is dat iets da de zelf ook op let? Dan..?

**P** Jaja. Ja da wel, da wel.

**S** Dus als er alarmen zijn..-

**P** Jaja. Ik zorg van zo stil mogelijk te liggen of ja nekeer zo te draaien of ten.. Want nu ist weer de scherpe naalden, dan moenik opletten.

**S** Euhmm.. (korte stilte) Andere zaken?

(dialysetoestel gaat in alarm)

**P** Ja voilà zie, kep teveel gedraaid. (lacht)

**S** (lacht) Ge wou het gewoon voortonen zegt?

**P** Ohneeje neeje. (praat tegen verpleegkundige: “Tis ne lichte alarm zeker?” verpleegkundige: “Ja.”) Ziejet? Jama kem teveel gedraaid en en ge. ge.. getoond hé.

(dialysetoestel blijft in alarm*, verpleegkundige: “Kzal hier is efkes komen ajusteren sie.”)*

**P** Ajusteren allez.

(*verpleegkundige: “Ajusteren”*, dialysetoestel stopt, *verpleegkundige: “Alstublieft”*)

**P** Danku.

**S** Tis al gedaan zeh.

**P** Jaja tis al ged… (korte stilte) Zo erg ist ni.

**S** Zijn er nog andere dingen da de zegt..?

(lange stilte)

**P** Nee ik kan zo ni direct niks.. niks.

**S** En als ge dan weer een beetje anders zou zeggen. Wa is er belangrijk aan een dialyse voor u?

**P** Wat is er belangrijk aan een dialyse?

(lange stilte)

**S** Wa vinde zelf belangrijk?

(lange stilte)

**P** Ja das oek.. Zo weinig mo… allez zo weinig.. weinig mogelijk vocht uitgetrokken wordt.

**S** Uhu.

**P** Dat is.. al een goed teken. En dat niet teveel alarm is hé.

**S** Ja euhm.. Hebde soms dat er veel vocht uitgetrokken wordt?

**P** Neen. Neen, ik denk het niet.

**S** Ma ge vindt da-

**P** Ma tis nooit ni veel bij hé.

**S** Nee, maar da houdt ge ook in de gaten?

**P** Jajajajaja.

**S** En waarom?

**P** (lacht) Voor het een beetje op te volgen hé.

**S** Ja om te zien-

**P** Jaja.

**S** Wa wilt da voor u zeggen zebben u veel of weinig vocht zou moeten afgetrokken worden?

**P** Wat dat wilt zeggen? D..D..Dat ik goed euh.… thuis goe mijn dingen doe hé.

**S** Ja dus voor u is da zo-

**P** Jajaja jaja moest dat volledig vol zijn, zou ik teveel drinken.

**S** Ja.

**P** Of.. Alle gienen sterken drank hé want drinken is sterken drank, maar kbedoel euh..

**S** Ja.

**P** Teveel drinkt.. Kan da goed naar omhoog goan. Kep nog tprobleem nog ni gehad.

**S** Ja maar dat is voor u zo een beetje den dingen-

**P** Ja.

**S** - waar da ge op let?

**P** Ja.

**S** Om te zien of dat het goed gaat?

**P** Jaja.

**S** Ja.

(Lange stilte)

**S** Andere zaken? (Lacht) Ik ga da ettelijke keren vragen zeh.

**P** (Lacht)

**S** (Lacht)

**P** Kzie direct zo niks nee.

**S** En mag ik dan zeggen dat het voor u wa belangrijk is aan een dialyse is eigenlijk dat ze goed gaat?

**P** Ja.

**S** En wadde in de gaten houdt is u alarmen-

**P** Ja.

**S** - en u vocht?

**P** En u vocht ja jajaja.

**S** En zijn er andere dingen..? Strikt genomen buiten u.. Dingen die met dialyse te maken hebben? Die voor u ook belangrijk zijn?

**P** Mijn gewicht, mijn gewicht houd ik ook int oog.

**S** Ja.

**P** Dat wel. Dat wel. (korte stilte) Dat wel, jaja.

**S** Dus de.. de.. puur objectieve dingen zo?

**P** Jajaja.

**S** Ja en nu vertelde dat u zus hemodialyse euh.. thuis heeft gedaan.

**P** Thuis..thuis dialyse ja.

**S** Euhmm. Hebde dan zelf ook zo.. daar over nagedacht of allez..? Ge vertrekt met een andere kennis natuurlijk hé?

**P** Jaja. Kwas al euh.. een beetje op de hoogte van…

**S** Ja.

**P** Tis wel ni, hier ist bloed doen maar.. maar thuis ist me water hé.

**S** Ja.

**P** En dermee was ik al redelijk op de hoogte. Ma khad het ni verwacht, khad het ni verwacht.

**S** En hebde zelf ooit overwogen om ook zo die thuis te doen of nooit?

**P** Nieje nieje.

**S** Neh.

**P** Daar hemmik ni over ge..

**S** Omwille dat ge alleen zijt dan?

**P** Ja omda ik het.. Tis een beetje moeilijker hé?

**S** Ja.

**P** Want.. Want kunt nooit ni weten als er een ongeval, allez iets voorvalt en ge stoet doer alleen.

**S** Ja.

**P** Mijn.. mijn zuster woont wel ni verder ze dan ni hoor. Dan moete ze al.. al. alle uren en vier keer per dag kommen kijken hé? Want tis vier kieren en ’s nachts en automatisch jaja. Kep er euh.. allemaal al meegemaakt hé.

**S** Hebde er over nagedacht of dat de ooit thuis zou willen?

**P** Nieje nieje nieje nieje.

**S** Twas voor u wreed duidelijk?

**P** Dade ja. En en b…m…mijn zuster heeft direct gezegd hier. (lacht) Twas direct. Neneeje dat is te moeilijk. Tis te moeilijk. (korte stilte) Maja als ge me twee thuis ist be.. ist..das gemakkelijker. En tin d’ogen houden en.. Ik zeg a..

**S** Ja.

**P** Als ge vier vijf keren ’s morgens. ’T ’s morgens , ’t ’s middags moe komen controleren.

**S** Ja.

**P** Dan ist beter hier hé.

**S** En is u leven veel veranderd met de dialyse te moeten starten?

**P** Pff ja.

**S** Kun je daar wat over vertellen?

**P** Ne.. Veranderd, i..in die mate ik ben giene stillen hé. Ik kan geen vijf minuten stilzitten. Dat is hier een.. Tis al een beetje verbeterd, maar vier uur zitten das ni voor mij hé. En da.. dat is het gruutste euhh.. euh probleem… Voor mij.. En thuis ben ik altijd…

**S** In de weer?

**P** In beweging jaja. En dermee da ik mijn krant meebreng want anders..

**S** Ja.

**P** Vier uur is lang hé? Keg het, tis al verbeterd want ik was wreed zenuwachtig. Ik kan.. Ik kan ni.. Kzeg het ik kan ni blijven zitten.

**S** Ja tis-

**P** En der blijven zitten.

**S** Tis dan meer ongedurig zijn sowieso?

**P** Ja ja.

**S** Los van de tijd eigenlijk?

**P** Los van de tijd ja.

**S** Moest er een toestel bestaan waarbij da je kunt rondlopen, zou je da liever hebben?

**P** Ahja! (lacht) Direct!

**S** Awel ma iedereen zo dezelfde weh?

**P** Jajajajaja.

**S** Nog altijd vast in..in..int centrum maar ge kunt..

**P** Jajaja ik be.. Ik versta wa da ge bedoeld. Jaja. Da wordt ook al beter ja. (lange stilte) Ja tis.. Ik zeg tis ni gemakkelijk.

**S** Tis ook nog ni lang hé? Uiteindelijk..

**P** Nee. Awel van in september euhh.. verleden jaar.

**S** En-

**P** En drie keren per week is oek.. Is oek veel hé?

(lange stilte)

**P** Ge kunt er niks aan veranderen hé?

**S** Nee, moest ge iets aan dialyse kunnen veranderen? Is om hem beter te maken, heel het systeem? En ge moogt gelijk wa zeggen, wa zoude…?

**P** Ah moest het mobiel zijn. (lacht)

**S** Mobiel zijn effectief ja.

**P** Ja.

(lange stilte)

**S** En ge zegt da de alleenstaand zijt. Hebde.. Bent u ooit getrouwd geweest?

**P** Nee.

**S** Nee, ge hebt dus ook geen kinderen dan waarschijnlijk?

**P** Nee, ahnee.

**S** Jama da wilt niks meer zeggen-

**P** Jajajaja eenentwintigste eeuw jaja.

**S** (Lacht) En euhh-

**P** Neenee.

**S** Maar euh u zus woont ni ver hebde gezegd?

**P** Nieje nieje nieje nieje.

**S** En hebde daar dan wa steun van of..?

**P** Jaja jaja dat is.. Dat wel.

**S** Ja en kunde zo nekeer u…uw klapke doen alsde zegt van..?

**P** Ja das iets anders.

**S** Ja?

**P** Ja.. Awel ja klapke.. kwa..kwabke.. klapke da wel, maar ze zegt altijd: “Ge zijt dezelfde als u vader, ge zegt niks”. Als ik binnengekomen ben ook, da was veel te ver al hé.

**S** Ja.

**P** Das veel te ver al. Kem.. Het.. Twas oek op het nipperken, dak op de langen duur gezeit hem ie..hier klopt iet ni hé.

**S** Had ge iets gevoeld op voorhand of ni?

**P** Oei, ik..ik moet een.. een..een dingen een allez.. (lange stilte) De.. De nacht, de nacht ervoren hé. Een hart euh allez.. Een hart dingen… Euh.. Euhmm..

**S** Een infarct?

**P** Een *infract* gemaakt hem. (korte stilte) Der achter hem ik.. hem.. hemmen ze mij da gezegd. Ik moet een *hartinfract* gemaakt hemmen. Maar ik weet het ni.

**S** Hebde het ni gevoeld?

**P** Neeje kheb het ni gevoeld. Kheb juist moeten o..over..overgeven diene nacht en.. Nee ik help veel in ee..een parochiale zaal hé ne ik ben den laatste naar huis gegaan, maar das ne redelijken berg. Ik ben bekan ni boven geraakt en ik heb thuis der nen heletijd ge.. Waarschijnlijk moet ik dan een *hartinfract* gemaakt hemmen. En ’s anderdaags.. ’s Middags ben na.. Ben ik bij mijn zuster gegoan.

**S** Tis u zus die u binnen gestuurd heeft?

**P** Ja. Dak gezegd, ik hem gezegd ik hem een longontsteking, maar twas geen longontsteking. (lacht) En zebben dan vier overbruggingen gemaakt.

**S** Ja.

**P** En direct dialyse oek hé.

**S** Twas van den eerste, lang ni na de dokter maar.

**P** Jaja. Kzeg het, in jaren bij den dokter ni meer geweest en ben ik.. En geen medicamenten genomen, nuut gedoen.. En nu een hiel serie.

**S** Ja en nu ieje drie keer per week nen dokter alsde wilt.

**P** Ja.

**S** En ge zegt van een klapje doen wel ma da de u zus zegt da ge ni open zijt. Hebdet moeilijk om over dingen te praten?

**P** Nieje nieje nieje nieje nieje dat niet. Maar kwil maar zeggen i..ik ben ni gaarne van iemand te laste. Ge..

**S** Ja.

**P** Gelijk hier oek, ik wel aa..aj..ann.. wel zeggen héhé kalm. Ziejet da.da..da da ze gien problemen me..me mij hemmen.

**S** Ja zelfstandig.

**P** Zo..zo..zo ben ik een beetje.. Verstoe jet?

**S** En was dat dan ni moeilijk in het begin?

(korte stilte)

**P** Da was zeker moeilijk. Het is begonnen in X hé. Da dat ik daar in X lag, hemmen ze dat daar gedaan. (patiënt mopmpelt) Ja dak “Speedy Gonzales” was. Alst gedaan was dak hier buiten liep. Maja als ge hier vier uur zit, ni.. nie mijn gewente van.. van azo vo vier.. vier uur te zitten hé.

**S** En doede dan zo nog iets na da de…? Als ge gedaan hebt?

**P** Ja.

**S** Doe je nog iets thuis of..?

**P** Aah thuis u, ja tzal ni zijn, ik weet zulder werken ook. Ik be.. ik bestel bier, ik…iik..ik kgoa..aa..aall.. al de stock aanvullen en al ofzu. Ben regelmatig bezig hé.

**S** Ja.

**P** E..en de vrijdag heb ik der nu een petanquebaan gaan halen en dan komen ze allemaal spelen. Ook de geburen hé.

**S** Ja.

**P** Ziejet e..en kmoet oek al het bier aa..aanvullen hé al ofzu. Ben regelmatig bezig. Ik ben gienn.. iemand da uren kan zitten. Kan da ni. Kan da ni. Ee.en in de fabriek dak gewerkt heb was, twas van onderhoud twas ginder gi.gin..in..in..Inderlopen.

**S** Ja.

**P** Ik ben da gewend hé.

**S** En hebde da dan.. moete dan veel aanpassen ook met de dialyse want..?

**P** Awel ja.. Aanpassen dat wel hé zu gelijk nu de stock moe nu opgemaakt worden. Da kan ik de maandagmorgen ni meer hé. Dus moenik da ten van de namiddag doen, dat is wel en..en den donder.. den donderdag leveren ze bier dus de vrijdag moeten ze ni komen want ik ben ni thuis hé. (lacht)

**S** Uhu. ma ge probeert nog al..alles te doen wa je voordien deed?

**P** Jajaja.

**S** Ma op korteren tijd?

**P** Jaja. Awel ja, ma den tuin dat heb ik laten vallen.

**S** Ja.

**P** Want da..da. da is.. Da doe ik ni meer. Ik doe natuurlijk nog wel euh.. de euh.. de pelous onderhouden en alles dat allemaal wel. Maar den tuin doek ni meer. Dat is.. Twas gene grote maar kept laten vallen, dat heb ik laten vallen.

**S** Hebde er spijt van?

**P** Nieje want twas al..altijd de aa…aardappelplaag, aardappelplaag en dan zu een bollekes, alle kom.

**S** Twas ne moestuin?

**P** En kep gezegd.. ja kem gezegd foert hé. (zucht) En verleden jaar lag ik nog int hospitaal, no..nog ni uit nog ni gerooid warend..En dermee kzei nejeneje. Kdoet nimeer.

**S** Maar ge hebt eigenlijk gekozen voor het geen dat ge het minst leuk vond of allez ja? Ist..?

**P** Awel ja jui..juist mijn uin heb ik laten vallen-

**S** Ja.

**P** -ma meer, meer ni hé?

**S** Nee.

**P** De rest doe ik nog gelijk ast vroeger hé. Nog allemaal gelijk ast vroeger. Jajajaja. Ma..Ma kwoon hier ni ver hé. Ziede want ik kom me mijne.. me mijnen bromfiets euhh langs hier hé.

**S** Ah ge komt zelf?

**P** Jaja ik kom zelf. (korte stilte). Dan had ik hetzelfde probleem, als ze hier met het vervoer me euh een uur zitten te wachten. Pfff nieje. Nieje nieje nieje nieje. Nieje me mijnen brommen en op vijf minuten zenk na thuis.

**S** Ja.

**P** En anders zat, zit ik te wachten, te wachten, te wachten. Nieje dass..Te zenuwachtig hé? En azu gept het misschien al gezien? (lacht).

**S** Nee.

**P** Dak een beetje..

**S** Nee eigenlijk ni.

**P** Hmm, jaja. Ma kzeg het ik ben al een beetje verkalmd want vroeger.. Int begin wast hier slechter. Jaja.. Kan der niks aan veranderen hé? Tis vier uur en tis vier uur hé.

**S** Ja.

**P** Dat isss… (zucht)

**S** En moesten ze nu den tijd willen verlengen?

**P** Verlengen?! Hejawadden.. K.ik..ik kom s’morgens binnen en mijnen bloeddruk is dertien misschien.. En een kwartier voor dak ga vertrekken dan ist eventien achttien.

**S** Danneuhh..

**P** Jaja. Het bloed..

**S** Ja, de reden dak da vraag is omdat sommigen mensen moeten langer draaien hé

**P** Jaja kweet het, kweet het, kweet het.. Kweget wel.

**S** Ik vraag mij af wat u..u reactie of hoe da de der op-

**P** Ja de..de.. da zou ni voor mij zijn allez tzou ni goe, ni goe, ni goe meevallen da de. Nog nekeer een uur bij, neejeneejeneeje.. (mompelt) Tis ni van..van den dienst da..da.. Tis dat ni hé. Tis.. Tis die..diejen tijd die te langk is. Moesten e morgen zeggen tis twee uur. Ik spring direct ne meter hoog. (lacht) Nieje ma theeft niks te maken me.. me..m..me d..d..den dienst hier hé. Tissss den tijd, den tijd.

**S** Het vasthangen?

**P** Ja ja ja.

**S** En hebde dan ooit gedacht aan nachtdialyse bijvoorbeeld?

**P** Nieje. Ah neeje want dan moete volledig euhhh… een apparaat thuis zetten weer.

**S** Uhu.

**P** Tis weer thuis hé.

**S** Bwa op sommige plaatsten doen ze het ook in het ziekenhuis hé.

**P** Ja? Ah.

**S** Maar ge moet ook kunnen slapen hé.

**P** Ahja. (korte stilte) Jama ze liggen daar allemaal te slape en kheb al dikwijls nekeer gezegd kzou ook neki een halfuur willen slapen mor…

**S** Tkomt ni.

**P** Tgoe ni. Tgoe niet. En als ik thuis vijf minuten naar mijnen tv kijk be..ben ik vertrokken. ’s Namiddags en hier.. Marc (*cfr. verpleegkundige op dienst*) mokt teveel laweit hé! (lacht)

*(Marc: “Men gedacht!”)*

**P** Nenieje, nenieje. nieje.. (korte stilte) Och ja (zucht)

(lange stilte)

**P** Ge kunt er niks aan veranderen hé.

**S** Mhh, nee. Der zijn weinig alternatieven hé?

**P** Ja der zijn weinig alternatieven inderdaad.

**S** Maar het scheelt wel al da de zegt da de u ni slechter voelt na dialyse enzo hé?

**P** Neen neen.

**S** En voelde zo echt.. Voor u is da krak hetzelfde voor of na?

**P** Soms ben ik wel is ver.. een beetje moe da wel. Dat wel hé. Ziede dat is twee dagen geen dialyse da voel ik wel soms.. rapper vermoeidheid hé.

**S** Na het weekend?

**P** Ja ziejet.. Of..of in de week, in de week ook want tis de maandag, de woensdag en de vrijdag..

**S** Ja?

**P** Maar dertussen kant soms zijn..

**S** Mmh.

**P** Da..Dak rapper vermoeid ben eh, dat wel.

**S** Tis beter na dialyse dan?

**P** Inderdaad, der achter ist beter ja ja.

**S** Dus ge voelt wel een beetje dat het werkt?

(lange stilte)

**P** Jaja da wel, da wel.

**S** Oké, euhmm wade..Wat veel artsen gebruiken of mensen doen een onderzoek en van die dingen, om te kijken of dat u dialyse goed werkt. Wat zij doen is de formule op basis van u bloeduitslagen hé? En dat is “Kt over v”, maar ik weet niet. Hebt u dat ooit al gehoord die term?

**P** Nieje dat hem ik nog ni gehoord, dat heb ik nog ni gehoord dade.

**S** Ook ni via u zus?

**P** Nee. (lacht)

**S** Nee.

**P** Dat heb ik nog ni gehoord.

**S** Ja tis ook ni. Tis puur een formule hé.

**P** Jaja.

**S** Oké goed. Dankuwel voor u tijd alleszins.

**P** Uhu.

**S** Zijn er nog dingen die u wilt vragen? Opmerkingen?

(korte stilte)

**P** Toch zo direct niet. Kep toch redelijk wa geantwoord. (lacht)

**S** Ja. Ja twas interessant. Kga nekeer kijken, oei.

**P** Ja dat het er op stoot of we meugen herbeginnen.

**S** (lacht) Neenee het staat erop.

**P** Staat het erop?

**S** Ja en die-

-OPNAME STOPT-

Interview patient 8

S Ik ga het interview afnemen. Ik wil vooral polsen naar uw ervaringen aan
 de dialyse, maar ik zou eerst willen beginnen met eens te luisteren hoe dat u hier terecht
 gekomen bent en wat uw verhaal een beetje is.

P Ja vanwaar moet ik beginnen?

S Het nierprobleem specifiek, van vroeger.

P Ja van vroeger ik was.. Als ik bij het leger moest gaan, ik was 18 - 19 jaar, ben ik naar “Het
 Klein Kasteeltje” geweest en toen hebben ze gezien dat ik albumine had. Albumine in het
 bloed. Dat is dan verzorgd geweest en ja dan heb ik mijn leger gedaan. Ik heb niet moeten
 gaan, ik ben afgekeurd geweest maar van toen tot mijn 60 jaar heb ik daar geen letsel van
 gehad. Ik heb heel mijn leven kunnen doen zonder problemen. Ik ben dan 60 jaar
 geworden, dan ging mijn.. hoe noemt dat daar in u bloed dat achter uit ging?

S De creatinine?

P Creatine ging achteruit en zo ben ik dan hier terecht gekomen en hebben we dat laten
 nakijken. Ze hebben dan voorgesteld, ik had maar 13 niet meer percent werking van de
 nieren. Dus hebben ze voorgesteld om dialyse te doen en dan ben ik daar mee begonnen.
 Ik heb eerst thuis peritoneale gedaan, met die zakjes, dat heb ik 5 jaar en half gedaan. Dat
 is goed meegevallen.

S Wanneer was u daar mee begonnen dan?

P Rond de 64 - 65 jaar en ik daarmee begonnen en ja die peritoneale is goed meegevallen.
 In mijn geval toch want ik deed dat zelf als we naar ergens moesten gaan. Dan nam ik die
 zakjes mee, ik deed het dak van de auto open en legde dat op mijn auto buiten. Daar deed
 ik dat met mijn auto. Ik heb daarmee geen last gehad, 5 jaar en half heb ik dat gedaan.
 Toen ben ik beginnen ontstekingen krijgen, bacteriën op de katheter en dan is de bal aan
 het rollen. De ene ontsteking achter de andere, ik moest altijd naar hier komen, antibiotica
 in die zakjes. Dat ging dan wel wat over, maar als de antibiotica uitgewerkt was dan kwam
 dat terug. Dus hebben ze dan besloten om die katheter daar uit te nemen en hebben ze mij
 nu hier een gestoken en nu hang ik zo aan de machine.

S Ja en sinds wanneer is dat dan?

P Dat is sinds februari.

S En hoe gaat het hier dan?

P Ja ik heb daar geen last van.

S Heeft u het lastig gehad om te moeten wisselen?

P Neen ik wist wat er op het spel stond. Als het ander niet meer gaat, hervallen op dit hier.
 Ja welke keuze heb je?

S Hoe bedoelt u?

P Awel ja, u vraagt hebt u daar last meegehad maar ja. Of je het nu graag doet of niet graag
 doet, je hebt toch geen keuze. Je weet wat er op het spel staat in mijn geval.

S Ja u wilt zeggen u hebt de vrijheid niet om..?

P Wel ja als er niets anders bestaat? Niet meer als dat, dan wil je dat wel aanvaarden.

S En merkt u verschil tussen dit en de spoelingen? Wat is voor u het grootste verschil?

P Ik moet zeggen ik heb er geen last van.

S En wat wilt u zeggen met geen last?

P Ik deed thuis graag die spoelingen, je word daar op afgestemd. Je weet natuurlijk als je dat
 thuis doet met al die dozen, dat wegen,… Dat was om de 3 - 4 uur, ja daar moet je u ook
 aan aanpassen. *(machine piept)* Ja nu gaat hij beginnen. Je moet u daar ook aan
 aanpassen, maar ik pas mij snel aan aan alles. Ik wist ook dat het niet meer ging.
 Dat ik hier ging op vallen.

S En heeft u een voorkeur voor 1 van de 2? Moest je mogen kiezen?

P Ik deed het andere ook graag, maar ik doe dat hier ook graag.

S Oké. Het onderzoek dat wij hier doen is vooral…

P Dus mij maakt dat niet uit of dat ik dat nu thuis deed. Thuis ben je vrijer he, hier moet je
 voor 4 uur komen liggen. Als je het uitrekent de uren dat je daar thuis mee bezig was,
 moest jij ook zitten he.

S Ja kom je met vervoer?

P Ja voorlopig vervoert mijn vrouw mij. Ik kan wel rijden hoor, maar ze brengt mij en komt mij
 halen.

S Is het ver?

P Neen, een kilometer of 13 om en weer. Van hier naar, het is nog X bij mij.

S Ja ik ben niet van hier.

P Ja dat is van X. Dus ze vervoert mij en komt mij halen, voorlopig is dat bij
 mij geen probleem.

S Nee en je hebt nog geen problemen gehad aan dialyse?

P Neen, ik kom hier 4 uur rusten. Hier kan ik wat slapen als ik wil.

S Hoe houdt u zich bezig, meestal?

P In de dag?

S Tijdens dialyse.

P Ah, hier liggen en als dat kan wat slapen.

S Ja geen boekjes, geen andere..?

P Ja als ik een krant heb, lees ik de krant of een boekje, maar dat hangt er van af hoe ik mij
 voel he. Ik krijg ook nog chemo.

S Ja dat weet ik niet.

P Ik zit met lymfo.. Aan de lymfeklieren, hoe noemt dat?

S Lymfomen?

P Dus ik moet nu nog 1 chemo hebben en dan ben ik er van af. Ik heb er al 7 gehad.
 Alles zou dan weg zijn dus dat speelt toch ook een beetje een rol. Dat ik nog een beetje..

S Ja rust.

P Rust en dat valt wel mee dat je hier 4 uur mag rusten. Thuis zet ik mij ook in de zetel als ik
 kan, dan ga ik ergens naar toe. Kan ik niet dan blijf ik liggen in mijn zetel zitten. Dat hangt
 af van dag tot dag. Hoe dat die chemo ook wat.. Versta je het?

S Ja ja die chemo dat weegt ook op u.

P Ja kwestie van hier. Ik moet komen hé? Er bestaat geen alternatief.

S Ja niet voor u in leven te houden hé? De studie die wij doen gaat vooral over de machine,
 dus niet de spoelingen, de gewone dialyse. Over de “adequacy of dialysis”, nu is de vraag
 hoe dat de patiënten dat interpreteren. Zegt dat woord u iets “adequacy of dialysis”?

Het is een Engelse term natuurlijk. Het is moeilijk om te vertalen, maar als we het letterlijk
 vertalen, komt dat neer op “adequaatheid van dialyse”. Als ik dat zou zeggen tegen u, wat
 stelt u zich daar bij voor? Of wat zou dat voor u inhouden?

P Adequaatheid van dialyse? Wat kan ik daar op zeggen? Ja ik moet ze ondergaan hé.

S En moest ik het in mensentaal zetten en ik zeg een goede dialyse? Als ik zeg wat is voor u
 een goede dialyse.

P Een goede dialyse? Ik heb hier nog geen slechte gehad hé? Juist dat de machine hier de
 helft van de tijd begint te piepen. Dat hangt af van ja, ik weet niet hoe dat komt. De ene
 keer is dat erg en de andere keer is dat minder erg.

S Ja en vindt u dat vervelend?

P Ja plezant is het nu ook weer niet. Zo dat gepiep in u oren altijd.

S En u wilt net rusten.

P Ja ze komen zij dat hier wel afzetten. Kijk ik heb maandag daar gelegen en toen heeft dat
 praktisch niet gegaan. Als ik hier lig wel, hangt dat nu af van die machine of ik weet het
 toch niet hoor. Er moet toch ergens iets zijn dat gevoeliger is.

S We zullen straks eens kijken.

P Het kan misschien, ik weet het niet.

S Dus dat zeg je van die alarmen dat merk je wel, maar hoe merk je dan of het goed of slecht
 geweest is? Los van de last, kun je daar iets…?

P Ja als ik hier buiten kom, voel ik niets. Ik voel niet dat ik aan dialyse gehangen heb. Ik heb
 nog geen problemen gehad ik ga het zo zeggen.
 Als de dialyse gedaan is, ga ik hier buiten en ja.

S Dus ik mag dan voor u stellen dat het voor u wel die tijd is die u kwijt bent maar u vult die
tijd in en u gaat weg maar u voelt verder niets? Dat is een beetje de samenvatting? En als we de vraag dan een beetje veranderen, we zeggen eerder van een goede dialyse, wat is belangrijk voor u aan dialyse?

P Belangrijk? Dat als ik toe kom ik snel aangesloten wordt. Dat duurt van dag tot dag en dat
 je hier snel buiten bent.

S Maar u zou dus ook kunnen stellen dat als je zegt van ik heb eens langer moeten wachten
 dat dat een minder goede dialyse is?

P Neen daarom niet, maar ik zeg het. Je ligt hier al 4 uur als je dan nog langer moet wachten
 om u aan te sluiten dan zit je al aan 5 uur.

S Andere dingen die u belangrijk vindt aan een dialyse? U mag op uw gemak eventjes na
 denken hoor, want het zijn ook geen vragen waar u op voorhand heb over kunnen..

P Dingen die belangrijk zijn buiten dialyse?

S Wel aan de dialyse.

P Aan dialyse? Ik hoop dat alles goed zal blijven gaan. Als het niet goed blijft gaan, kom ik in
 de problemen hé? Daarmee dat ik daar soms aan denk, ik word dit jaar 70 jaar. Ik hoop
 nog een jaar of 10 - 15 bij te krijgen, of dat ik daar wel zal raken.

S En pieker je daar veel over of denk je daar veel over?

P Ja, ik denk daar wel aan.

S Ook voor u nierproblemen al? Of vooral met de tijd dat u nieren niet werken?

P Luister, ik heb een dochter gehad en op haar 22 jaar is ze gestorven.
Ook aan nierproblemen, die had een Wilms-tumor in de nier. En ze hebben op 9 maanden
die nier daar uitgenomen, ja en dan is het spel ook begonnen. Je weet wel. Achteruit een
beetje op school en dit en dat. Ze heeft zij meer in Gent gezeten als thuis. Dan op haar 14
jaar heeft ze scoliose gekregen, dat er ook nog bij. En dan nog allemaal is ze beginnen verdikken in alles, dat ze op 22 jaar dus ook dat geërfd wat dat mijn ex had. Dat was een goitre zo een krop dat ze zeggen zo een paar keer en mijn ex haar moeder had dat ook. Ze hebben dat in X op een dag een biopsie gedaan van de nier en ook hier gekeken en een operatie aan de…?

S Aan die goitre?

P Aan de keel hé? Natuurlijk de dag nadien is ze weer gebleven en ze heeft een hartstilstand
 gekregen en ze hebben het niet gezien in Gent ’s nachts en ’s morgens was ze dood hé.

S Dat moet zwaar geweest zijn.

P Dat is nu van in ’92 dat dat gebeurd is, maar nu nog alle dagen ben je daar mee bezig.
 Omdat ik het ook aan mijn nieren heb hé, dus zij heeft geërfd wat wij alle twee hadden.
 Hier dat van mij heeft ze geërfd aan de nieren en van mijn vrouw heeft ze dat geërfd van
 de keel hé. Daarmee ben je daar altijd mee bezig, hoe gaat het met mij aflopen?
 Kijk zij heeft dat voorgehad, dan stel ik mij toch de vraag of ik niet 80 - 85 ga halen.
 Dat houdt u nu wel wat bezig.

S Ja dat kan ik mij voorstellen. Dat moet ook nog altijd lastig zijn om u kind te verliezen ook?

P Ja ja tuurlijk.

S En los daarvan is het dan nog van de nieren.

P Ja dat is alle dagen hé. Dat je daar mee bezig.. Je staat daar mee op en je gaat daar mee
 slapen.

S Hebt u dan nog andere kinderen ook?

P Ja ja ik heb er nog 2. Mijn ander komt al 13 jaar niet meer naar huis. Alle problemen vertelt hem een andere, het spel is gekomen met mijn vrouw en misschien is hij wel niet van mij. We hebben geen DNA-test gedaan want ze wou niet. Mijn andere die komen is als ze tijd hebben. Versta je, want ze moeten wel altijd ergens naar toe. Dat is het leven hé?

S Ja het is niet altijd gemakkelijk.

P Ja je moet u daar ook aan kunnen aanpassen. Als ze komen, komen ze als ze niet komen,
 komen ze niet. Ik zit nu in bed, ze weten dat ik kanker heb. Denk je dat ze daarom eens
 meer bellen of iets? Ze bellen zij niet.

S En heb je daar dan extra verdriet van?

P Nee maar dat houdt u wel wat bezig, dat je zegt je hebt dat dan, dat ze u dan eens bellen.
 Dat is toch snel gedaan om eens te vragen: “Vader hoe gaat het, is het beter?”.

S En met de dialyse dan waarschijnlijk ook dan nooit?

P Hoe bedoel je?

S Als je dan aan nierdialyse moest, hebben ze waarschijnlijk ook niet extra..

P Neen, ze weten dat allemaal hoor. Als je is komt van dit en dat, maar je kan is extra bellen
 of langskomen in de week. Maar neen want ze hebben geen tijd hé.

S En hebt u steun van iemand, een partner?

P Van mijn vrouw. Ik heb hier 10 dagen in het ziekenhuis gelegen, ik heb toen een
 peritoneale gedaan, een buikvliesontsteking. Toen heb ik hier 10 dagen gelegen en dan
 ben ik ver geweest. Dan die kanker daar nog bij, toen dacht ik van hier kom ik niet meer
 buiten. Dat ik zo flauw was, mijn bloed stond maar op 8,5 niet meer. Ja dan heb ik ver
 gezeten hoor. Kijk ik ben er weer doorgetrokken. Wat zou ik nog veel moeten verwachten
 van het leven?

S Dat zijn zware vragen.

P Ik word er nu 70, als ik nog 10 jaar bij krijg, mag ik blij zijn zeker?

S Dat is boven de gemiddelde leeftijd.

P Als ik dat hier nog 10 jaar volhoud, want je weet dat er hierna niks meer anders bestaat
 dan..

S En zijn er dingen die je hebt moeten laten voor u dialyse? Ik wil zeggen met de PD, met de
 peritoneale was u nog meer mobiel.

P Neen ik moet helemaal niets laten.

S Maar u was niemand die vroeger veel reisde?

P Ik heb 20 jaar altijd met de ‘oudjes’ gereden. Altijd naar het ziekenhuis, na dialyse,
 na bestralingen, na chemo. Dat heb ik 20 jaar gedaan.

S Als beroep?

P Neen als vrijwilliger. Heb ik dat 20 jaar gedaan. Als ik dat nu wil, kan ik dat nog. Met die chemo en dat hier bij, ben ik beter dat ik dat een jaartje of 2 rustig hou.

S En doet u nog veel thuis voor de.. U zit nu wel veel in de zetel, maar zo sociale activiteiten?
 Komt u nog eens buiten?

P Ja als het hier of daar eetfestijn is en ik voel mij goed dan gaan wij daar eten.

S Moet je hard op uw dieet letten of valt dat mee?

P Ik heb geen dieet normaal gezien.

S U heeft geen dieet?

P Maar ik pas wel wat op op mijn eten. Geen vet en geen zouten, niet extreem frieten
 beginnen eten, weet je. Ik pas zelf wat op.

S En u plast nog goed ook waarschijnlijk?

P Ja. Ik heb daar geen hinder van, ik ga waar ik wil. Als wij zeggen wij gaan morgen naar
 Lunch Garden of ergens eten, naar de Makro. Natuurlijk ik heb ook nog wat dingen bij van
 mijn evenwicht.

S Ik heb daar iets van gelezen.

P Dat is ook een ‘viezigheid’ hoor.

S Moest je van al u gezondheidsproblemen die je hebt, moest je 1 ding kunnen weghouden.
 Welk van de dingen waar je gezondheidsproblemen, welk van de dingen zou je kiezen om
 weg te doen.

P Misschien dan toch de dialyse.

S Ja?

P Met dat evenwicht pas ik mij wel aan, je loopt wel als iemand die gedronken heeft maar
 kom.

S En waarom zou je kiezen voor de dialyse weg te doen?

P Wel omdat dat toch het meest van al in beslag neemt.

S De tijd?

P De tijd ja. Dat is nu wel 4 uur. Ik vond als het nu 3 uur had geweest dat het veel beter
 geweest. Die 3 uur dat gaat snel voorbij, maar dat vierde uur is er teveel aan.

S Hebt u dat gevoel?

P Vind ik hé. Je bent wel blij dat je er bijna van af bent, ik ben hier al 3 uur. Maar dan nog een
 uur. Dan zit ik hier nog een uur.

S Het zijn die laatste stukjes dat het lastigste zijn?

P Ja dat laatste uur is er voor mij teveel aan. Kom, teveel of niet teveel als het moet dan moet
 het hé?

S Goed. Nee ik denk-

P Maar ja dat ze dat dan allemaal op 4 uur zetten. Die mensen volgen parameters en die
 zeggen voilà het is 4 uur. Maar ik ben toch van het gedacht van persoon tot persoon dat

het zou kunnen zijn dat jij maar 3 uur en half nodig hebt.
 Of iemand anders misschien maar 3 uur.

S En zou u willen dat er meer inspraak is in zo een dingen? Of zeg je ja de dokter zal wel een
 reden hebben?

P Ja als ze de parameters volgen is het 4 uur dan is het 4 uur. Wie dat dat op papier gezet
 heeft, weet ik niet waarom hij daar 4 uur opgezet heeft.
 Het zal wel een reden hebben zeker? Maar dat dat dan voor iedereen 4 uur is, denk ik wel
 dat er iemand blij zou zijn met 3 uur en half zo volgens zijn toestand.

S En de term “Kt/V” kent u dat? Dat is ook gewoon een medische term, daar wordt de tijd
 mee berekend. Nee? Oké ik denkt dat het voor mij een leerrijk moment en gesprek was
 met u. En ik hoop dat allemaal goed blijft gaan aan de dialyse.

P Het is mijn laatste kans hé? Als er hier problemen zijn, dat moet je in u hoofd steken.

S Als dit niet meer zou werken, bedoel je?

P Als dit niet meer gaat, is het tussen de 4 planken hé? Als je het zo ziet. En daar ben je toch
 wel een beetje mee bezig.

S *** stop opname ***

Interview Patient 9

S Goed? Dus ik zou eigenlijk gewoon willen beginnen met eerst een keer te luisteren beetje
 naar uw verhaal, hoe u aan dialyse terechtgekomen bent, wat er met uw nieren aan de
 hand is, hoe lang, …?

P Ja ze hebben dat eigenlijk ontdekt toen dat ik zwanger was van mijn zoon. Toen was ik 19
 jaar dus ja ik word er nu 38 dus dat is al eventjes geleden. Ik had eigenlijk geen klachten,
 ze hebben dat gewoon gezien tijdens een bezoekje aan de gynaecoloog. Op een bepaald
 moment heb je het routine onderzoek van u plasje dat je moet binnenbrengen en via dat is
 dat allemaal eigenlijk een beetje in gang gekomen.

S En sinds wanneer wordt u dan gevolgd bij de nefroloog enzo? Vanaf dan?

P Vanaf dan ja.

S En dialyse sinds wanneer doet u dat?

P Nu 2 jaar en half.

S Altijd hemodialyse of..?

P Ja altijd hemodialyse ja. Ik heb dat bewust gekozen omdat ja voor mijn kinderen. Ik heb
 thuisdialyse ook eventjes overwogen hoor, maar mijn kinderen zijn daar te gevoelig voor en
 ik wil ze niet teveel confronteren met… Nu doe ik het hé, ik bedoel ik kom naar hier en
 niemand heeft daar last van. Enkel ik dan, maar thuis bedoel ik dan, thuis worden ze daar
 zo niet mee geconfronteerd.

S En uw kinderen hoe oud zijn die?

P Mijn zoon wordt 18 en mijn dochter wordt 11.

S Oké en wonen ze nog thuis?

P Jaja.

S Ja maar de ene gaat waarschijnlijk naar de unief of studeert af?

P Nog niet, nog niet. Hij is eens blijven zitten dus hij zit nog maar het vijfde.

S Ja oké. En werkt u nog?

P Nu doe ik terug een administratieve job, eerst zat ik in de zorgsector, maar een keer de
 dialyse gestart, ben ik daar mee gestopt want dat kon ik niet combineren. Ik had ook niet
 echt een baas om tegen te zeggen van een beetje weg, een beetje soepeler, een beetje
 aangepast werk. Dus het was een beetje alles of niets dus daarom heb ik twee jaar niet
 gewerkt en ik ging regelmatig op bezoek bij de arbeidsgeneesheer. Die merkte dat dat dan
 aan mij dat dat niet echt mijn goesting was dat ik zo thuis zat. Dus die zei van waarom
 neem je geen medisch ontslag en ga je iets anders zoeken dat je wel kan combineren?
 Dan heb ik dat ook gedaan, ben ik beginnen zoeken en mijn schoonbroer die heeft een
 eigen bedrijf en die raakte niet meer door zijn administratie zelf. Nu doe ik dus de
 administratie en ik breng dat mee naar hier ook.

S Oké en als u gestart bent met de dialyse, de maanden daarvoor, wist u op voorhand een
 beetje wat dat allemaal inhield? Zoveel processen dat u daar naar toe leiden, hoe dat u dat
 ervaren hebt?

P Ja. Ik ben veel informatie zelf beginnen opzoeken, dat doe je ook automatisch omdat je
 denkt van ja waar sta ik voor. Ik heb daar eigenlijk ook niet super veel bij stilgestaan ook
 niet hoor. Ik heb het gewoon op mij laten afkomen.

S En in het begin, is dat vlot gegaan of hoe waren die eerste dialyses, die eerste maanden?
 Kan u zich daar nog iets van herinneren?

P Ik voelde mij beter. Net voor de dialyse heb ik echt een paar slechte maanden gehad, was
 het elke dag die misselijkheid dat ik had, mij niet goed voelen en geen energie meer. Dat
 was er aan de hand en ik ben dialyse gestart en ik voelde wel een verschil terug. Ik voelde
 mij terug een beetje beter.

S U hebt altijd enkel een fistel gehad of ook een katheter?

P Nee, ik heb ook een katheter gehad in het begin. Persoonlijk ben ik daar blij voor dat ik
 daar vanaf ben, dat vond ik een beetje de vrouwelijkheid… De vrouwelijkheid van een
 katheter..

S Van de katheter?

P Ja dat tast u vrouwelijkheid een beetje aan. Waarom? Ja je zit hier met een buisje dat je
 ziet lopen en dan komt er hier nog een tube uit voor een heel groot stuk. Dat moet dan elke
 keer proper afgewonden worden om geen ontstekingen te krijgen, dus je zit hier met een
 open wondje. Geen bad, geen zwemmen, ook ga je je automatisch afschermen dat de
 mensen dat niet zien. Met dit had ik dat ook in het begin hoor.

S Ja ik ging het juist vragen.

P Ja als dat begon te zwellen, die aders beginnen daar dan zo op te liggen. Dan dacht ik van
 “Oei ik ga mij een T-shirt aan doen maar toch met een pulletje over, zodat de mensen dat
 niet zien”. In het begin deed ik dat ook, nu begint dat te minderen. Nu denk ik van ja kijk
 wie dat het niet kan zien, moet er maar naast kijken.

S Vertelt u er veel over tegen mensen of beginnen daar vaak mensen over?

P Ja ze zeggen toch altijd: “Dat doet toch pijn in u arm?”. Dat is het eerste dat ze vragen,
 maar nee ik heb daar geen pijn in. Je ziet ook wel als ik bij de bakker ga, over 14 dagen
 was ik bij de Panos. Het was super warm dus ik had een T-shirt aan en die juffrouw achter
 de toog was in shock. Dat zag ik wel. Die kon niet stoppen met naar mijn arm te kijken. Ik
 dacht van: “Oké ik snap het, maar je overdrijft”.

S Ja dus je merkt wel duidelijk verschil met eigelijk zo…

P Ja je ziet de mensen zo denken van wat zou daar achter zitten.

S Ja wat is dit?

P Ja wat is dit. In het begin dat ik van ze gaan hier allemaal denken dat ik aan de drugs zit.
 Dat dat sporen zijn van naalden, want het zijn ook duidelijk sporen van naalden en dan was
 ik aan het denken dat het wel erg zou zijn, moesten mensen dat over mij denken. Nu heb ik
 zo iets van ja dat ze denken wat ze willen. In het begin trok ik het mij wel allemaal aan, nu
 niet meer dat is gedaan.

S Ja dat zijn nu wel zo van die dingen dat interessant zijn om te weten. Omdat iedereen dat
 proces, ja dat is zeer individueel. Het onderzoek dat wij concreet doen is gebaseerd op een
 studie waarbij ze gekeken hebben naar alle zaken die patiënten in de literatuur
 gerapporteerd hebben over dialyse. En dan hebben ze dat nagedaan bij patiënten, wat
 patiënten allemaal belangrijk vinden uit die lijst. 1 van de zaken die de patiënt belangrijk
 leek te vinden was “adequacy of dialysis”. Het moeilijkste van dat onderzoek is dat dat een
 Engelse term is om mee te beginnen. Bij oudere patiënten is die Engelse kennis soms wat
 minder en omdat dan te vertalen. Maar ik zou eerst willen luisteren of die term “adequacy
 of dialysis” of dat u zich daar iets kan bij voorstellen?

P Niet direct? Ik ben aan het nadenken, mijn Engels is niet zo slecht maar..

S En “adequacy” op zich dat woord? Zou dat u iets zeggen? Als je het letterlijk vertaald, met
 nog wat meer moeilijke termen is dat “adequaatheid van dialyse”. Ik weet niet of u dat wat
 meer zegt?

P Ik zou denken aan het nut van de dialyse. In die zin zou ik beginnen denken.

S Ja, kunt u daar nog wat meer over vertellen als iemand zo zegt wat vind jij de adequaatheid
 van dialyse? Hoe zou u dat bekijken of hoe zou u dat evalueren of u dialyse adequaat is of
 niet?

P In mijn geval denkt ik dat dat wel zo is. Ik bedoel ik kan een vrij, tussen haakjes, leven
 leiden. In sommige gevallen dat ik rondom mij zie, dan denk ik dat dat niet altijd het geval
 is. Ieder voor zich he, maar er zijn toch bepaalde dingen die ik voor mezelf uitgemaakt heb.
 Tot zover wil ik gaan en meer niet.

S Kun je daar wat meer over vertellen?

P Ja, moest ik nu elke dag ziek zijn en heel slecht reageren op die dialyse dan zou ik liever
 hebben dat het stopt. Als ik echt afhankelijk zou moeten zijn van iedereen rondom u en u
 niet meer zou kunnen leven zoals je zelf wilt. Dan zou dat voor mij het gevecht niet meer
 waard zijn.

S Als we dan de term zouden pakken in plaats van adequaatheid van dialyse een goeie
 dialyse? Is dat voor u dan wat gelijkaardig, een beetje vergelijkbaar?
 Wat is voor u een goede dialyse?

P Ja, vergelijkbaar hoe bedoeld?

S Die 2 termen.

P Het nut. Dat vind ik een moeilijke. Ja een goede dialyse. Vergelijkbaar? Ergens misschien
 wel.

S En als ik u vraag wat is voor u een goede dialyse?

P Ja wat is een goede dialyse? Vind ik ook een moeilijke.

S Je mag gerust eventjes nadenken, het is inderdaad geen gemakkelijke vraag.

P Ja een goede dialyse, ik denk dat een goeie dialyse vooral inhoudt dat de zuivering op de
 juiste manier en goed gebeurd. Uiteindelijk is het maar een percentage van 10% heb ik mij
 toch steeds laten vertellen, als je daar over nadenkt dan weet je dat er heel veel
 afvalstoffen niet gaan rondzweven.

S En merkt u dat of een dialyse goed geweest is of niet?

P Nee, niet elke dag is hetzelfde. Niet elke dialysedag is hetzelfde, maar ik denk dat dat ook
 afhankelijk is van hoe je u voelt. In het hoofd ook. Voor mij is dat iets, ik vind dat je veel
 bereikt met hoe je er mee omgaat. Je hebt twee keuzes, ofwel laat je u gaan en dat gaat u
 niet helpen ofwel vecht je ervoor.

S Bent u in de uitwerking voor eventueel een transplant?

P Ja.

S Ja stel nu. Staat u op de lijst?

P Ja.

S Dus u wacht nu eigenlijk op de telefoon dat je naar X gaat en dat de dialyse kan
 stoppen. Moest u nu niet op de transplantlijst staan, hoe zou dat voor u zijn? Kunt u zich
 dat enigszins inbeelden? Zou dat voor u de ervaring veranderen?

P Dat zou heel veel veranderen dat is een feit. Ik zou misschien een andere vorm van dialyse
 kiezen om te beginnen, om toch niet elke keer 3 keer in de week naar hier te moeten
 komen. Maar het zou een tussenweg moeten zijn tussen er thuis niet te veel mee
 geconfronteerd te worden en dan naar de kinderen toe. Ook die buikspoelingen dat is 4
 keer op een dag, dat is 4 keer op een dag dat je daar mee bezig bent. Dat je uzelf moet
 dialyseren. 4 keer per dag. Neen daar kan ik mijzelf echt niet in vinden.

S Ik heb zo een beetje de indruk dat u dat echt probeert te scheiden.

P Ja ik scheid dat echt.

S U mag mij corrigeren indien ik verkeerd ben hoor.

P Neen bij mij is dat echt een scheiding. Ik praat daar thuis ook niet zoveel over, tenzij dat het
 echt noodzakelijk is. Of als ik mij eens echt niet goed voel dan zal ik dat eens vernoemen
 maar ik praat daar echt niet veel over.

S Moest u nu de dialyse kunnen verbeteren, wat zou u in een soort van magische wereld
 willen, maar u kan niet wensen dat u geen dialyse meer heeft. Iets aan het hele proces
 veranderen, 1 ding dat u graag zou aanpassen.

P Ik zou vooral… Dat is ook geen gemakkelijke.

S Inderdaad ook omdat de opties eindeloos zijn.

P Ja.

S U mag ook enkele ideeën geven, dat of dat stoort mij het meest moest ik dat kunnen
 aanpassen.

P Ik vind het echt moeilijk om iets…

S Iets dat veel mensen bijvoorbeeld aanhalen is de tijd.

P Ja ik heb daar niet zoveel last van want ik ben altijd aan het werken en ik heb te weinig tijd.
 Uiteindelijk zit ik hier wel op mijn gemak.

S Je hebt geen storingen.

P Ja, echt op mijn gemak dat wil ik nu ook niet zeggen maar soms denk ik wel van: “Amai is
 dat hier al tijd? Is mijn tijd hier al op?”.

S Je kan u tijd hier nuttig gebruiken?

P Ja ik denk dat dan wel een groot verschil is met als je dat niet kunt. Dan is het wel triestig.

S Ja maar het gaat over uzelf hé?

P Ja, ja moest ik iets zou mogen veranderen, ik zou een manier zoeken om een betere
 zuivering te krijgen van het bloed dat ik toch een keer meer zou mogen zondigen qua
 voeding. Dan zou ik eerder voor zoiets gaan.

S Let u zeer hard op uw dieet?

P Ja dat wel ja.

S En lukt dat? Hoe is dat geweest voor die aanpassing?

P Dat is niet altijd gemakkelijk, ik bedoel dat gebeurd wel dat ik een hele andere menu maak
 voor mij alleen omdat ik denk van oké.. Ik ga u een voorbeeld geven: macaroni met hesp
 en kaas in de oven, liefst met heel veel kaas. Dat is allemaal heel zout, die kaassaus
 gemaakt met melk ook een vergif. Allemaal dingen dat ik heel graag eet en drink. Dus ja
 dan maak ik voor mezelf dan een andere pasta met tomatensaus op mijn manier. Zo weinig
 mogelijk vochthoudende dingen. Tomaten zijn niet goed voor als je vocht ophoudt, maar in
 mijn geval kan dat er nog door.

S Ja je hebt wat meer marge om te drinken wel dan?

P Ja.

S Nog een laatste vraag. Het is heel interessant. Dus het concept van wat is een goede
 dialyse voor u, is dat dan effectief dingen die met de machine te maken hebben? Minder de
 dingen de dingen die rondom de dialyse gebeuren?

P Neen dat is ook belangrijk rondom de dialyse. De zorg naar de mensen toe vind ik ook heel
 belangrijk.

S Wel maar dat is iets dat je belangrijk vindt aan de dialyse, maar als je de term ‘goede
 dialyse’ op zich. Valt dat daar ook onder of is het de zorg en de interactie dat je allemaal
 belangrijk vindt bij dialyse of dat een deel van het woord goeie dialyse? Het is gewoon
 persoonlijk hoor, er is geen juist of goed antwoord.

P Een goede dialyse is voor mij een dialyse waar dat jij als persoon goed op reageert en
 waarna je dan eigenlijk goede resultaten hebt.

S En de dingen kunt doen die je wilt doen.

P En de dingen doen die je wilt doen tot zolang dat het nodig is en dan ja wat er dan bijkomt
 is ook belangrijk.

S Ja maar het is een ander concept?

P Ja.

S En zijn er, om te weten of u dialyse goed is, zijn er dingen die je zelf in de gaten houdt of?
 Hoe weet je dat u dialyse goed is? Je zegt ik reageer er goed op, maar hoe weet je dat?
 Wat gebruik je?

P Ik veronderstel dat, maar ik weet wel wat dat allemaal wilt zeggen hoor. Het enigste waar
 dat ik op let is dat er maar 50 van het vocht afgaat en dat dat juist ingesteld is. Dat weet ik,
 maar er zijn nog nooit echt… En ja dat ijzer, maar er zijn nog nooit echt problemen
 geweest.

S Oké en als de artsen langskomen om bloedresultaten te bespreken, is dat iets dat je zo ook
 gebruikt van de machine werkt of meer van mijn dieet werkt?

P Soms kan dat wel eens gebeuren dat ik een te hoge kalium had, maar dan denk ik meestal
 niet aan de machine maar denk ik aan mijzelf. Van “Ja oké ik heb in het weekend dat en
 dat gedaan en dat zal er misschien toch wel een beetje over geweest zijn”.

S Ja maar je vindt het wel eens interessant om zo eens te horen en te bespreken?

P Ja ik weet dat wel graag. Op regelmatige basis. Dokter doet dat, dan komt
 ze rond en op regelmatige basis zegt ze dan van je bent goed bezig of je moet minderen
 met dit of dat. Ik vind dat wel tof dat ze dat doet want anders heb je geen idee. Ben ik nu
 goed bezig of ben ik nu niet goed bezig?

S Omdat de dieetfouten er langzaamaan er in sluipen en je hebt geen feedback?

P Ja voilà.

S Oké goed, ik denk dat ik alles gevraagd heb wat ik wou vragen. Ik vond het zeer
 interessant, maar het is bij ons ook meestal …

*** stop opname ***

Interview Patiënt 10

S Zo nen telefoon dak kan voor alles dienen tegenwoordig.

P Ahjoet. Joemoe, ons... Ons maske heed oek azu ienen (gerommel) opstoan.
En foto’s pakken en …

S Ja en sturen [P: en weet ik allemaal ewa] en mails.

P En computersdingen.

S Ja en agenda’s.

P Ja en agenda’s.

S Euhmm.. Nu enkel, ik ben aant afvragen of dak mij ook aan de kant van de tafel zou zetten.
 Mag ik da tafelke den andere kant zetten?

P Ma bajoat. Doet ma hoe da ge da geiren [S: Tis nog..] hed.

S Tis nog een beetje installeren hé.

P Ja.

(lange stilte)

S Ik ga ook.

P Voor u gemak is hé.

S Awel het moe voor u ook passen hé. Dus ni verschieten ik ga ook dingen opschrijven. Dat is eigenlijk gewoon om een beetje zo..

S Als je dingen vertelt en ik kan u ni onderbreken, kan ik achteraf .. Daar nog een keer opnieuw terugkomen hé.

P Ja.

S Goe. We gaan dat hier een beetje dichter zetten.

(gerommel en gekraak)

S Ja. Dus de studie gaat zo’n beetje of t’interview hé, da gaat over hoe da mensen da..be.. doen aan dialyse, wat ge zelf vindt van dialyse. Maar ik zou eerst ne keer willen luisteren wat dat eigenlijk het probleem me u nieren was en hoe da ge aan de dialyse beland zijt enzo.

P Das al.. Hoe kind.. Dat is verzeid al zeker al mier als zeven of acht joer dakik [S: Ja] Mo.. Ma kem t’ierste niere das al neig langk geleden ze. Da kan al twintig joer zijn, mijn niere kwijt .

S Ja.

P Die was slecht allez zemme ze ten moeten uitnemen en zebben ten geopereerd. Mo kem altijd nog goed geweest, kem nog kunnen goen werken en al. [S: Uhu] Enne.. Enneuhh en dan ben ik hier dan beginnen te verslechten hé. Ge weet mijne rug zie en en nog weet ik allemaal watten. En ten ben ik allie gekommen en ten moest ik na de dialyse kommen hé.. Moest ik.. En azuu, jamoe kem er nog langk mee.. Kem er nog veel mee gesukkeld ze, emmik. Altijd me mijne rugpijn hé. Ooehh da was.. [S: Uhu] vried ze. En kging ten werken, gingk ik, en kmoest ten altijd die liters woeter droagen en da was teveel hé. [S: Ja] Die liters droagen hé jong. Da.. Da kost ni zijn hé. [S: Ja] Kem vant leven afgezien zeh, en ten hebben ze het geopereerd. Dan benk doa in Oiljst doe geweest. [S: ja] int ASZ hé. [S: Ja] Kem ten nog langk.. Allez dernoa hemmik nog goe geweest ze, mobajoak. Nog kunnen goen werken dernoe [S: Ja] emmik.

S En waarde… werd ge al lang opgevolgd?

P Bajoak. Aiaiai. Da was euh.. Ja in’t begin moest ik ten altijd noar Oiljst goen na de controle.

S Ja.

P Bekan alle moanden hé! [S: Uhu] Alle moanden moest ik goen.. Awel ja twas ten altijd goed. Op den duur gingk ik ten ni mier. Ten ben ik na allie geland,.. [S: Ja] bennik. En dingen ad ten gezeit “Jama u ander nier werkt oek ni meer zooo… perfect.”

S Ja.

P We zijn dan…Op den duur zei ze om een beetje te verhelpen. [S: Ja] Dermee hemme ze da gepro.. Das al joeren hé kind dakik [S: Ja] hier komme.

S Ma ge hebt het zo allez ge wist dat het er aankwam, twas ni super plots?

P Nenein

[*praten samen*] [S: Tis gegaan opt gemak] [ P: zo opt gemak jaja.. Opt gemak.]

S En hebde gij ne fistel of ne katheter?

(Lange stilte)

P Hieje.

S Ne euhh.. ne Katheter.

P Ja.

S Ja, oké. En hoe stelde het? Aan dialyse.

P Neig goed. Neig goed. Vroeger wast in mijn arm hé. [S: Ja] Ma da euh.. Da was ni goed.

S En hoe komt het?

P Awel ik hem ten nekeer deur een duur volledig uitgebloed. Ma neig gebloed ze! ’T Spietjigen tot in den blafon’ en de vloere. Twas gralijk en ik kost da ni stelpen. Ten bennik sebiet na de kliniek gekommen, ten hemme ze’t anders gedoen hé. [S: Ja] Emme ze da toe en hier ten gedoen hé.

S Ja en wete nog als ge zo begon me dialyse hoe dat da was voor u? Zo die.. In’t begin, zoveel jaar terug?

P Mo.. Kannekik doe van.. Ja dade da is wel woe als ge na de dialyse komt, aje er ten afkomt ternoe, dienen dag, zijde wel ni veel weird. Da ge moeg zijt. [S: uhu] Ma feitelijk op de dialyse zelf niet. De.. Dei..Doe ebde da ni dat ni goe goet, ma als ge thuis komt en ge wilt iet doen, da goat niet. [S: Neh] En da goe noa nog niet.

S En das gewoon altijd het zelfde geweest.

P Joet . Ge wordt ten moeg hé. Moe.

S Ja.

P Ten doennekik ni veel . Op dienen dag gelijk as vandoag kommik nu thuis van de namiddag en ik zal ni veel doen. Veel rusten hé. Mo.. Mo mergen [S: Uhu] bennik ne man hé.

S Ja.

P Ten bennik weer

S En wat doede dan zo allemaal?

P Awel nog mijn huishouden hé volledig. [S: ja] Kuisen, eten maken, strijken, wasken,…
Wa ge kunt hé. Wa da ge allemaal [S: Ja] moet doen hé. [S: Ja] Tis der veel te doen hé.

S Ja en hebde euuh..ne man nog enzo?

P Joaaa.. ma joaak en een masken oek.

S En kinderen?

P Een masken. [S: Ah ja] Een meisjen.

S (Lacht) Ja soms is het dialect hé!

P Jaja dialect toet.

S Ma ge moogt… Babbelt maar ze, maar euhh..

P Jajaja (korte stilte) **** (onverstaanbaar stil) Ons Liesbeth is nog thuis, ma ze is wel gehandicapt hé. [S: Ahja] Ze heeft euhhh… een doof.. wa zeggen ze doof.. [S: Uhu]
Ze hoort niet en ze.. ze kan ni spreken of niks hé. Ma tgoat goed eur dingen. Zis nog ba ons thuis en ze trekt goed euren plan.

S En heeft de dialyse daarop veel veranderd? Zo op u gezinstoestand of euh..?

P Naaaiinntt.. Da blijft da altijd zelfde hé. (stil gemompel) Mijne man moet hij wel een beetje mier inspringen nog. Een beetje doagen dakik ten ni kan, moeten hij mo ten ewa helpen. En Lieschbeth helpt mij oek veel. [S: ja] De doagen dak allie kom, moe ze zij mij veel helpen hé. Doe ze zij veel mijn werk. (lange pauze) En azu.. Hmm..

S Ja, ma tis wel nen tijd zegt.

P Mojoet… Mojoet. Groh ja hoe langk dakik doemee al… Het kan al misschien 15 joer zijn dakik die nieren kwijt ben. Of nog mier… Want.. Da stoet hier allemoal te boeken te doen mo. Kwetekik da ni vanboaiten hé. [S: Neh.] Da weet ik.

S En euhh hebde het soms moeilijk gehad met het feit da ge naar de dialyse moest?

P (Korte stilte) Awel in het begin ik kom ni geiren hé en hij had gezegd “Ah ja mo wa gaat da weer zijn en zo langk..” [S: Ja] Das wel een beetje ‘t dingen dat wel lang duurt hé. [S: Ja] Het duurt wel langk hé. Joa bij mij ist ten 4 uur en half. Das nog langer hé. (korte stilte) Mo het duurt wel langk hé. Moja soms goeget nog ze. (Lange stilte) Ik.. Ik verveel ma ni allez.

S En was ..

P Kem .. mijnen babbel hé (lacht).

S Met de buurvrouw?

P Joa joa…

S Zit ge meestal naast elkaar?

P Wij liggen altijd tegenien.

S Ja, oké. Ge hebt er deugd van voor een keer een klapke [P: ma bojoak] te kunnen doen?

P Ne keer een babbelke te sloen hé.

S Ja en is het al lang da je die 4 uur en half moet doen dan nu?

P Wat datte?

S Is dat al lang da jullie die-..

P Joag, van int begin.

S Vanaf het begin 4 uur en half?

P Van int begin.. (korte stilte) Van int begin..

S Ge hebt nooit 4 uur gekregen?

P Toet, gieeell int begin wel. Tierst [S: Ja] wast begost me 3 uren en ten een beetje mier. Altijd een beetje mier me ne keer hé. En ten wast 4 uur en half…

S Ja.. En hoe was da voor u om telkens zo nog .. nog langer te moeten? … Maakte da veel verschil?

P Mabajoat tes ma een halfuurke hé. Ja se.. Da goet ten nog. Ma int begin hemme ze wel gesukkeld ze me mij. Hie, veu te steken hé. [S: Ja] Doe hemme ze nog veel, veel mee gesukkeld ze int begin. En ze vonden ten.. dienen dingen ni hé. Dienen ader.

S Dienen fistel.

P Ja ja, en ze kregen geen bloed hé. Ze hemme nog langk gesukkeld dermee, mo kben content da het hie is noe hé.

S Van die katheter?

P Ja, hie doet da geen ziej of niks hé, moe doe died da ziej hé!

S Ahja ma alst geen pijn doet, ist veel makkelijker. [P: Joet] En ejje dan al lang een katheter? Of is dat ietske.. ?

P Zemme da dernoe gedoen, .. peis ik. Een joer of twie noa misschien? [S: Ja] Alst twie joar is…

S Ma ge zijt contenter met de katheter?

P Joak kem liever dade.

S Ge zou gene fistel meer willen? ….

(korte stilte)

S Nee (lacht) Kziet.

P Nieje zeh. Ma ja jong. Da bloed doe zitten uit te spieten. Ze moeten rap zijn ze ten. Om.. ja.. Om… en ten na “Spoed” te komme en sebiet ten moete bloed geven. Ja kwast er kweet niet hoeveel kwijt hé jong.

S Ja als ge het niet kunt stoppen, .. [P: Brrrrrr…..] en het spuit eruit.

P Ze kosten het ni stoppen hé. [S: Ja] (patiënt jammert bijna geluidloos) Ze kosten ‘tni stoppen..
(korte stilte) En ten me den ambulance ten na allier kommen. Ma da was ten al (patiënt mompelt)

S Allez maar nu euh.. met de katheter zijde content?

P Jo joa joaa kben der content van.

S De-…. (Patiënt valt plots in) Moe doemee ist nimier proberen. Nie nie niej.

S Is dat voor u een groot, da’s voor u een groot verschil hé zo nu -.. [P: Ja ja]

- Praten tegelijkertijd – S: Ne katheter of nen euh.. –P: Ja das een groot verschil.

S Maakt dat euh.. Maakt da da voor u ook de.. de dialyse aangenamer is da je ne katheter hebt?

P (korte stilte) Mo bo.. Da’s allemoel tzelfde hé. Da blijft zelfde.

S Tis enkel den prik?

P Ja.

S Ja.

P Aahnja da dingen hé.

S (haalt diep adem) De studie gaat bij ons wa over de “Adequacy of dialysis”. Nu dat is een Engels woord hé en das … (patiënt mompelt iets) Voilà en das zo ne medische term. Als ik het wil letterlijk vertalen.. hé?.. Dan komde uit op adequaatheid van dialyse. Maar das ook nog altijd een moeilijk woord. Euhm… Als we het.. N.. Nog wa veranderen is het eigenlijk een goeie dialyse. Dus de vraag is een beetje: “Was voor u een goeie dialyse? Wanneer vinde gij da.. dat.. een goe.. een dialyse een goeie dialyse is geweest?”

P (Korte stilte) Bo.. Alst een goeien en alst azu joej. Ten gagget goed. En emmek geiren, als ze reclammeren hemmik het ni geiren. (lacht)

S En wie..?

P De dokter kan pertang neki reclameren hé. [S: Ja?] Te veel kalium, te veel fosfor,.. [S: Ja], teveel dade.. (lacht) Deze hier ni, dieje da na ieren doet hé, maar die euh..

S Is ze strenger?

P Joe joes.

S En maakt dat dan uit wie dat er u, wie dat er langskomt voor u of dade zo zegt van…?

P Oeh na neiggg. Das allemoel tzelfde.

(lachen samen)

S Maar ge hebt graag gewoon .. mensen rond u? [P: Ja jong] ja ja.. (korte pauze)
En van de dialyse zelf, zo van het toestel of andere dingen of.. ? Kunde daar andere..? Als ge zo ne keer alles bekijkt van een dialyse wa zou er voor u.., wa is er allemaal belangrijk aan een dialyse om te maken .. dat goe is? Ge zegt de mensen zijn voor u wreed belangrijk merk ik?

P Ja ja joat..

S Wa nog? Ge moogt op u gemak nadenken ze, ik ga u wa tijd geven.

P Kweet kik niet. da goa nog allemoel redelijk goed. Dat is just dienen dingen, dat langk duurt hé.

S Dat lang duurt? Ja.

P [fluistert: Dat langk duurt hé]

S En ist er voor u een punt dade zegt van: “Dan zou ik het ni meer doen dialyse”? Bijvoorbeeld moest het zes uur worden -…

P Aaahn ja ma ge kunt ni zonder hé! Ge moet het hemmen hé, want tschijnt da als ge het nieje komt [S: Ja?] dan kunde op twie – drei doagen er [S: Ja] vantussen kunt zijn, daje vergeven werd. Nie niej da dat is voor mijn leven hé.

S Ja, maar stel nu ze zeggen van: “Kijk vanaf nu moete zes uur komen met dialyse”, bijvoorbeeld.

P Nieje da zouk ni oenkunnen. Nieje das te veel. Want kem nog geweest.. neki da heeft ma iene kier geweest hé. Dak nekier drei doagen achterien moest kommen. En das te veel. Da kan ik ni oen. Da kost ik ni oen. Da was te veel. Kweni mi ze om wa reden da da was dakik drei doagen achterien moest kommen .. (haalt diep adem) en da was te veel. Da kost ik ni oen.

S En kunde wa meer vertellen over: “Da was te veel” ? Was dat fysiek, was dat mentaal?

P Awel

S Wa da…

P Moeij. Tot en met moeij. Gien corroge van niks tein. [S: ja] Tuut, tuut ma da heeft mo iene kier. Kweni m.. Ik k.. k…kan ni mie zeggen ze waarom. Wa reden da da was da kik daarveur neki drei kieren achterien. Da weet ik ni mier. Mo da..da..das teveel. [S: Ja]
(fluistert) drei doagen achterien.

S Duss-

P Om wel te zijn moet er da da nen dag tusken. [S: Ja] Gelijk alst nuij ist goed. [S: Ja] Tes goed.

S (korte stilte) Dus.. Moest dialyse elken dag zijn, dan zou je het ni kunnen?

P Kzou het ni goed kunnen. Da kan ik ni aan. [S: Nee] Das te veel.

S Ja, en moest de dialyse altijd lang..langer zijn: zes uur of zeven uur?

P (blaast) Nee zeh! Dan ni zeh. Da..Da kan ni zeh. Zes uren. Goed jongk. Das te vele zeh.

S Neh. Ja normaal doen ze da ni hé. Ma tis gewoon puur, zo .., moest het zo zijn, wa daje zou doen?

P Ja ja khad da oek al gepeist. Ahja alsket goa zetten moet hem doar stoen, dan moet ge der stoen mor.

S Da zou u ongelukkig maken –

P Kzou der toch gien, nieje da zouk ni neig vo te zijn.

S En omwille van den tijd of omwille van da je denkt dat die euh.. achteraf meer last gaat hebben? Waarom zou die zes uur dan.. zoveel lastiger zijn dan de vier uur en half?

P Mja.. Kweet kik dat oek ni hé. Da weet ik.. da weet ik na oek ni.. Das allemaal nuut he.

S Omdat te lang is gewoonweg? Da je zegt van ik wil ni zo lang hier int ziekenhuis gerust zijn?

P Awel nee, mo da is langk, das nuij al langk [S: Ja hé] Die vier uur en half. Ahja, das nogal langk hé. (lange stilte) Ma tis toch wel vermoeiend ze dernoe. Ik ben der toch moeij af. Gelijk vandaag oek, van de namiddag, ben ni veel weird ze. Ge moet ma ni vroagen: “Doet neki mijne strijk of .. of doet ne keer af, of ik zal neki kuisken van den achternoene”. Nieniejniejniej doe goen ik ni oen beginnen. [S: Nee] Want tis just mijn eten dak zal maken en tzal gedoen zijn.

S Hebde betere of slechtere dagen wat da betreft van de vermoeidheid?

P Nee das altijd tzelde.

S Ja.

(lange stilte)

P Das altijd tzelde hé.

S En ge vertelde dadeuummm.. sommige dokters nekeer kunnen over de kalium en de fosfor bezig zijn (patiënt lacht) Is da voor u, wa heeft, da heeft waarschijnlijk, ma wat heeft da voor u te maken als ze klagen en over u fosfor beginnen? Wa wilt da dan.. Wa betekent dat dan voor u?

P Awel ja tis miskien wel veu iet.. tzal wel miskien wel gelijk hemmen wa da ze zeit.

S Ja.

P Maja das allemaal zo simpel ni hé. Ge kunt er toch de brokken op de stukken ni af naaien. (lacht)

S Ja nee nee. Dus het heeft te maken met het dieet dan hé?

P Ja ja joet [S: Ja] Da heeft altijd te moaken me dieet hé.

S Is da voor u-

P Of te veel geten of te veel gedroenken of te veel … (lacht)

S En zijde.., waarde dan vroeger nen Bourgondiër?

P Hoe wilde zeggen? Euh.. Ne goeien eter?

S Ja.

P Baja vroeger joak, ma noa zoviel ni mier hé. Ooeehh ik eette veel veel minder. Vele minder, kemmekik azoe gienen appeteit ni mieje. Veel minder zegt.

S En was da-, was dat euhmm.. moeilijk om zo u dieet aan te passen enzo dan?

P Moh da, da goet. Da komt vantzelfs. Da komt vantzelfs… kommet. Ja vaneigens alst a is smokt of iet en ge goat dan is neki goed eten en dan wel neki.., meude oek ni doen hé.
[S: Ja] En alst a is neki goe smokt.

(korte stilte)

S En gade soms op restaurant of van die dingen?

P Nee, nonoa nieje. Tis van zen leven niet.

S Nooit gedaan?

P Nieje nuut ni gedoen. [S: Nee] Wel naar eetdingen hé ge weet noa bijvoorbeeld een eetdingen van dit of een eetdingen van dade.

S Van de verenigingen enzo van die dingen?

P Ja van die dingen. [S: Ja] En doe goen we no toe hé. Op restaurant nuut. Jamais.

S En zijn dat dan van die verenigingen waar ge zelf in zit of dade kent, of… ?

P Jamajamajaak wemmen er wel twie draa.

S Hebde veel zo … sociale conta- euh.. sociaal leven zo na de dialyse? (praten samen)
[P: jamaja jajaak ja-]

P Awel joejoe der komt nog wel bezoek en..

S En kunde een keer wa-.. der over vertellen?

P Over watten hé?

S Awel zo de dingen die da je allemaal doet als ge thuis zijt?

P Ah awel ja ten euh … Kben in dingen in bond van KVLV voor de vrouwenbond. [S: Ja]
Awel doe goen wij noa alle.. regelmoetig in de winter goen we no vergeiringen no toe hé. Tis veel kooklessen en vergeiringen ja kan neki een gezondheidsles zijn oek. Doe goan we allemaan no toe hé. [S: Ja] Dan goen we me de vrouwen van de geburen. (lacht) En dan van de Landelijke Gilde das van de mannen. Doe goen we oek na toe. Goen we. Alst er iets te doen is, allez, goan we doer na toe hé. [S: Ja] … Goen we… Ze doen zelfs soms reizen oek ma doe, da kan ik nu ni mie pakken. Da doen ik ni mier.

S De reizen?

P Azu neki na ne.., me nen bus ieves no [S: Uhu] toe goen of iet hé. Mo da kannik ni mie.

S En hoe kommet?

P Omda ja, pff, dak vermoeid ben en dak azu ni mee-.. en kben oek wel vriee veel kort, kort op mijnen adem hé. [S: Ja?] Neig kort. Als ik een inspanning gedoen hem dak vrie kort op mijne adem hem. Da’s woe zeh. Doe mee hemmik compasse.

S Dus tis te lastig voor u geworden, letterlijk?

P Mmhh, dade toch.

S Ja, het heeft niet te maken met het feit da je niet mee kunt doen door dat het juist dialyse is?

P Niejenienieje

S Tis omdat ge vermoeid zijt nadien? [P: Jaja] Ja en uwen adem?

P Ja mijnen adem.

S Andere sociale dingen diede doet? Zit ge veel op bezoek?

P Ahjamaja de geburen [S: Ja] komen allemoel regelmoetig nog nekie af en ofwel goen wij nekie aldoer. Ge weet. We zitten ni overien azu hé, ge weet. Ni in de huizen goan zitten, da ni hé. [S: Neh] Mor wel tegenien babbelen en da wel allemoel hé.

S Ja.

P Dade… (lange stilte, lacht ongemakkelijk) Ja jong. Azo vertellen dan moe voeisj hé.

S Ja. Euhm gij.. Kep den indruk dade zeer veel belang hecht aan zo sociale contacten hé?

P Ahja? Baja kem toch nog geiren miensjken [S: mensen] woe da ge mee kunt klappen.

S Ja en vinde da dat een pro.. zo da u dialyse daar zo

--Opname onderbreekt—

S Ja nu ist terug verder aant doen. [P: Ja] Als ze bellen dan stopt ie me opnemen. [P: Ahnja] Wa waren we aant zeggen? Kep in de laatste zin gemist. Wa waarde aant zeggen?

P Na weet ik het ni mier.

S Kweet ook ni. (patiënt lacht) Thad iets me euhmmm u sociaal leven te maken, nog graag mensen
[P: Jaja] Ah ja dak zei dat geen probleem was van de dialyse dat dat daar zo.. tussenkomt. En dan zegt ge ja en dan waren.. vertel maar.

P Groh ja kweet kik doe ni mie zoviel uuver, (lacht) da weet ik. Ma gewoen ze kommen zelle bij een en vertellen wij doer over nog een beke en zelle vroegen dan nog een beke hé, ge kunt dat hé. En dien azu en dien azu. (gsm trilt) Get dan oek mensjken da da oek kunnen, da da allemoel meegemaakt heet. Wemmen ne gebuur gehad hé, dienen minschj beuven ons doe hé, dienen heeft vijfentwintig joer na de dialyse geweest, vijfentwintig joer.. Ma da was noar Oiljst,. Hij is oud geworden hé, is em. Vijfentwintig joer.

S Das lang hé?

P Joet. (lange stilte) Jejoet.

S Ma ge kent dan nog andere mensen ook [P: Majoek] da aan dialyse zijn?

P Majoek. Jajoek, das een dialyse. Ma die gasten die tèn… Ma kem er hier al viel geweten die gestorven zijn oek zeh.

S Ja hé.

P Neig vele. Ooeh kem hier al, ja een pak gezien zeh.

S En hebde daar dan ook verdriet van?

P Joak. Da pakt mij. Da pakt mij. De leste da gestorven is, die lag doe tegen mij zie. Da was nog ne jonge mensj zeh. Joak, doe..doe hem ik ertzier van. Doe.. doe weet ik.

S En ik kan – (patiënt onderbreekt)

P Kort twie hé! Oeehhh. Doe weet ik het. (korte stilte) Doe kan ik ni goe tegen, maja. (mompelt tegen zichzelf) Ge moet het verzetten hé.

S Ja. [P: Moeije] En praat ge daar dan over met de andere mensen van de dialyse soms?

P Joajejoag, jejoagjejoag (korte stilte) Das zeker.

S Want ik kan mij voorstellen dat da.. dat da lastig euh..

P (fluistert) Kem er hier al veel geweten oek zeh. Twas nu wel een stokoud miensjken maja,
joeng miensjken oek. (fluistert) Kem er hier al veel weten te goan.. (lange stilte)
Ja joeng, mo doe moeije ni mee bezig zijn hé. [S: Nee] Doe moogde ni mee bezig, ge moet het aan de goeie kant pakken. Da moeije altijd zeggen, euh da kan ma nog redden, da kan ma nog helpen. Ja moenekik de goeie kant pakken en [S: Ja] verdien a corroge pakken veu te blijven kommen hé. Ja..

S En moeste, moest ge zo één ietske in u leven kunnen veranderen: zou da de dialyse zijn of zou ge iets anders veranderen?

(lange stilte)

S Ge hebt zo één wens en ge moogt één ding aan u leven…

(lange stilte)

P Ahbajajoak das as..as..as.. ge da zou kunnen afschaffen maja, [S: Nee] das neuijt ni woe hé.

S Nee da ga nooit int echt gaan hé. [P: Da ga neujit ni goen] Maar moest ge een wens mogen doen. Zegt ge van ik zou da weg wensen of ik zou voor iets anders wensen of..?

(lange stilte)

P Dade.. Awe..Awel in dinge in aanmerking voor een nier kommek ni hé. [S: Ja] (patiënt mompelt) maja zemme mij da van zen leven al gevraagd en gedoen met dak een dingen ben.. een diabeet hé. [S: Uhu] Is da ieves ewa moeilijk. En zemme liever van niet. [S: Ja] Als wel. As ten azu goe is, ist ten azu hé. (zucht en mompelt)

S En was da lastig, als ze zo zeiden van da ge geen nier ging kunnen krijgen nooit?

P (korte stilte) Awel ja.. Aan den iene kant wel en aan den andere kant nie hé. Ja..

S Hoe bedoelde?

P Awel ja aan den iene kant zou ge gered zijn. Als ten lukt! [S: Ja] Ma het kan anders zijn oek hé. [S: Ja] Het kan mislukken oek hé. Doemee zijje oek allemaal bezig hé ten. (korte stilte) Mo ik trek het mij nog zo neig ni oen. Moeten wijle kommen van … Tis wel veel te kommen en lang te kommen mo ja..

S Tis de afweging da de maakt ten opzichte van het alternatief?

P Ja .

S Ja.

(lange stilte)

S Goed… Hebde gij ooit al gehoord van “Kt/V”?

P Nienk, wa is dade?

S Euhm, das niets belangrijk ze. Das iets medisch, dat gebruiken wij om die goedheid van dialyse zo in de.. in medische wereld enzo, gebruiken we dat om te kijken hoe goed dat u dailyse marcheert. Maja de mensen gebruiken da ni natuurlijk hé. (korte stilte) En de prof gebruikt het ook nooit dus euh..

P Ahja.

S Dan gaat ge het niet kennen hé denk ik. Als ze het u nooit verteld heeft.

P Ja.

S Neh. Oké, ik denk dat we ongeveer rond zijn zeker hé?

P Ja.

S Ja. Merci alleszins voor u tijd.

(patiënt lacht)

S Tis altijd een keer interessant om keer te luisteren wa de mensen vinden hé. [P: Ahja] Wacht ik ga dat hier eerst. –Opname stopt-

Interview Patiënt 11

**S**  Hebde gij euhm da papier gekregen me zo dienen uitleg op? (korte stilte) Wete nog wa er allemaal opstond?

**P** Ni afgelezen.

**S** Ge hebt het ni afgelezen?

**P** Daarveu heb kik gienen tijd.

**S**  Hebde genen tijd? Moek het nog kort vertellen? (korte stilte) Neh. Oké. Het komt erop neer gewoon da alles euhm da we al u gegevens goe behandelen hé. Da we da ni weggeven en da we wa informatie willen over dialyse en of de dan daarover ne keer me ons wilt babbelen. Ma kzou eerst nekeer willen weten hoe dade gij aan de dialyse ge.. terecht gekomen zijt, want ik ken uwen dossier niet hé.

**P** (korte stilte) Om.. Omda.. Omdak vo.. vocht ophiele.

**S** Ja en dus u nieren werkten ni meer dan? En wete hoe dat da kwam?

(korte stilte)

**S** Hebben ze u da nooit verteld? … Neh. En hoe gaat dat de dialyse?

(lange stilte)

**P** Goed, maar ambetant hé. Da ge a werk moe loeten stoen.

**S** Hebde zo’n.. Hebde een druk leven?

**P**  Hmmjaa.

**S** Ja. En wa moete allemaal opgeven dan?

**P**  (korte stilte) ’S nachts veel meer werken hé.

**S**  ’S nachts?!

**P**  Ahja. Wad k anders in de veurmiddag doen, moek nu ‘s nachts doen.

**S**  En kunde nekeer vertellen wa da gij allemaal zo doet dan?

**P** Ben landbouwer.

**S** Aahnn.

**P** Ik volg het seizoen.

**S**  Ja.

**P** A we mais planten of euhh bieten planten.

**S**  Ja.

(lange stilte)

**P**  Kunde ni zeggen kk.. kgoen het noste week doen hé.

**S**  Neh.

**P**  Et moe gedoen zijn he.

**S**  En hebde wa hulp?

**P** Bwa. Mijn vader helpt nog.

**S**  Ja.

**P**  Wa dat hem nog kan.

**S** Ja en lukt da dan?

**P** Hah. (korte stilte) Op een giel jeur goek veuren te negenenhalf tsnaks gaak ni sloapen.

**S** En zijde dan ni doodmoe?

**P**  Hah. Kommekik allie voor te sloapen hé.

**S**  Ja, ne keer vier uur u ogen dicht doen.

**P**  Ja vier uren kan da ni, dan moete ze awen bloeddruk pakken en of wa ist altijd iet.

**S**  Ja of dan komen ze interviews afnemen. (zucht) Ja.. Euhm (klikt met tong) De.. Het onderzoek gaat een beetje over “Adequacy of dialysis”. Da’s een Engelse term hé. Dat wilt zeggen.

P Da verstoa ik ni.

**S** Ja, awel.

**P**  Kan geen Engels.

**S**  In’t Nederlands is het ook nog altijd moeilijk, dan zeggen ze eerst “adequaatheid van dialyse”. Ma das ook nog ne moeilijken term hé, maar als we het anders zeggen is “goeie dialyse”. En dus de vraag is een beetje: “Wat is da een goeie dialyse voor u?”.

(lange stilte – alarm dialysetoestel)

**S**  Wa maakt er voor u dat de dialyse goe is?

**P**  Dat de verpleegsters me a voeten ni meugen spelen.

**S**  Doen ze da?

**P** Baja sommigsten.

**S**  En hoeda?

**P** Ze… (korte stilte) Als ze wat zegt en.. en.. ek ben ni gruuit. Ge zijt een klein kind. Bekijkt ne keer in de spiegel. Dan gajet zien.

**S** Oei.

**P** Ma bij mij goat da der ni in hé. De die heet gedoen en voor altijd. (korte stilte) En ze weet het wel!

**S** Ja. Tis een beetje … de verpleegkundigen enzo? Das voor u belangrijk voor een dialyse?

(Dialyse toestel in alarm)

**P**  Ma ben aant reclammeren omda het stopt.

**S** Uhu.

**P** Ma Betty azu kan kik ni liggen!

**P** En in de zetel koste kik alles, mo hier ni, op da bedde.

(lange stilte)

**P**  Wel had mijne zetel hier gezet hé.

**S** Tspijt mij zeh. Kwist het ook ni.

**P** Baja da… Baja das a foute ni hé? Ahn. Ofk vrage mij af waarom da ni doen, als da bedde ni gemakkelijk ligt, die zetel alhier ni brengen.

**S** Ik ken de politiek ni van het ziekenhuis..

**P** Ahjamaja.

(lange stilte)

**S** Dus ne zetel is voor u ook waarschijnlijk belangrijk voor ne goeie dialyse?

(korte stilte)

**S** Ja. En zijn er andere zaken diede belangrijk vindt?

(lange stilte)

**S** Zijn er andere dingen die je zegt van da maakt mijn dialyse beter of slechter?

**P** Neuje.

(lange stilte)

**S**  Moede gij veel vocht af altijd of ni?

**P**  (mompelt onverstaanbaar da machien.

**S**  In totaal?

**P** Nenieje per uure.

**S** Per uur? Ja.

(lange stilte)

**S** En hebde ooit al gehad da ge langer moe blijven?

**P** Nieje.

**S**  Altijd 4 uur?

**P**  Ma kzou ni blijven oek zeh! Kzou het kot afbreken!

**S**  Awel das de vraag da ik zou stellen derachter. Ja.

(korte stilte)

**S** En heeft het te maken met het feit da ge eigenlijk moe werken?.. Ja.

(lange stilte)

**S** En euhmm.. Alsde.. Moeste iets kunnen veranderen aan de dialyse, wa zou je der aan aanpassen?

**P** Da weet ik ni.

(korte stilte)

**S**  Ook geen gemakkelijke vraag hé.

(korte stilte)

**S**  En hebde zo nen dag dan bijvoorbeeld da je zegt van da was.. de beste dialyse dag ooit?

**P** Nieje.

**S** Neh. Wa was de slechtste dialyse dag ooit? … Zijn er dingen die u dialyse slecht maken? Behalve dan bepaalde verpleegkundigen blijkbaar.

**P** De ien verpleegster is goed, de andere maakt u uit dak et a gezegd hem.

**S** Ja.

**P** Dan zijn erbij da reclammeren als als ge vijf minuten of tien minuten te loet zet. Ma ge meugt ni vergeten, ik kan ni op uure zijn! Alster mergenvroeg een koe kalft..

**S**  Ja?

**P**  Kan kik.. euh Ni langs hier kommen en men koe loeten stoen, want alsek thuis komme smiddags kan mijn koe en mijn kalf doed zijn. En wie gaat er mij da voergoeien?

**S** Ja. Komde zelf naar hier?

**P** Jajaak.

**S** Ja. Om een beetje meer u tijd te kunnen in euh.. En hebde.. En.. Dus ge zei als er een kalf is of zo ietske is, ge zijt den enigste die het kan doen?

**P**  Mijn vader is vijfenzeventig joer!

**S** Ja en hebde een vrouw of kinders?

**P** Nieje.

**S**  Nee.

**P** En hij heeft nooit aan de koeien gewerkt mijn pa, hij heeft altijd weest werken. Ik doe veut aant bedrijf van mijn gruutouders.

**S** Ja, u pa is nooit een.. de landbouwer geweest eigenlijk?

**P** Bwaneek.

**S**  En doedet graag?

**P** Gro ot et giene gedoen, kzou het ni gedoen hebben hé.

**S** Euh, ma doede u werk graag?

**P** Groh ja.

**S** Op zich wel? Ma tis moeilijk te combineren met de dialys.

**P** Tis gemakkelijk, ma euuhh… Als gelijk veurige week oek.. Ge komt toe tien minuten loeter as anders (korte stilte) Twas direct al.. reclammeren.

**S** Ge vindt dat er niet veel begrip is?

**P** Mmhh.. (korte stilte) Ma ge meugt ni vergeten een koe en een kalf hé, is vijftienhonderd euro.

**S** Das veel hé.

**P**  Njah.. Da kank kwet zen. Azu zouk rap in de nesten zitten me annen deisel.

**S** Wadde?

**P** As ik… As ek veuk dak opwas veurt goe, zitte in de nesten me an deisel. Kweurk langs ier zeh.

**S** Ja (lacht) Ik ben ni van hier hé.

**P** Ahnee ahja.

(lange stilte)

**P**  Ma bepoelde verpleegsters hebben hier respect veu mo alles da poeiten heeft!

(Dialyse toestel gaat in alarm)

**S** Tis terug aant piepen.

(Dialyse alarm gaat uit)

**S** Ah tis over. Ja, stoort u da ni die alarmen?

**P** Da benk al geweun.

**S** Tis vooral hoe da ze me u omgaan? Das voor u het belangrijkste? En voor de rest… zo snel mogelijk terug weg blijkbaar. Ja.. Oké. En zijde gij al lang aan dialyse?

**P** Vijf joer peisk.

**S**  En merkte het verschil met dialyse of zonder?

**P** Nieje.

**S** Voelde gij u soms na dialyse of..?

**P**  Nieje.

**S**  Of zelf beter?

**P** Nieje.

**S** Merkt ge da de dialyse iets doet?

**P** Baja. Ah ik ier ni kom, worde men bienen hiel opgewollen.. Vant vocht dak ophiele.

**S** Ja.

**P** En da krijg ik noa ni meer hé.

**S**  Zo kunde gij zien dat het werkt?

**P** Njaa.

**S** Ja. (korte stilte) Oké en voor de rest.. Hebde nog ander opmerkingen over dialyse bijvoorbeeld?

**P** Nieje.

(lange stilte)

**S** Wete nog int begin.. As ge dialyse had, was da een grote aanpassing? Hebde daar een tijd mee… Want nu is da al vijf jaar natuurlijk, maar die eerste maanden? Kunde u da nog voorstellen hoe dat dat was voor u?

**P** Nieje.

**S** Ja. Hebde gij ooit ne katheter gehad?

**P** Jeujk.

**S** En sinds wanneer hebde ne fistel?

**P** Groh, noe een moind.

**S** Hmm?

**P** (mompelt iets onverstaanbaar 09:01)

**S** Ah nog maar net?

**P** Nieje, noa een moand of twie nadien pas.

**S** Ah een ma.. een maand of twee. Ja oké. Maakt da voor u een verschil uit katheter of … fistel?

**P** Die fistel da marcheert ni hé.

**S** Nee?

**P** Ge weet zelve, ge loept giele dag tussen a biesten. Ge l… Ge k.. Ge krijgt van .. van hier nen douw

**S** Ja?

**P** Een wreve van ginder en tan wast al verzopen.

**S** Ma nu ist goe?

**P** Maja mo me a katheter willek emmen.

**S**  Ah u katheter ging ni?

**P** Jaa.

**S** Ahjaja zo. Dus ge zijt content met uwen fistel en minder (dialyse alarm gaat af)

**P** Ahja.

**S** .. en minder problemen ja. Tis ook ja, dat is ook effectief het risico hé meestal met infecties.

(Dialyse toestel blijft alarm geven)

**S** Goed? Kzou der op duwen moest ik weten waar da ik moe aankomen.

**P** Ik weet het niet. Ik weet het niet.

**S** Ik weet het ook niet. Oké goe, denk dat ik al mijn vragen al gesteld heb. Merci alleszins voor uw tijd en het spijt mij dade gij in euh.. in een bed hebt moeten liggen hé. Volgende keer terug op de zetel zeg en veel succes met uw seizoen. Is het ni bijna gedaan? … Nee?

**P** Tis giel tjoer.

**S** En in de winter ist ni kalmer?

**P** Kalmer?! Tan emme we viel mier werk, komen de biesten op stal.

**S** Aaahnn.. Ik zou denken int.. Int s.. int groeiseizoen meer en in de winter een beetje goedkomen.

**P** Tis afwisselend werk hé.

**S** Ah ma tis omda de twee dingen doet.

**P** Ja.

**S** Ge doet en…en ja… En beesten en planten. Wacht hé kga eerst een keer stoppen. En ge plant voor voor voeder ofzo? Of ge plant ook voor euh…

- OPNAME STOPT -
